# Prognostic signature based on mitochondria quality control proteins for the prediction of lung adenocarcinoma patients survival

**DOI:** 10.1038/s41420-023-01649-x

**Published:** 2023-09-25

**Authors:** Anna S. Gorbunova, Alexey V. Zamaraev, Maria A. Yapryntseva, Olga V. Kovaleva, Elena M. Tchevkina, Maria V. Turkina, Boris Zhivotovsky, Gelina S. Kopeina

**Affiliations:** 1 Faculty of Medicine, MV Lomonosov Moscow State University, 119991 Moscow, Russia; 2 Engelhardt Institute of Molecular Biology, Russian Academy of Sciences, 119991 Moscow, Russia; 3 Department of Oncogenes Regulation, NN Blokhin Medical Research Center of Oncology, 115478 Moscow, Russia; 4 Faculty of Medicine and Heath Sciences, Department of Clinical and Experimental Medicine, Linköpings Universitet, 58185 Linkoping, Sweden; 5 Division of Toxicology, Institute of Environmental Medicine, Karolinska Institutet, 17177 Stockholm, Sweden

**Keywords:** Non-small-cell lung cancer, Apoptosis

## Abstract

Lung cancer is the leading cause of cancer mortality worldwide. In recent years, the incidence of lung cancer subtype lung adenocarcinoma (LUAD) has steadily increased. Mitochondria, as a pivotal site of cell bioenergetics, metabolism, cell signaling, and cell death, are often dysregulated in lung cancer cells. Mitochondria maintenance and integrity depend on mitochondrial quality control proteins (MQCPs). During lung cancer progression, the levels of MQCPs could change and promote cancer cell adaptation to the microenvironment and stresses. Here, univariate and multivariate proportional Cox regression analyses were applied to develop a signature based on the level of MQCPs (dimeric form of BNIP3, DRP1, and SIRT3) in tumorous and non-tumorous samples of 80 patients with LUAD. The MQCP signature could be used to separate the patients with LUAD into high- and low-risk groups. Survival analysis indicated that patients in the high-risk group had dramatically shorter overall survival compared with the low-risk patients. Moreover, a nomogram combining clinicopathologic features and the MQCP signature was constructed and validated to predict 1-, 3-, and 5-year overall survival of the patients. Thus, this study presents a novel signature based on MQCPs as a reliable prognostic tool to predict overall survival for patients with LUAD.

## Introduction

Currently, lung cancer is the most common cause of morbidity and mortality among all cancer types in both sexes worldwide [1]. Lung cancer has histologically been subdivided into small-cell lung cancer (SCLC) and non-small-cell lung cancer (NSCLC). Lung adenocarcinoma (LUAD) is an NSCLC subtype that is more common in non-smokers. LUAD is mainly caused by somatic mutations in *KRAS*, *EGFR*, *ALK*, *ERBB2*, and *BRAF*, and by chronic inflammation. Despite being well studied, there are still many gaps regarding the prerequisites of LUAD.

**Fig. 1:**
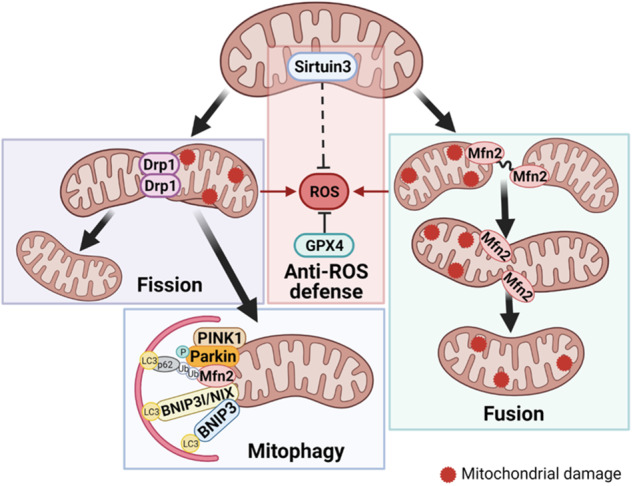
Interrelations among PINK1, Parkin, BNIP3, BNIP3L/NIX, DRP1, MFN2, SIRT3, and GPX4. Thick solid arrows show the action of mitochondrial dynamics, thin solid lines demonstrate the impact on ROS production, and dotted lines indicate indirect influence of the ROS level. Made in Biorender.

In lungs, mitochondria have a strong impact on tissue function and homeostasis [2]. Mitochondrial dysfunction may trigger pathologic processes in the lung, including chronic diseases, and cancer [3–5]. Proper mitochondrial functioning depends on the quality of these intracellular compartments, which is maintained by mitophagy (mitochondrial autophagy) and organelle dynamics (fission and fusion). Mitophagy proteins (BNIP3, BNIP3L/NIX, PINK1, and Parkin) engage autophagosomes to dysfunctional mitochondria, which eventually leads to their degradation. Fission proteins, including the key regulator DRP1, help to segregate damaged mitochondria that will be digested by the mitophagy machinery. Fusion proteins, in particular MFN2, serve to dilute impaired mitochondrial components, preventing their removal. Generally, mitophagy and mitochondrial dynamics maintain a healthy mitochondrial population and prevent reactive oxygen species (ROS) accumulation. Therefore, proteins related to ROS regulation were included in the study. One of these proteins, Sirtuin 3 (SIRT3), is a mitochondrial deacetylase and a crucial regulator of oxidative stress [6]. Of note, mitochondrial metabolism and oxidative stress are closely related to ferroptosis. Glutathione peroxidase 4 (GPX4) is involved in ferroptosis regulation and in protecting mitochondria from oxidative damage [7]. Besides, the protein levels of GPX4 and SIRT3 have not yet been fully estimated thoroughly in patients with LUAD [8–12]. Thus, the key regulators of mitophagy (PINK1, Parkin, BNIP3, and BNIP3L/NIX), mitochondrial fission (DRP1) and fusion (MFN2), and antioxidative defense (SIRT3 and GPX4), which are interrelated among each other (Fig. 1), were included in the analysis.

Besides the molecular background, many physiological factors such as age, sex, and stage, among others, affect disease progression and survival. However, not all of them clearly demonstrate prognostic value for patients with cancer. Hence, identification of prognostic markers associated with improved survival remains a challenge. A possible solution to create a reliable prognostic model is to combine clinicopathologic and molecular traits.

In this study, the impact of mitochondrial quality control proteins (MQCPs) and clinicopathological traits on the survival probability of 80 patients with LUAD was analyzed retrospectively. A prognostic signature based on the level of MQCPs (dimeric form of BNIP3, DRP1, and SIRT3) was developed to predict the overall survival (OS) of patients with LUAD and to divide them into high- and low-risk groups. In addition, a nomogram with an MQCP signature and clinicopathologic traits was established and validated to predict the 1-, 3-, and 5-year OS of the patients.

## Results

### The baseline clinicopathological characteristics of patients with LUAD enrolled in this study

**Fig. 2:**
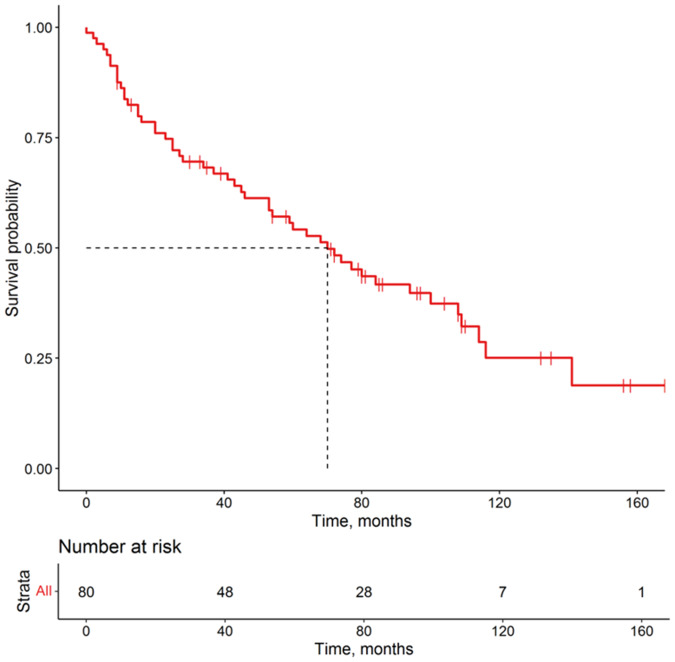
Survival curve of patients with LUAD enrolled in this study. The number of patients at risk is listed below. Red pluses represent censored data.

A total of 80 patients were enrolled after surgical resection of LUAD during the period of 2005–2018. The median age of the included patients was 63 years, and 64% were male. The median follow-up time was 56 months and there were 50 deaths and 30 censored cases (Table 1). The Kaplan–Meier survival curve of the patients with LUAD is shown in Fig. 2. Median OS was 70 months. The 1-, 3-, and 5-year OS rates were 82.4%, 68.2%, and 54.2%, respectively.

**Table 1:** The clinicopathological characteristics of 80 patients with LUAD.

Characteristic	*N* = 80^a^
Follow-up time (months)	56 (20, 94)
Survival status	
Alive	30 (38%)
Dead	50 (62%)
Age	
≤65	50 (62%)
>65	30 (38%)
Gender	
Female	29 (36%)
Male	51 (64%)
*T*	
1–2	54 (68%)
3–4	26 (32%)
*N*	
0	47 (59%)
1–2	33 (41%)
*M*	
0	77 (96.2%)
1	3 (3.8%)
Stage	
1–2	50 (62%)
3–4	30 (38%)
Grade	
1–2	45 (56%)
Mixed	11 (14%)
3–4	24 (30%)
Multiple primary malignant tumors	
Absence	74 (92.5%)
Presence	6 (7.5%)

^a^Median (IQR); *n* (%).

### Comparative analysis of MQCP levels in tumorous and adjacent non-tumorous tissues

**Fig. 3:**
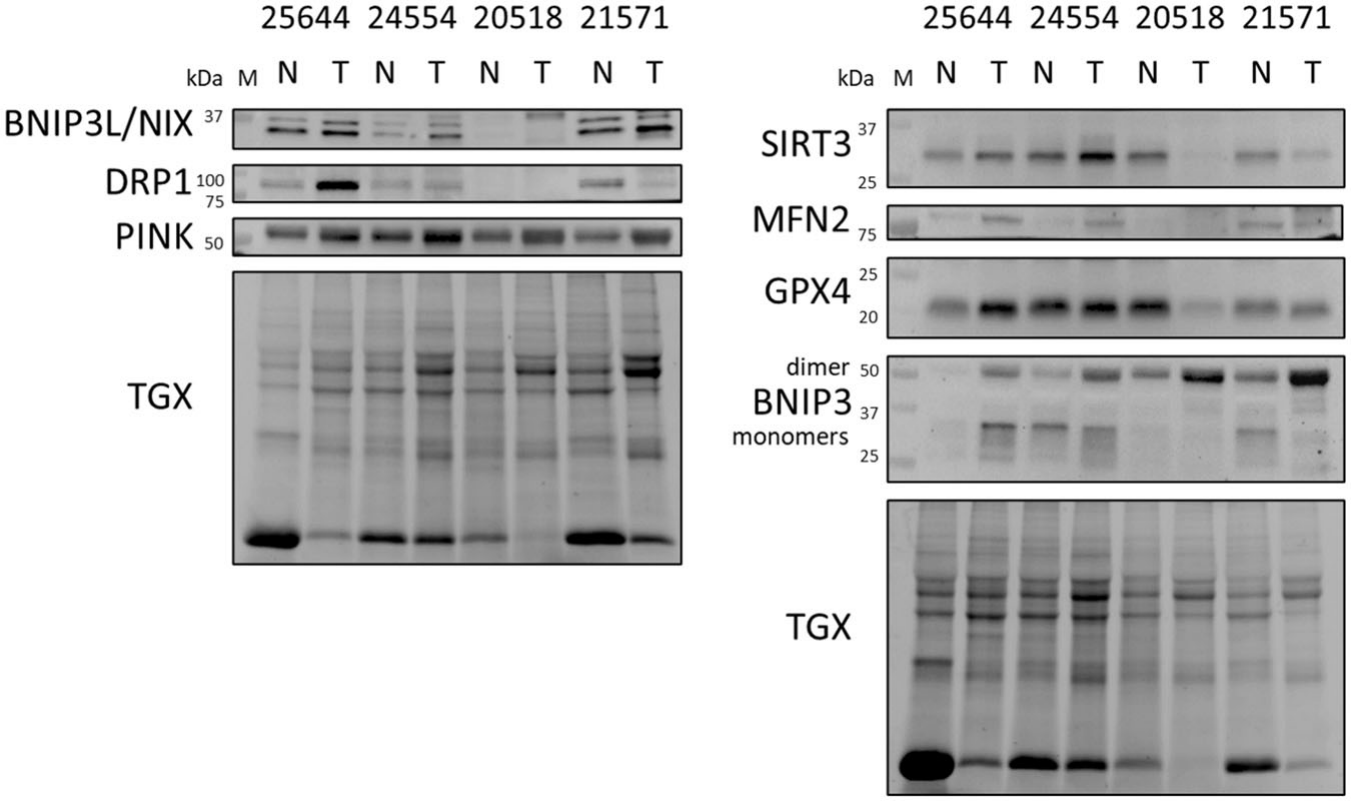
Representative WB analysis of LUAD tissue samples. Note: 25644, 24554, 20518, and 21571 are patient IDs. N, adjacent non-tumorous tissue; T, tumorous tissue; TGX, loading control, TGX Stain-Free^TM^ FastCast^TM^ Gel.

Western blotting (WB) was used to estimate the MQCP levels in tumorous and adjacent non-tumorous tissues of 80 patients with LUAD (Fig. 3 and Suppl. Fig. S1). The current analysis included the key regulators of mitophagy (PINK1, BNIP3, and BNIP3L/NIX), mitochondrial fission (DRP1), mitochondrial fusion (MFN2), and antioxidative defense (SIRT3 and GPX4).

**Fig. 4:**
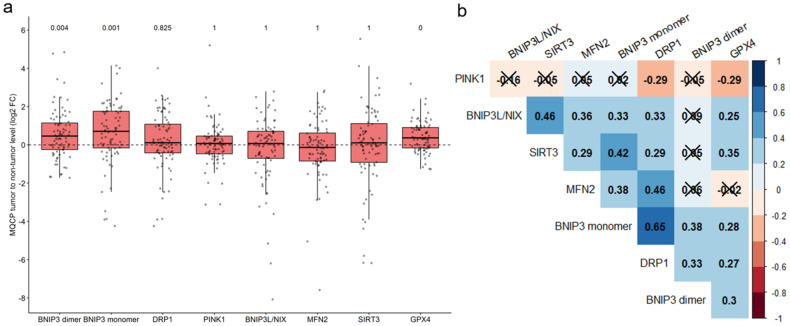
Comparative analysis of MQCP levels in tumorous and adjacent non-tumorous tissues of patients with LUAD. **a** Densitometry analysis of BNIP3 (dimeric and monomeric forms), DRP1, PINK1, BNIP3L/NIX, MFN2, SIRT3, and GPX4 levels in tumorous relative to non-tumorous samples normalized based on the total protein level. Notes: the log2 values were determined as TGX normalized protein level in tumorous samples/TGX normalized protein level in non-tumorous sample. The *p*-values are presented above the corresponding boxplots. **b** Pairwise correlation heatmap among log2 fold change of BNIP3 (dimeric and monomeric forms), DRP1, PINK1, BNIP3L/NIX, MFN2, SIRT3, and GPX4. Note: strikethrough values have *p* > 0.05. *N* = 80 patients.

Upon densitometric analysis of WB data, MQCP levels were normalized based on the total protein levels for each sample quantified by using TGX stain-free gels. The ratio of normalized MQCP levels in tumorous to non-tumorous tissues was transformed into a log2 scale (log2 fold change [FC]) and used for further analysis (Suppl. Table S1). The distribution of log2 fold-changes of MQCPs, shown in Fig. 4a, demonstrated that the levels of BNIP3 dimer (*p* ≤ 0.002) and monomer (*p* ≤ 0.001) and of GPX4 (*p* ≤ 0.001) were significantly increased in tumorous compared with non-tumorous tissues. It is important to note that for several proteins the tendency to increase the level remains not only at the protein level, but also at the level of mRNA (Suppl. Fig. S2).

**Fig. 5:**
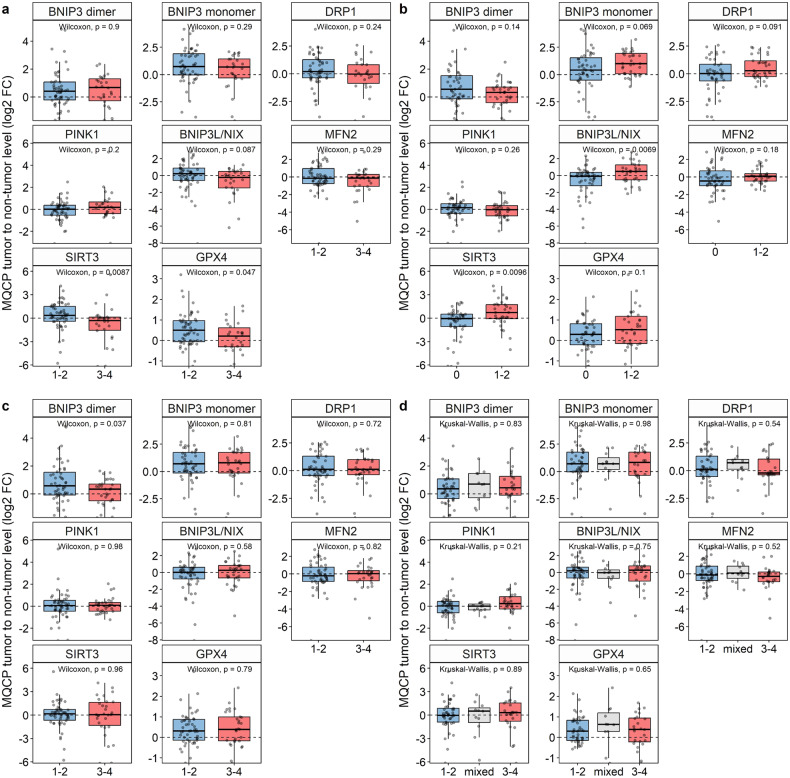
Associations between MQCP levels and clinicopathological features of patients with LUAD. Distribution of log2 fold-changes (FC) of BNIP3 (dimeric and monomeric forms), DRP1, PINK1, BNIP3L/NIX, MFN2, SIRT3, and GPX4, stratified by T (**a**), N (**b**), stage (**c**), and grade (**d**) in 80 patients with LUAD.

Correlation analysis of MQCP log2 fold-changes (FC) revealed a moderate positive correlation between SIRT3, BNIP3L and BNIP3 monomer; and between DRP1, BNIP3 monomer and MFN2 (Fig. 4b). There were weak negative correlations between PINK1 and DRP1 and GPX4. In addition, there were associations between the MQCP log2 FC and the size of the primary tumor (T), regional lymph node involvement (N), stages, and grades (Fig. 5). There was significant upregulation of SIRT3 and GPX4 levels in tumorous tissues in patients with T1–2 (Fig. 5a). In patients with regional lymph node metastasis, the levels of SIRT3 and BNIP3L/NIX were downregulated in tumorous tissues (Fig. 5b). Moreover, increased BNIP3 dimer levels were associated with stage 1–2 tumors (Fig. 5c). The grade was not correlated with any of the proteins (Fig. 5d).

### Development of a prognostic MQCP signature for survival prediction

**Fig. 6:**
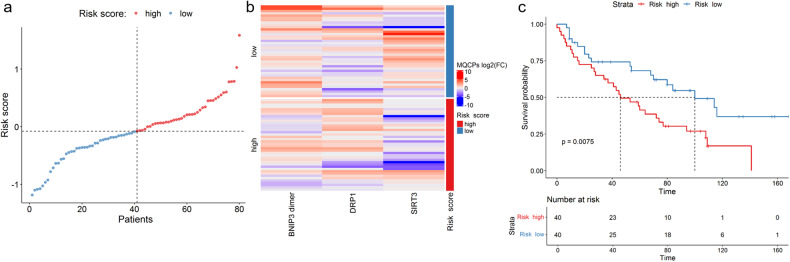
Development of a prognostic MQCP signature for survival prediction. **a** Distribution of risk scores based on a multivariate Cox regression model depending on the BNIP3 dimer, DRP1, and SIRT3 levels. The dotted line represents the median risk scores, dividing patients into the low- and high-risk groups. **b** Heatmap of log2 fold-changes (FC) of BNIP3 dimer, DRP1, and SIRT3 in the low- and high-risk groups. **c** Kaplan–Meier analysis of OS in the low- and high-risk groups. Pluses represent censored data. *N* = 80 patients.

Univariate Cox regression analysis was applied to estimate the prognostic value of the aforementioned MQCPs (Table 2). Bidirectional stepwise regression was used to select prognostic MQCPs and to determine the final multivariate Cox model. SIRT3, DRP1, and BNIP3 dimer were included in the multivariate Cox regression analysis. A deviance residual plot was generated and the Schoenfeld test was performed to examine the influential observations or outliers and time independence of variables (Suppl. Fig. S3). Then, multivariate Cox regression analysis was carried out to construct a risk signature. The risk score equation 0.322*DRP1_log2FC – 0.205*SIRT3_log2FC – 0.265*BNIP3dimer_log2FC was used to calculate the risk scores of each patient. Using the median risk score as a cut-off point, the patients were divided into high-risk (*n* = 40) and low-risk (*n* = 40) groups (Fig. 6a). A heatmap of SIRT3, DRP1, and the BNIP3 dimer and the Kaplan–Meier curve depending on the risk scores were also constructed (Fig. 6b, c). Patients in the high-risk group had a significantly (*p* ≤ 0.0075) shorter OS compared with the low-risk group (Fig. 6c). The median survival of the high-risk group was 46 months, which is 2.2 times less than for patients in the low-risk group (100 months). Thus, alterations in the levels of BNIP3 dimer, DRP1, and SIRT3 in tumorous relative to non-tumorous samples have an impact on OS.

**Table 2:** Univariate and multivariate Cox regression models based on the fold-changes (FC) of BNIP3 (dimeric and monomeric forms), DRP1, PINK1, BNIP3L/NIX, MFN2, SIRT3, and GPX4 of 80 patients with LUAD.

	Univariate				Multivariate			
Characteristic	*N*	HR^a^	95% CI^a^	*p*-value	*N*	HR^a^	95% CI^a^	*p*-value
BNIP3 dimer	80	0.85	0.68, 1.05	0.13	80	0.77	0.60, 0.97	0.030
BNIP3 monomer	80	0.95	0.80, 1.12	0.5				
DRP1	80	1.07	0.89, 1.30	0.5	80	1.38	1.09, 1.75	0.008
PINK1	80	1.11	0.83, 1.48	0.5				
BNIP3L/NIX	80	0.96	0.84, 1.09	0.5				
MFN2	80	1.01	0.85, 1.19	>0.9				
SIRT3	80	0.87	0.76, 0.99	0.035	80	0.81	0.71, 0.94	0.005
GPX4	80	0.82	0.56, 1.19	0.3				

^a^HR hazard ratio, *CI* confidence interval.

Wald test = 14.4; *p*-value(Wald test) = 0.002; No. of obs. = 80; *N* events = 50.0; Concordance = 0.645; SE(concordance) = 0.043.

### Construction and evaluation of a nomogram for OS based on the MQCP risk score

Univariate and multivariate Cox regression analysis was used to evaluate the independent predictive value of the MQCP signature for OS among all available clinicopathological variables. The univariate Cox proportional hazards regression analysis demonstrated that TN, tumor stages, age, and the MQCP risk score (Hazard ratio [HR] = 2.74, *p* ≤ 0.001) were significantly correlated with the survival of patients (Table 3). The risk score remained as an independent prognostic predictor in the multivariate analyses after adjusting for other clinicopathological variables (HR = 3.88, *p* ≤ 0.001).

**Table 3:** Univariate and multivariate Cox regression analyses of clinicopathological variables and the MQCP risk score based on OS in 80 patients with LUAD.

	Univariate				Multivariate				Multivariate (reduced)			
Characteristic	*N*	HR^a^	95% CI^a^	*p*-value	*N*	HR^a^	95% CI^a^	*p*-value	*N*	HR^a^	95% CI^a^	*p*-value
*T*												
1–2	54	—	—		54	—	—					
3–4	26	2.06	1.13, 3.79	0.019	26	2.16	0.95, 4.90	0.065				
*N*												
0	47	—	—		47	—	—					
1–2	33	1.74	0.99, 3.05	0.055	33	1.08	0.48, 2.46	0.9				
Grade												
1–2	45	—	—		45	—	—					
Mixed	11	2.04	0.97, 4.32	0.061	11	1.55	0.64, 3.71	0.3				
3–4	24	1.95	1.03, 3.70	0.041	24	1.70	0.85, 3.38	0.13				
Stage												
1–2	50	—	—		50	—	—		50	—	—	
3–4	30	2.56	1.46, 4.49	0.001	30	3.04	1.20, 7.69	0.019	30	4.00	2.18, 7.36	<0.001
Sex												
Female	29	—	—		29	—	—					
Male	51	1.07	0.60, 1.90	0.8	51	0.78	0.37, 1.63	0.5				
Age												
≤65	50	—	—		50	—	—		50	—	—	
>65	30	1.69	0.97, 2.96	0.065	30	3.38	1.65, 6.93	<0.001	30	3.02	1.65, 5.53	<0.001
Multiple primary malignant tumors												
Absence	74	—	—		74	—	—					
Presence	6	1.95	0.69, 5.47	0.2	6	0.75	0.20, 2.76	0.7				
Risk score	80	2.74	1.63, 4.61	<0.001	80	4.03	2.01, 8.10	<0.001	80	3.68	1.99, 6.81	<0.001

^a^*HR* hazard ratio, *CI* confidence interval.

Wald test = 40.4; *p*-value(Wald test) = 0.000; No. of obs. = 80; *N* events = 50.0; Concordance = 0.747; SE (Concordance) = 0.037.

**Fig. 7:**
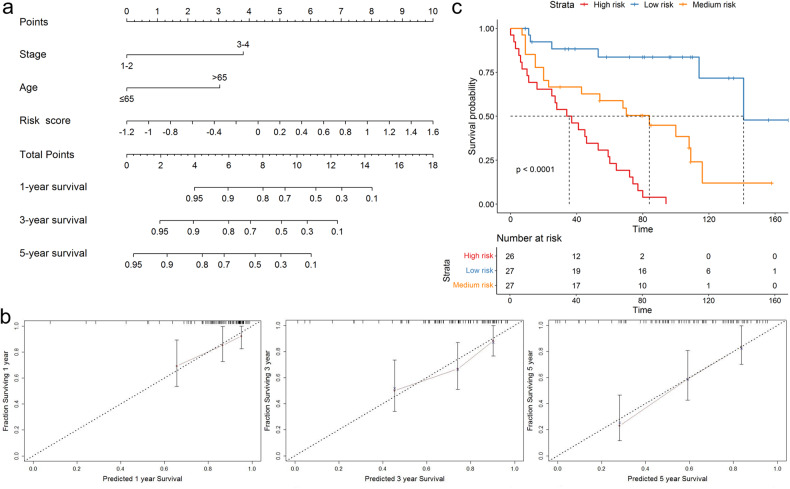
Construction and validation of the prognostic nomogram. **a** The prognostic nomogram for predicting 1-, 3-, and 5-year OS of patients with LUAD. **b** The bootstrapped calibration curves for 1-, 3-, and 5-years OS. **c** Kaplan–Meier curves of OS in the low-, medium- and high-risk groups. Pluses represent censored data. *N* = 80 patients.

To construct a suitable tool for clinical application, a prognostic nomogram to predict 1-, 3-, and 5-year OS using the MQCP prognostic signature and the independent clinicopathological factors such as stage and age was established (Fig. 7a). The Harrell’s concordance index (C-index), which reflects the differentiation ability and accuracy of the prediction model, for the tumor stage model and the nomogram was 0.628 (standard error [SE] = 0.036) and 0.749 (SE = 0.037), respectively (Suppl. Fig. S4). The discriminability of regression models was cross-validated using the bootstrap re-sampling method. The bootstrapped calibration curve demonstrated good predictive accuracy between nomogram predictions and actual observations for 1-, 3-, and 5-year OS probabilities (Fig. 7b). According to the nomogram, the total risk score of each patient was calculated. Using the tertile of the total risk score value, patients were categorized into three groups with a low (*n* = 27), medium (*n* = 27), and high (*n* = 26) risk of death. The Kaplan–Meier curves demonstrated that the survival of these three groups of patients was significantly different from each other (*p* ≤ 0.0001) (Fig. 7c). Median OS of patients in the high-risk group was 35.5 months, which is 2.4 times less than for patients from the medium-risk group (84 months), and 4 times less than in the low-risk group (141 months). Thus, the nomogram for patients with LUAD had considerable discriminative power and good predictive value.

## Discussion

LUAD is an aggressive cancer type with unsatisfactory OS prognosis. To predict the prognosis of patients with LUAD, several clinical factors such as age, grade, TNM, and stage have been well characterized and are used clinically. However, recent studies have demonstrated the crucial role of proteogenomic data in cancer prognosis and guiding treatment decisions. Mitochondrial functioning and metabolism could significantly regulate the development and progression of lung cancer, including LUAD [13, 14]. Mitochondrial quality is modulated by mitophagy and organelle dynamics [15]. Modulation of mitophagy and mitochondrial dynamics provides tumor cells, particularly in LUAD, advantages to overcome extreme environments, resulting in a poor prognosis for patients [13, 16, 17]. Taking this into account, the levels of MQCPs in tumorous and non-tumorous tissues were estimated—including key regulators of mitophagy BNIP3 (dimeric and monomeric forms), DRP1, PINK1, BNIP3L/NIX, MFN2, SIRT3, and GPX4—to create an MQCP-based signature to aid in the prediction of LUAD patient survival.

Analysis of MQCP levels in tissues from patients with LUAD demonstrated the abundance of BNIP3 dimer and monomer as well as GPX4 in tumorous samples (Fig. 4a). The elevated protein levels of BNIP3 and GPX4 in cancer tissues are concordant with the data from TCGA dataset of 517 LUAD patients (Suppl. Fig. S2). Increased BNIP3 levels may be associated with tumor hypoxia and negative regulation of apoptosis. The development of solid tumors such as LUAD is accompanied by hypoxia due to rapid tumor growth, which outpaces angiogenesis and, as a consequence, leads to a limited oxygen supply. The *BNIP3* promoter contains a hypoxia response element (HRE) that could be activated under hypoxic conditions. Indeed, mutual expression of BNIP3 and HIF-1α has been demonstrated by immunohistochemistry in tissues of patients with NSCLC [18]. Moreover, BNIP3 has a tumorigenic function, suppressing expression of apoptosis-inducing factor (AIF) to prevent apoptotic cell death [19]. This data is in line with the effects of BNIP3 knockout or knockdown in LUAD cell lines. BNIP3 suppression enhances apoptosis in LUAD cell lines compared with wild-type cells under cisplatin induction [20]. Besides, by promoting HRE-containing genes, hypoxia stimulates ROS production, leading to oxidative stress. Antioxidative proteins, in particular, GPX4 are activated to overcome the destructive influence of ROS. GPX4 overexpression hinders oxidative stress–induced cell death in a mouse model that displays GPX4 as a pro-tumorigenic protein [21]. GPX4 knockdown increases apoptosis and inhibits proliferation in non-cancer and cancer cells [22–24]. Therefore, increased GPX4 levels in LUAD cells is possibly linked to excessive ROS production, cell proliferation, and reduced apoptosis compared with non-tumorous tissues.

The levels of most MQCPs positively correlated with each other, showing their joint action in maintaining a healthy mitochondrial population. However, there were two negative correlations: between PINK1 and DRP1 and between PINK1 and GPX4 (Fig. 4b). The negative PINK1–DRP1 correlation can be explained by the interchangeable expression of PINK1 and DRP1 to compensate for each other’s functions. This phenomenon has been shown in a PINK1 knockdown cell line and a DRP1^+/-^ mouse model [25, 26].On the other hand, the negative PINK1–GPX4 correlation can be explained by the activation of PINK-dependent mitophagy during ferroptosis, which is negatively regulated by GPX4 [27].

During tumor progression, the MQCP levels change, promoting cancer cells to adapt to the changing microenvironment. In this regard, associations between the MQCP levels and the size and localization of primary tumor (T), lymph node metastases (N), and tumor stages (Fig. 5) were analyzed. SIRT3 was significantly increased in LUAD patients with T1–2 and N1–2. Consequently, an increased SIRT3 level may characterize metastatic LUAD, whose cells spread to the lymph nodes without forming an extensive primary tumor site. These data are in line with an NSCLC cohort of patients where SIRT3 expression was correlated with lymph node metastasis [8]. Similarly to SIRT3, the BNIP3L/NIX level has a comparable tendency to be elevated in patients with T1–2 (*p* ≤ 0.087) and N1–2 (*p* ≤ 0.0069). Humpton et al. detected an increased BNIP3L/NIX level in the metastases of a pancreatic cancer mouse model [28]. BNIP3L/NIX expression is regulated by the HIF-1a transcription factor, which modulates glycolysis and oxidative stress, stimulating the survival of disseminated cells and metastasis [29].

A univariate Cox regression analysis was conducted to determine the association between MQCP levels and the LUAD prognosis. Bidirectional stepwise regression was used to develop a prognostic MQCP signature that consists of three proteins (DRP1, BNIP3 dimer, and SIRT3). Based on the MQCP signature, the elevated SIRT3 and BNIP3 dimer levels in tumorous tissues were predictive of better survival (Table 2). Previous studies have demonstrated that SIRT3 overexpression enhances apoptosis of lung cancer cells and sensitivity to cisplatin [30]. Thus, the prolonged OS could be associated with the efficacy of postoperative adjuvant chemotherapy. The elevated level of BNIP3 and decreased risk of death for patients with LUAD (Table 2) are concordant with the data for patients with pancreatic cancer. Patients with pancreatic cancer with low BNIP3 expression have a shortened OS [31]. In contrast to SIRT3 and BNIP3, increased DRP1 levels in tumorous tissue were predictive of poor survival (Table 2). Yu et al. demonstrated that DRP1 overexpression correlated with decreased survival in patients with LUAD [32]. DRP1-mediated mitochondrial fission is an essential step for the proper passage of the cell cycle. Inappropriate mitochondrial fragmentation results in replication stress leading to the inhibition of cell division and apoptosis activation [32]. Thus, elevated DRP1 expression may abolish apoptosis in LUAD cells, which can stimulate the growth and formation of a more aggressive tumor, shortening OS.

All patients were divided into high- and low-risk groups based on their MQCP signature risk scores. Patients with LUAD in the high-risk group demonstrated a significantly poorer prognosis than those in the low-risk group. Moreover, the MQCP signature demonstrated the independent predictive value for OS among the available clinicopathological variables. Furthermore, a nomogram combining age, stage, and risk score was established. The calibration curves demonstrated good consistency between the actual and predicted demonstrated a better predictive value than other constructed with tumor stage or risk score individually. It is important to note that WB is an uncommon approach in clinical laboratory practice. However, it allows evaluating posttranslational modifications of proteins, including their dimers, which have a predictive value. This fact serves as a prerequisite for the implementation of WB for diagnostic purposes for patients with cancer. The constructed nomogram based on the MQCP signature could allow physicians to consider new approaches for more effective prediction of OS and to personalize treatment of patients with LUAD.

This study has a few limitations that should be considered. First, a relatively small cohort of patients with LUAD and MQCPs was included in the study. Second, the study was retrospective, and the patient population was heterogeneous, factors that could introduce bias in the obtained results. Third, the lack of an external validation dataset could limit the clinical value of the model. Thus, despite the interesting new correlations summarized in this study, additional work is required to validate the nomogram using clinical samples from other hospitals.

## Materials and methods

### Data and sample collection

Tissue specimens from 80 patients who underwent LUAD resection at the N.N. Blokhin National Medical Research Center of Oncology (NMRCO) during the period of 2005–2018 were used. The samples were collected in accordance with the guidelines issued by the Ethics Committee of the N.N. Blokhin NMRCO (approval No. 04-04-08097). After surgical removal, the specimens were frozen and stored in liquid nitrogen.

The patients’ diagnoses were verified by histopathology. Matched controls were located as far as possible from the tumor and were histologically confirmed to be normal epithelial cells. The tumors were characterized based on the TNM according to the staging classification of the Eighth Edition of the Union for International Cancer Control. The clinicopathological characteristics of patients with LUAD were categorized as follows: the size and extent of the primary tumor (T1–2 and T3–4), metastases in lymph nodes (N0 and N1–2), metastases in distant organs (M0 and M1), grade (1–2, 3–4, and mixed [different grades of primary tumor site]), stage (1–2 and 3–4), age (≤65 years old and >65 years old), gender (male and female), and multiple primary malignant tumors (presence and absence) (Table 1). Data were analyzed anonymously. OS was defined as the interval between surgery and death, or between surgery and the last follow-up for surviving patients.

### WB analysis

First, 0.5 mL of radioimmunoprecipitation assay (RIPA) lysis buffer (50 mM Tris-HCl [pH 7.4], 150 mM NaCl, 2 mM ethylenediaminetetraacetic acid [EDTA], 0.5% SDS, 0.5% sodium deoxycholate, 1% NP-40, phosphatase inhibitor cocktail 2 [Sigma-Aldrich, P5726, USA], and cOmplete™ Protease Inhibitor Cocktail [Sigma-Aldrich, USA]) was added per tissue sample. Next, samples were homogenized using BashingBead Lysis Tubes (Zymo Research, Irvine, CA, USA) and Precellys 24 tissue homogenizer (Bertin Technologies, Montigny-le-Bretonneux, France). Samples were then incubated for 30 min on ice for cell lysis followed by centrifugation (15,000 × *g*, 30 min, +4 °C). Protein samples were prepared and transferred, as described previously [20]. Protein was separated using TGX Stain-Free™ FastCast™ acrylamide kit (Bio-Rad, USA). The following primary antibodies and dilutions were used: BNIP3 1:1000 (Cat. No: 44060), PINK 1:1000 (Cat. No. 6946), Parkin 1:500 and 1:1000 (Cat. No. 4211), DRP1 1:1000 (Cat No. 8570), and SIRT3 1:1000 (Cat. No. 2627), all from Cell Signaling Technology (CST) (Danvers, MA, USA); BNIP3L/NIX 1:1000 (Cat No. 8399), MFN2 1:1000 (Cat No. 124773), and GPX4 1:1000 (Cat No. 125066), all from Abcam, UK; and Parkin 1:500, 1:1000, and 1:2000 (Cat No. AB5112, Millipore, USA). The following secondary antibodies were used: anti-rabbit 1:5000 (Cat No. 205718, Abcam) and anti-rabbit 1:1000 (Cat No. 7074) and anti-mouse 1:1000 (Cat No. 7076), both from Cell Signaling Technology.

Second, the primary and secondary antibodies were validated using patient tissue lysates and above-mentioned TGX Stain-Free™ FastCast™ acrylamide kit (Bio-Rad, USA) (Suppl. Fig. S5a). A nonspecific 50 kDa band corresponding to endogenous IgG was detected upon staining with the Abcam secondary antibody (Suppl. Fig. S5b). Therefore, the CST secondary antibody was selected for further investigation (Suppl. Fig. S5b). Moreover, Parkin was not included in the analysis due to insufficient WB signal upon titration of the Millipore and CST primary antibodies (Suppl. Fig. S5c). Several groups have shown that BNIP3 dimer, as well as its monomer, can be detected by sodium dodecyl sulfate–polyacrylamide gel electrophoresis (SDS-PAGE) due to its high stability [33–35]. Indeed, in the current experiments, BNIP3 was well-stained as a dimer and monomer. BNIP3 dimerization is suggested to regulate mitophagy activation [36, 37]. Thus, based on densitometric analysis, one can assume that mitophagy is active.

Polypeptide separation revealed 15 kDa bands that were more prevalent in adjacent non-tumorous tissues than in tumorous tissues (Fig. 3). Mass-spectrometry (MS) analysis of these bands demonstrated the abundance of α, β, and δ hemoglobin subunits (Suppl. Table S2). The increased level of hemoglobin is compared with tumorous tissues could be associated with the abnormal and poorly organized cancer vasculature with altered permeability leading to a relatively poor blood supply of the latter [38, 39]. Therefore, the signal from this band was not considered for the densitometric analysis to minimize the contribution of different blood supplies in the tissues.

### WB image analysis

WB images were processed using Image Lab software (Bio-Rad). Densitometric analysis was performed using ImageJ 4.1 (LOCI, University of Wisconsin, Madison, WI, USA). The protein level of each clinical sample was normalized by the total protein level based on TGX stain-free gel (Bio-Rad). For further analysis of MQCP level alterations, the ${\log }_{2}({normalized\; protein\; level\; in\; tumorous\; tissue}/{normalized\; protein\; level\; in\; non}-{tumorous\; tissue})$ values were used.

### MS analysis

#### Protein identification from SDS-PAGE

In-gel digestion was carried out according to a protocol described by Shevchenko et al. [40].

#### Liquid chromatography–mass-spectrometry (LC-MS/MS) analysis

Peptides were separated by reverse-phase chromatography using an EASY nLC 1200 system (Thermo Scientific). Peptides were injected into a C18 pre-column (Acclaim PepMap 100, 75 µm × 2 cm, Thermo Scientific) and separated on a C18 reverse-phase LC column (PepMap RSLC C18, 2 µm, 100 A 75 µm × 25 cm, Thermo Scientific) at a flow rate of 300 nL/min using a linear gradient of buffer A (0.1% formic acid in water) and buffer B (0.1% formic acid in 80% acetonitrile) as follows: from 4% B to 30% B in 90 min; from 30% B to 100% B in 25 min; and 100% B for 5 min. Automated online analyses were conducted in positive mode by a Q Exactive HF hybrid Quadrupole Orbitrap mass spectrometer (Thermo Scientific) equipped with a nano-electrospray source and Xcalibur software (v.4.1, Thermo Scientific). Each MS scan was obtained at a resolution of 120,000 at the 380–1400 *m*/*z* range. The top 15 most intense multiple charged ions were selected with an isolation window of 1.2 *m*/*z* and subjected to MS/MS. The maximum ion injection time was set to 50 ms for MS and 80 ms for MS/MS scans. The automatic gain control target value was 3 × 10^6^ for MS scans and 1 × 10^5^ for MS/MS scans.

### Database search

Raw files were analyzed using Sequest HT in Proteome Discoverer (Thermo Fisher Scientific, CS version 2.5) against the Uniprot Human 9606 database (March 2022). The following search parameters were used: trypsin as a digestion enzyme; maximum number of missed cleavages 2; a peptide mass tolerance of 15 ppm, a fragment mass tolerance of 0.6 Da; carbamidomethyl as a fixed modification on cysteine; and methionine oxidation as variable modification.

The identified proteins were validated using SCAFFOLD software (Version 5.0.0; Proteome Software Inc., Portland, OR, USA). Identifications were based on a minimum 80% peptide identification probability (using the Scaffold Local FDR algorithm), and a minimum 95% protein identification probability using the Protein Prophet algorithm [41]. Proteins that contained similar peptides and that could not be differentiated based on LC-MS/MS analysis alone were grouped to satisfy the principles of parsimony. Label-free quantitative analysis was done using the total number of spectral counts; normalization was performed to account for variations between samples. Quantitative differences were statistically analyzed by Student’s t-test and differences with *p* ≤ 0.05 were considered significant.

### Establishing an MQCP risk score

Univariate and multivariate Cox proportional hazards models were used to analyze the impact of MQCP alterations on patient survival. HR and 95% confidence intervals (CI) were calculated. The time independence of variables was verified by the Schoenfeld test. To test influential observations or outliers, the deviance residual plot was analyzed. To determine the final multivariate Cox model, the bidirectional stepwise regression was used. Next, the regression coefficient obtained by multivariate Cox regression analysis was multiplied by the ${\log }_{2}({normalized\; protein\; level\; in\; tumorous\; tissue}/{normalized\; protein\; level\; in\; non}-{tumorous\; tissue})$ value of each protein to construct the MQCP-based prognostic signature. The prognostic MQCP risk score of each patient was determined as: MQCP risk score = $\mathop{\sum }\nolimits_{i=1}^{n}{regression\; coefficient\; protein\; i}* {\log }_{2}({normalized\; protein\; i\; level\; in\; tumorous\; tissue}/{normalized\; protein\; i\; level\; in\; non}-{tumorous\; tissue}).\,$Patients were divided into low- and high-risk groups based on the median MQCP risk score as the cut-off. The survival differences between the groups were evaluated by the Kaplan–Meier method and compared using log-rank statistical methods. Univariate and multivariate Cox regression analyses were performed to identify independent prognostic parameters and to validate the independent prognostic value of the MQCP risk score and clinicopathological parameters.

### Construction and validation of a predictive nomogram

Based on the prognostic factors determined by the multivariate Cox proportional hazards regression analysis, a nomogram was formulated to predict the 1-, 3-, and 5-year OS of patients. C-index was calculated to assess the predictive accuracy of the model by a bootstrap method and to compare with the American Joint Committee on Cancer (AJCC) TNM staging system. A calibration plot was used to compare the predicted probability with the observed one at a specific time point. If the model is ideal, pairs of observed and the predicted probabilities lie on the diagonal line.

### Statistical analysis

All continuous data were tested for homogeneity of variance and normality using Levene’s test and the Shapiro–Wilk test, respectively. Two or three groups were compared using the two-sample Wilcoxon test or the Kruskal–Wallis tests, respectively. A one-sample Wilcoxon test was applied to estimate the significant alteration in the tumorous tissue and adjacent non-tumorous tissue. Spearman rank correlation coefficients were calculated to analyze the correlations among MQCP level alterations. Survival curves were compared with the log-rank test. Statistical analyses were performed, and the graphics were generated using R (4.1.2). In the analyses, *p* ≤ 0.05 were considered significant.

### Supplementary information

Supplementary Files

## Data Availability

All data are available in the main text or the supplementary materials.
