# Supplementary material for: Prognostic signature based on mitochondria quality control proteins for the prediction of lung adenocarcinoma patients survival
Source: Cell Death Discov. 2023 Sep 25;9:352. doi: 10.1038/s41420-023-01649-x (PMC10519931; doi:10.1038/s41420-023-01649-x)
Supplement: Supplementary file 1 — Supplementary Files [file 41420_2023_1649_MOESM1_ESM.pdf]

Figure S1. WB of analyzed proteins in tumorous (T) and adjacent non-tumorous tissues (N) of 80 patients with LUAD.

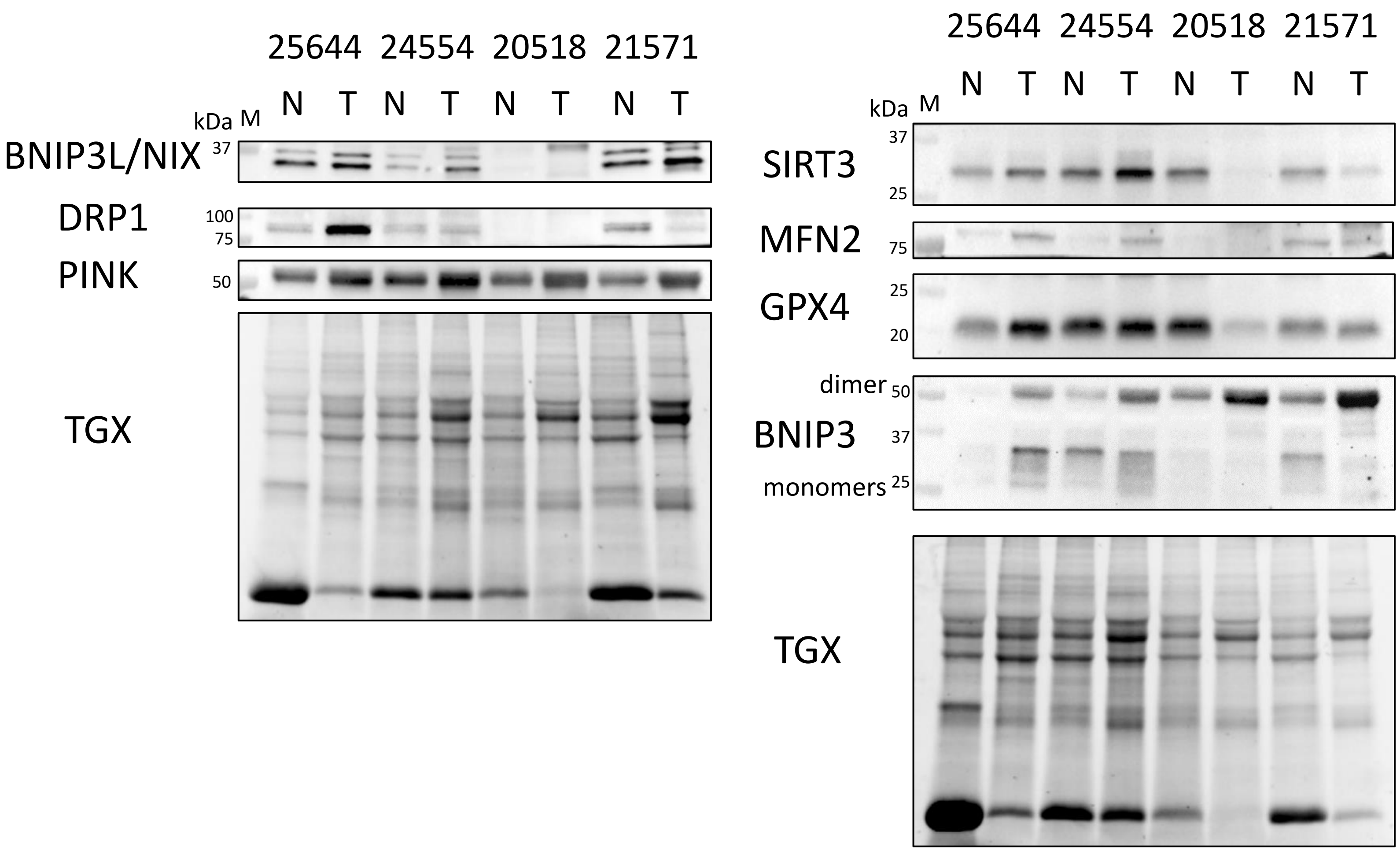

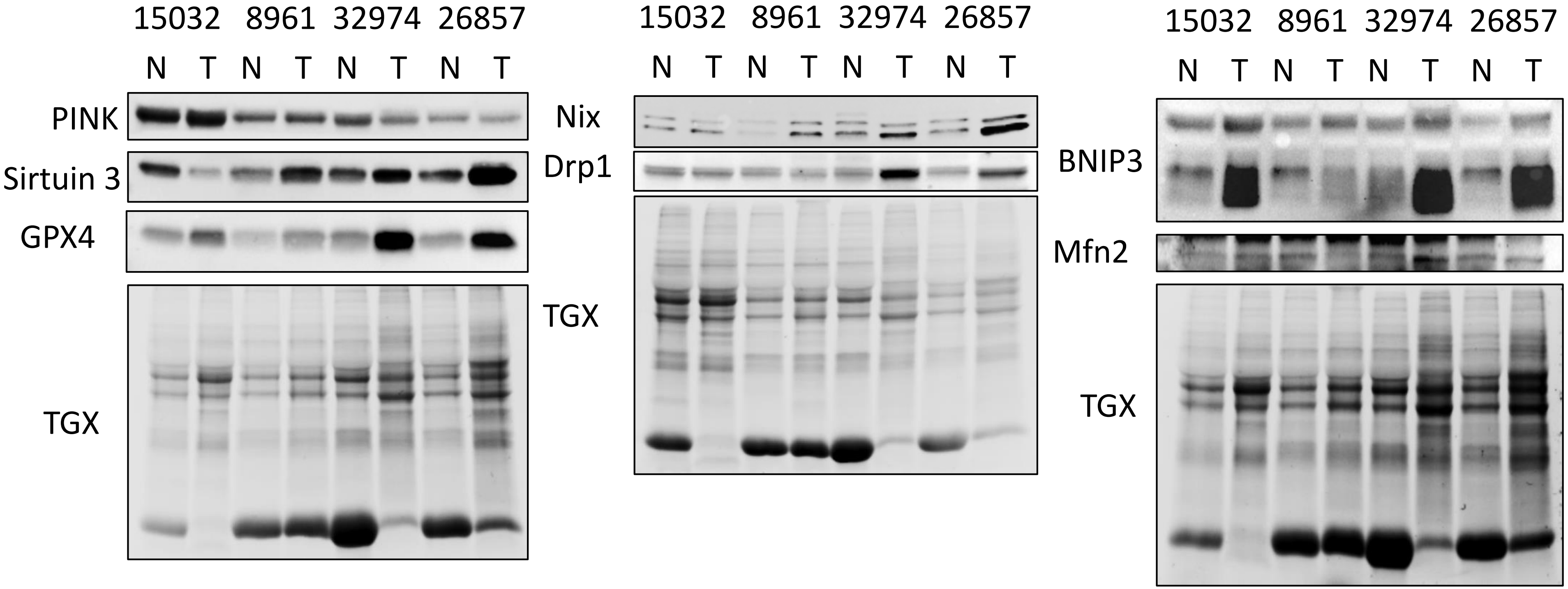
15032
8961
32974
26857
N
T
N
T
N
T
N
T
PINK
Sirtuin 3
GPX4
TGX
Nix
Drp1
TGX
BNIP3
Mfn2
TGX

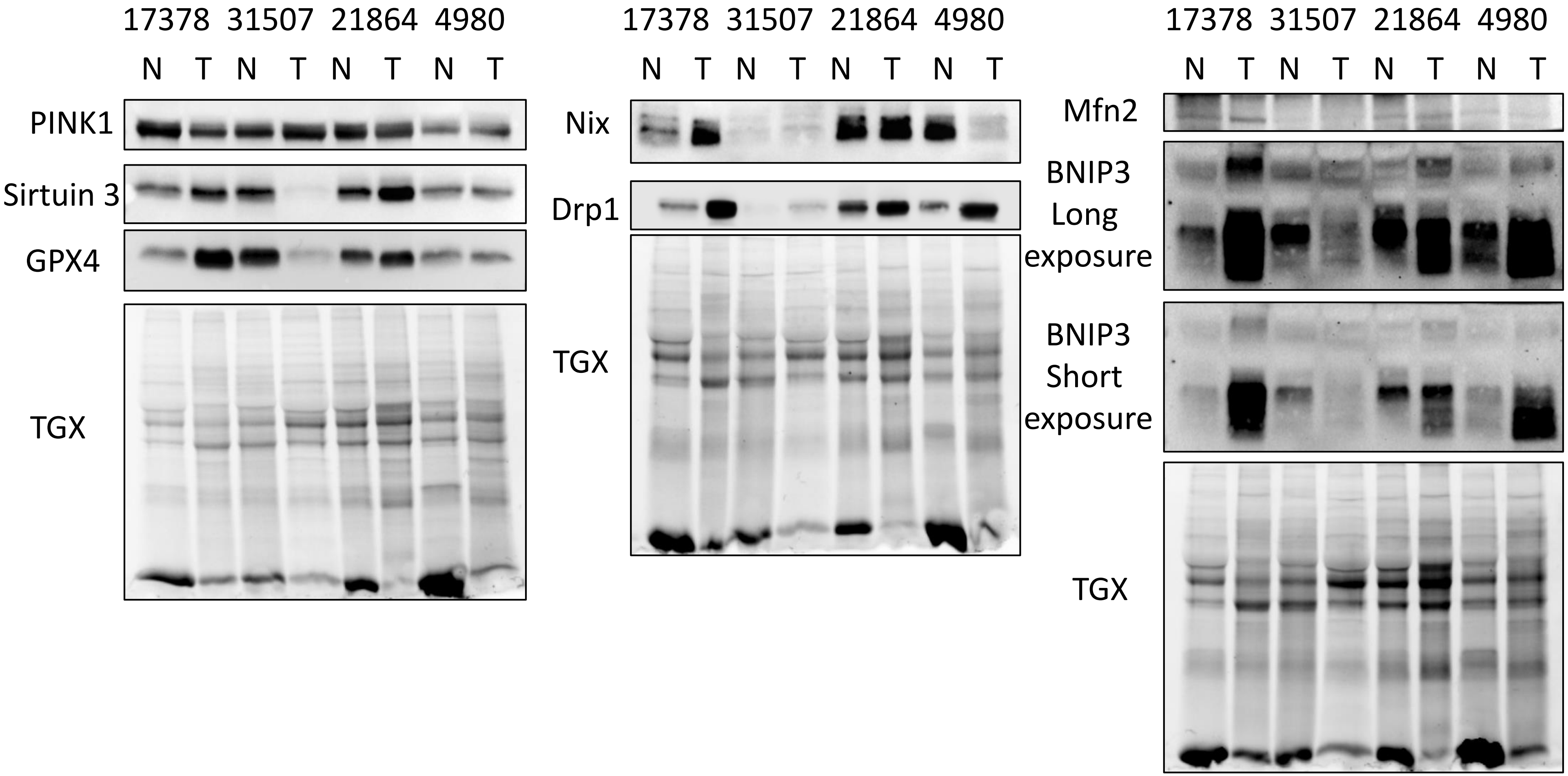
17378 31507 21864 4980
N T N T N T N T
PINK1
Sirtuin 3
GPX4
TGX
17378 31507 21864 4980
N T N T N T N T
Nix
Drp1
TGX
17378 31507 21864 4980
N T N T N T N T
Mfn2
BNIP3 Long exposure
BNIP3 Short exposure
TGX

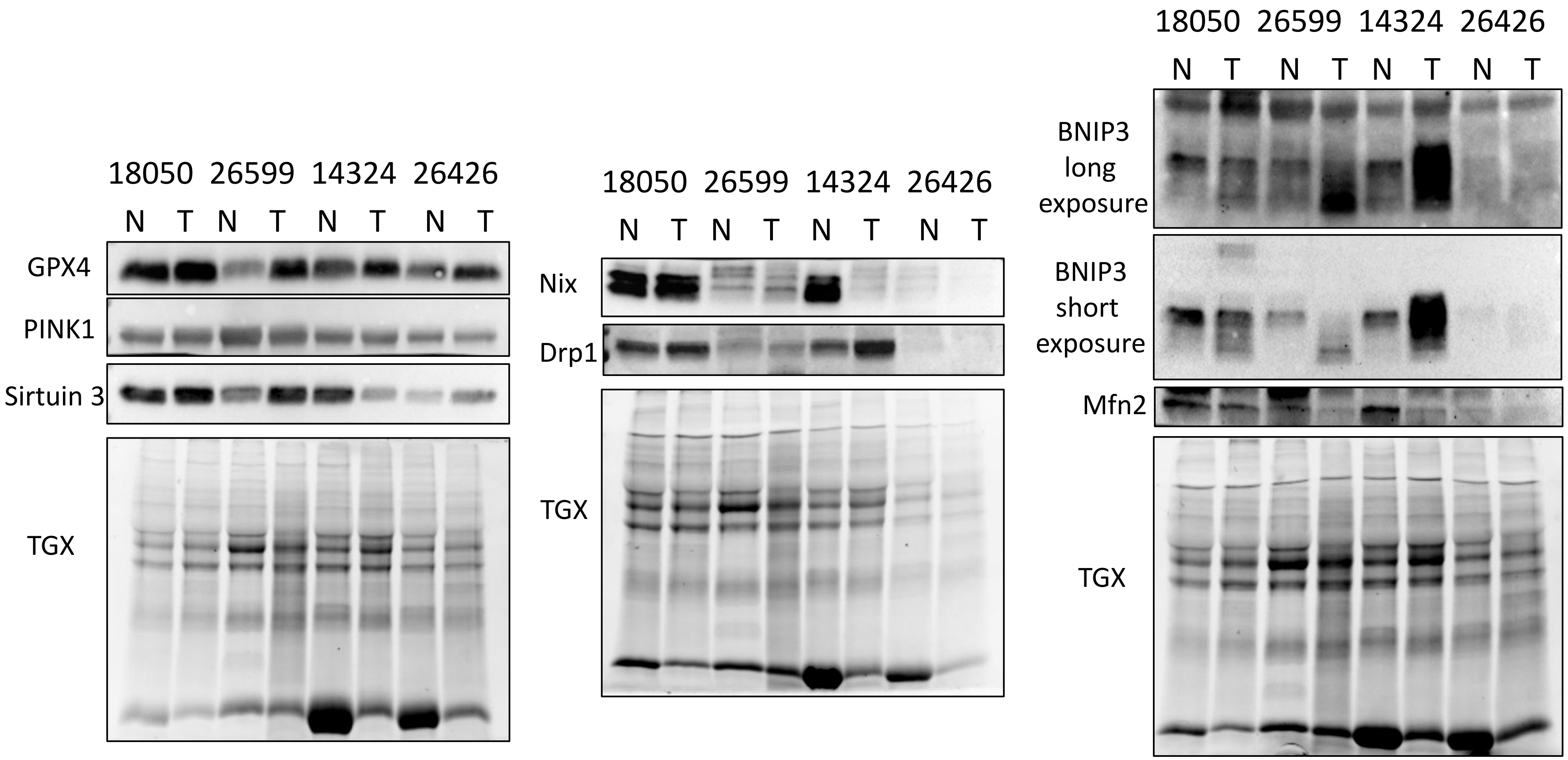
18050 26599 14324 26426
N T N T N T N T
GPX4
PINK1
Sirtuin 3
TGX
18050 26599 14324 26426
N T N T N T N T
Nix
Drp1
TGX
18050 26599 14324 26426
N T N T N T N T
BNIP3 long exposure
BNIP3 short exposure
Mfn2
TGX

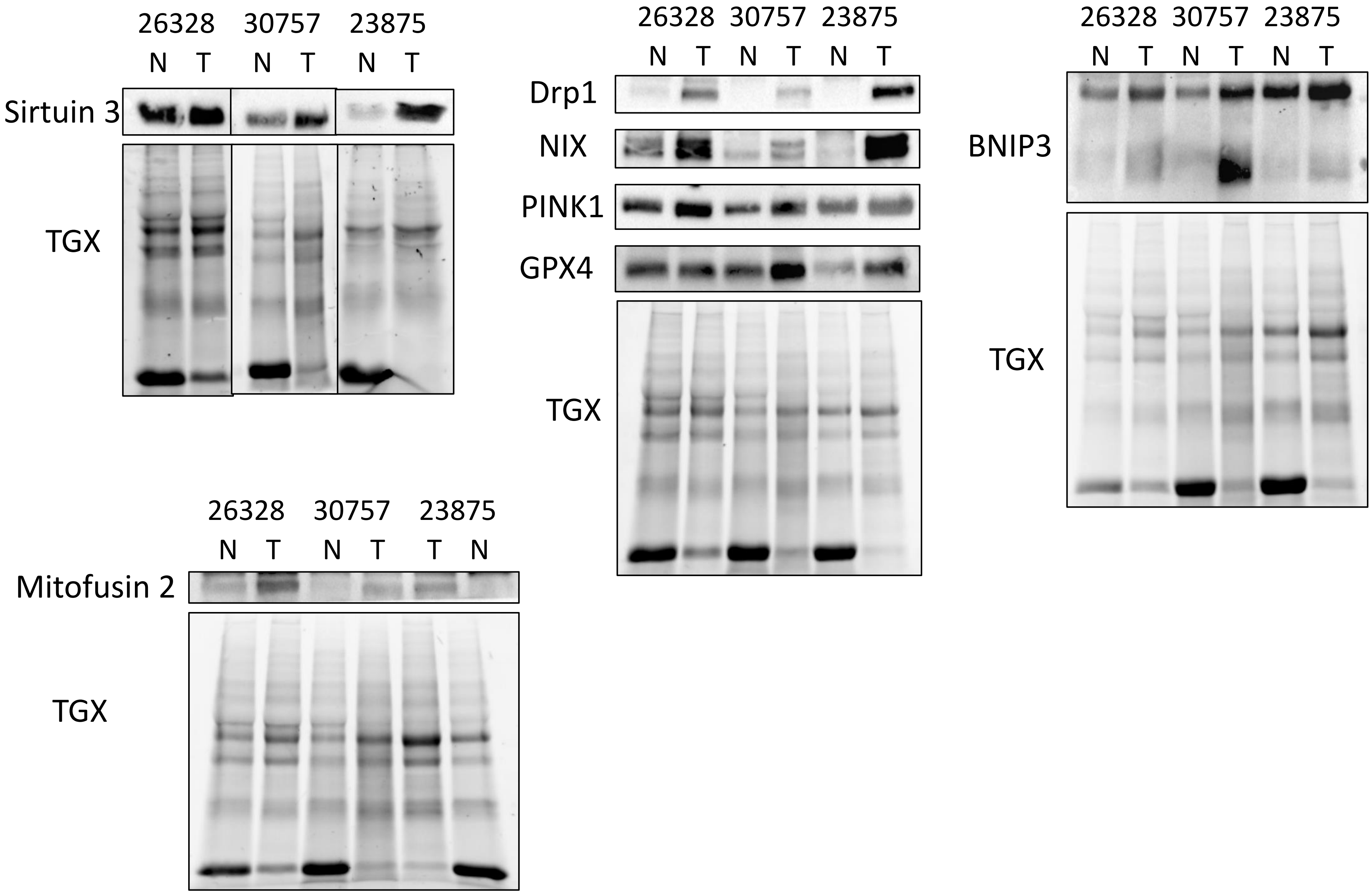
26328 30757 23875
N T N T N T
Sirtuin 3
TGX
26328 30757 23875
N T N T N T
Drp1
NIX
PINK1
GPX4
TGX
26328 30757 23875
N T N T N T
BNIP3
TGX
26328 30757 23875
N T N T T N
Mitofusin 2
TGX

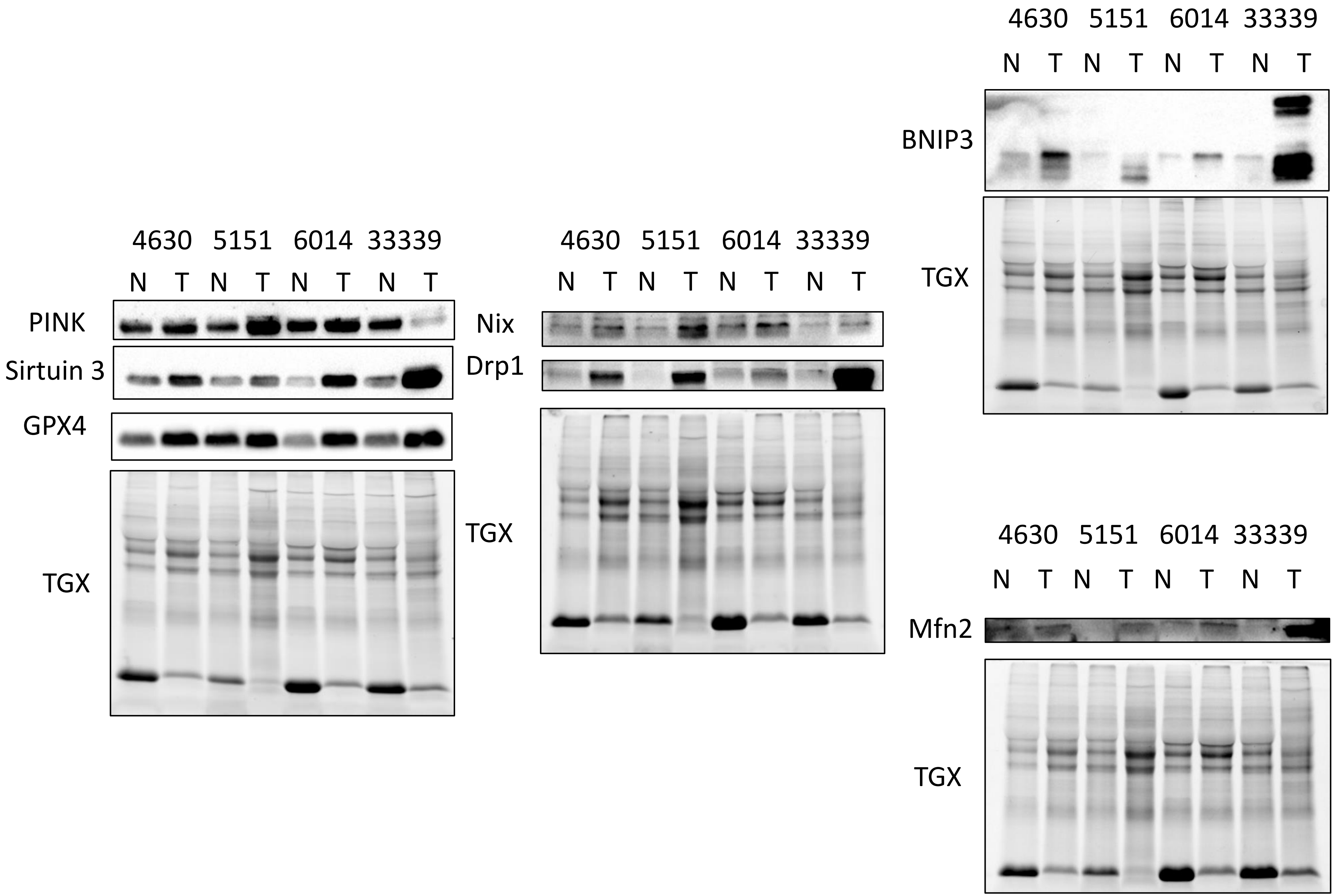
4630 5151 6014 33339
N T N T N T N T
PINK
Sirtuin 3
GPX4
TGX
4630 5151 6014 33339
N T N T N T N T
Nix
Drp1
TGX
4630 5151 6014 33339
N T N T N T N T
BNIP3
TGX
4630 5151 6014 33339
N T N T N T N T
Mfn2
TGX

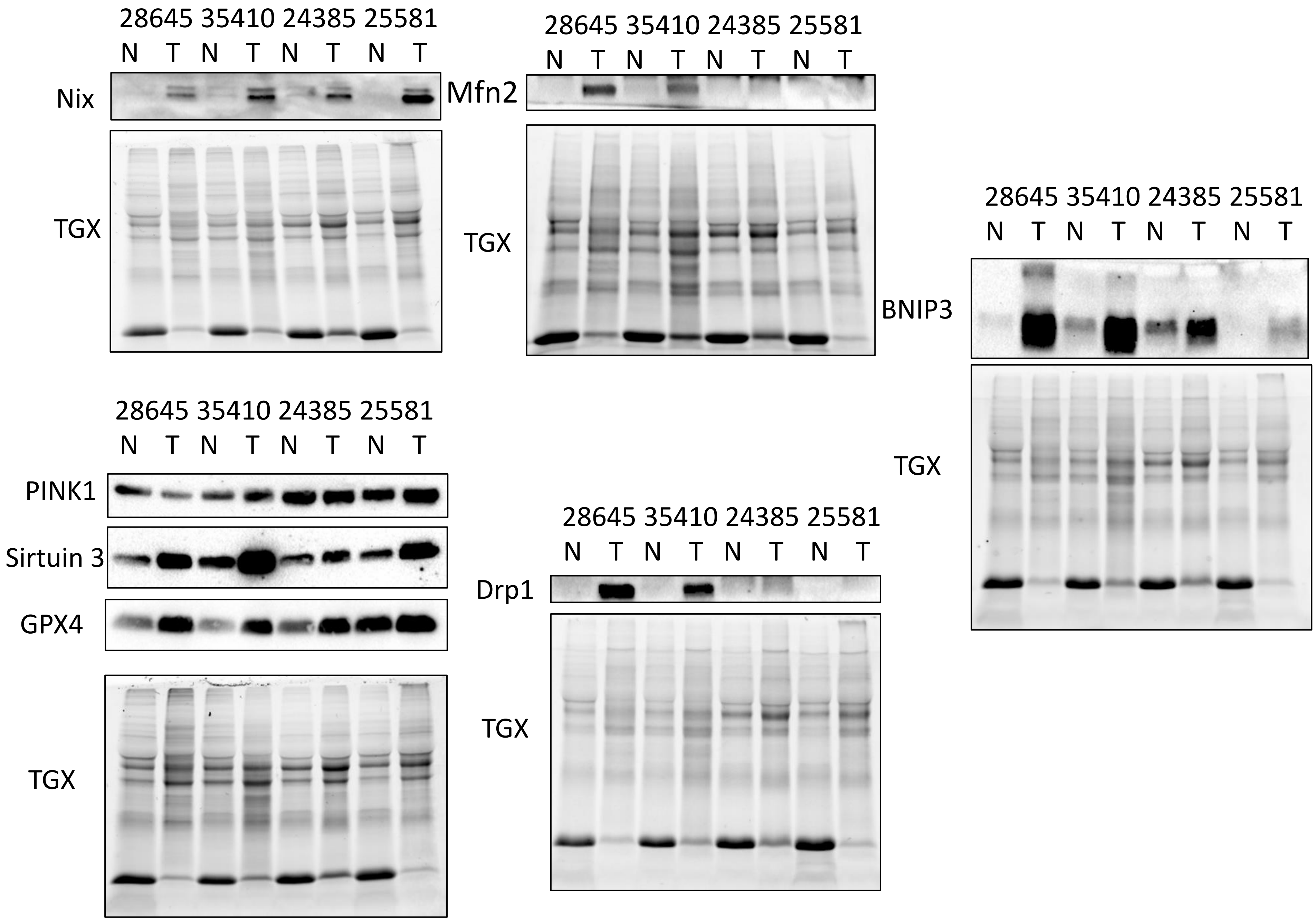
28645 35410 24385 25581
N T N T N T N T
Nix
TGX
28645 35410 24385 25581
N T N T N T N T
Mfn2
TGX
28645 35410 24385 25581
N T N T N T N T
BNIP3
TGX
28645 35410 24385 25581
N T N T N T N T
PINK1
Sirtuin 3
GPX4
TGX
28645 35410 24385 25581
N T N T N T N T
Drp1
TGX

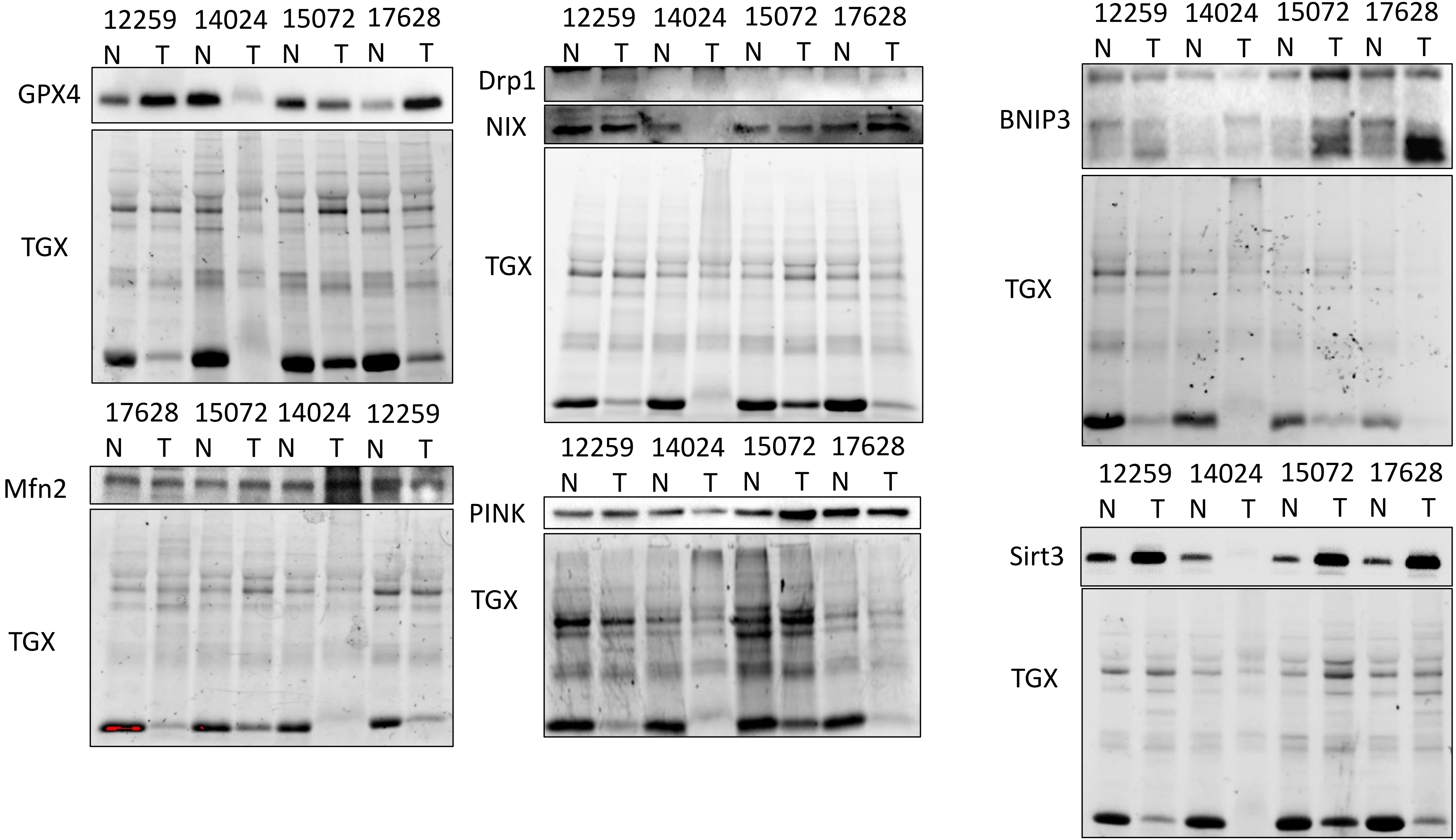
12259 14024 15072 17628
N T N T N T N T
GPX4
TGX
17628 15072 14024 12259
N T N T N T N T
Mfn2
TGX
12259 14024 15072 17628
N T N T N T N T
Drp1
NIX
TGX
12259 14024 15072 17628
N T N T N T N T
PINK
TGX
12259 14024 15072 17628
N T N T N T N T
BNIP3
TGX
12259 14024 15072 17628
N T N T N T N T
Sirt3
TGX

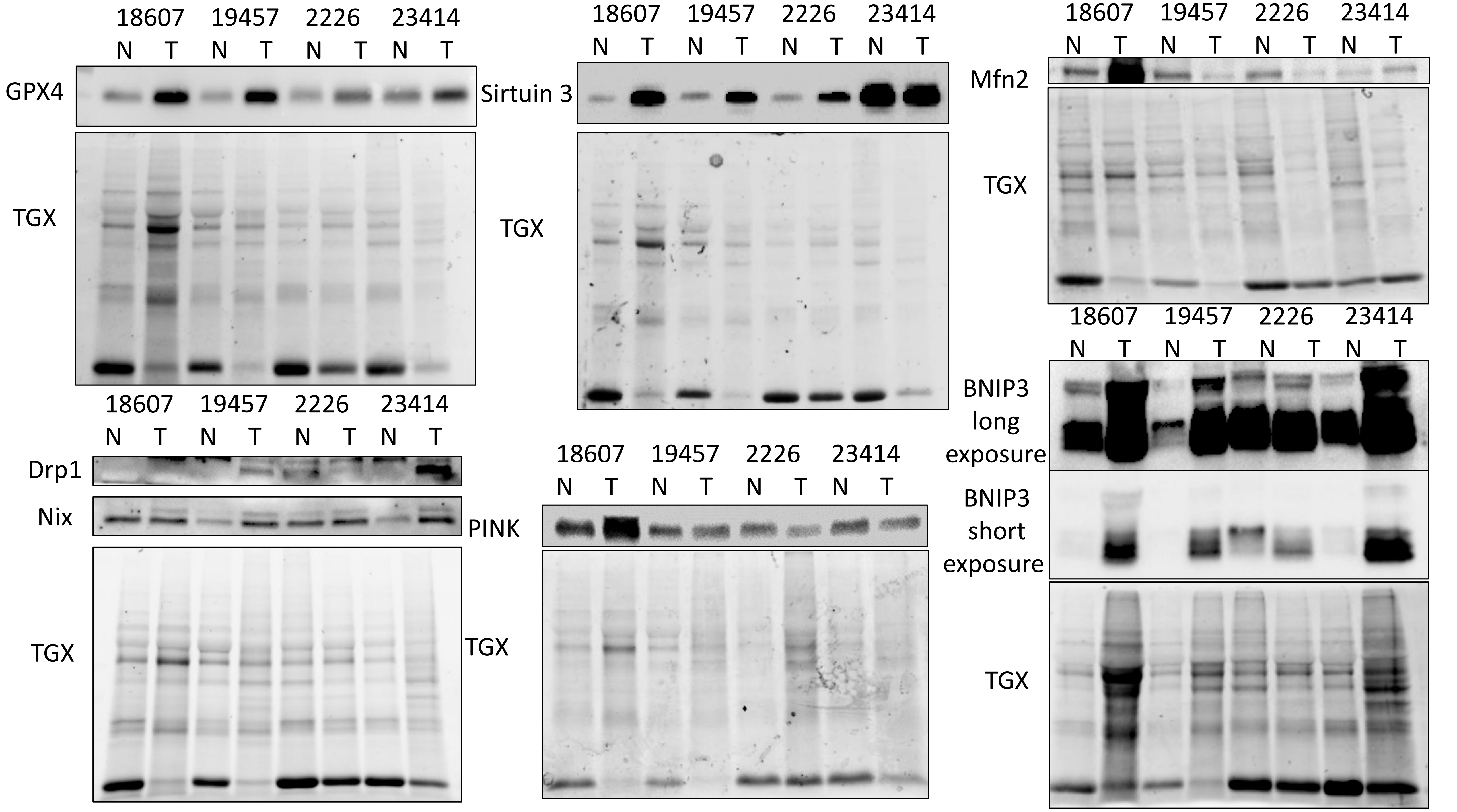
18607 19457 2226 23414
N T N T N T N T
GPX4
TGX
Sirtuin 3
TGX
Mfn2
TGX
BNIP3 long exposure
BNIP3 short exposure
TGX
Drp1
Nix
TGX
PINK
TGX

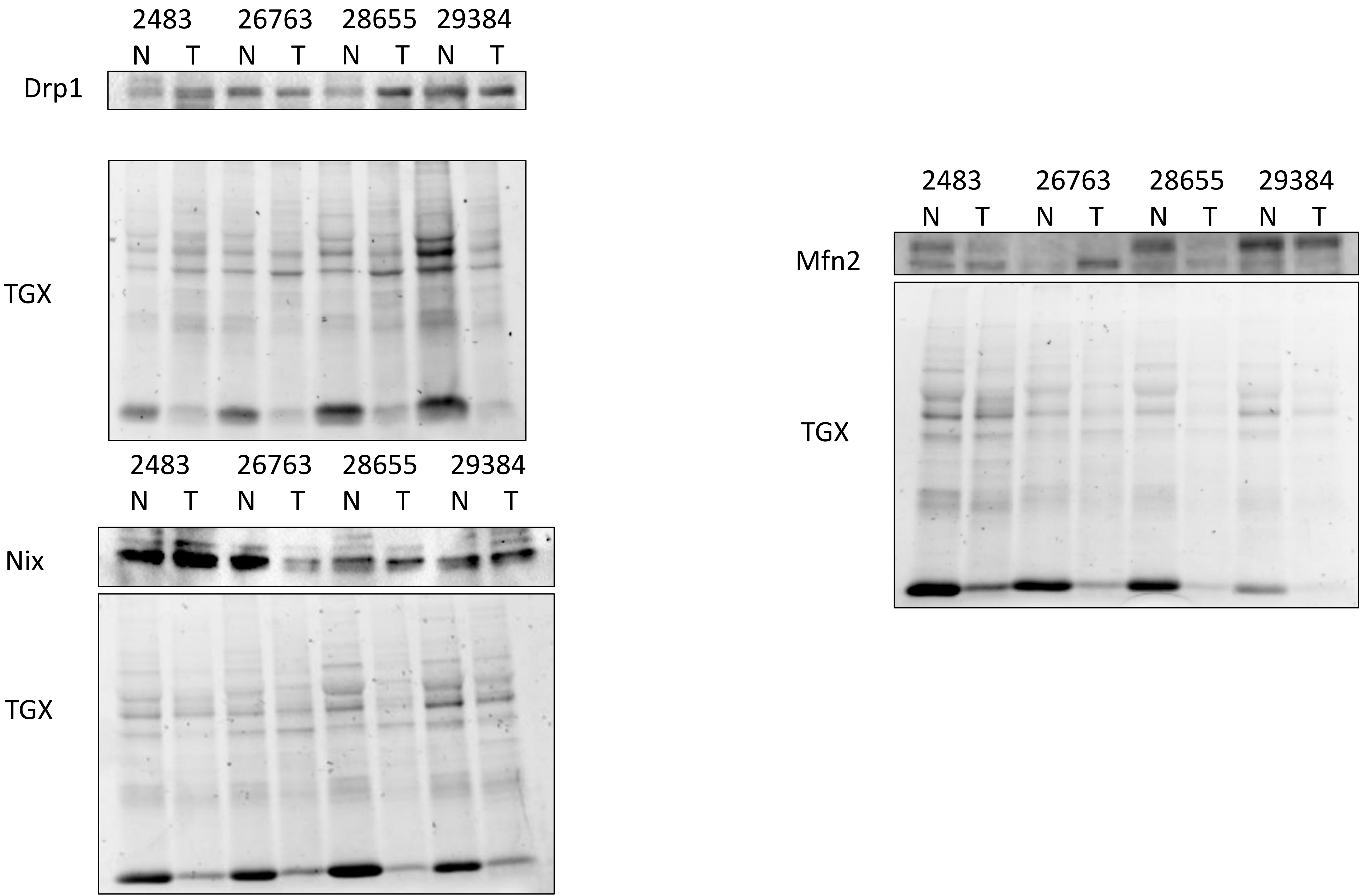
2483 26763 28655 29384
N T N T N T N T
Drp1
TGX
2483 26763 28655 29384
N T N T N T N T
Nix
TGX
2483 26763 28655 29384
N T N T N T N T
Mfn2
TGX

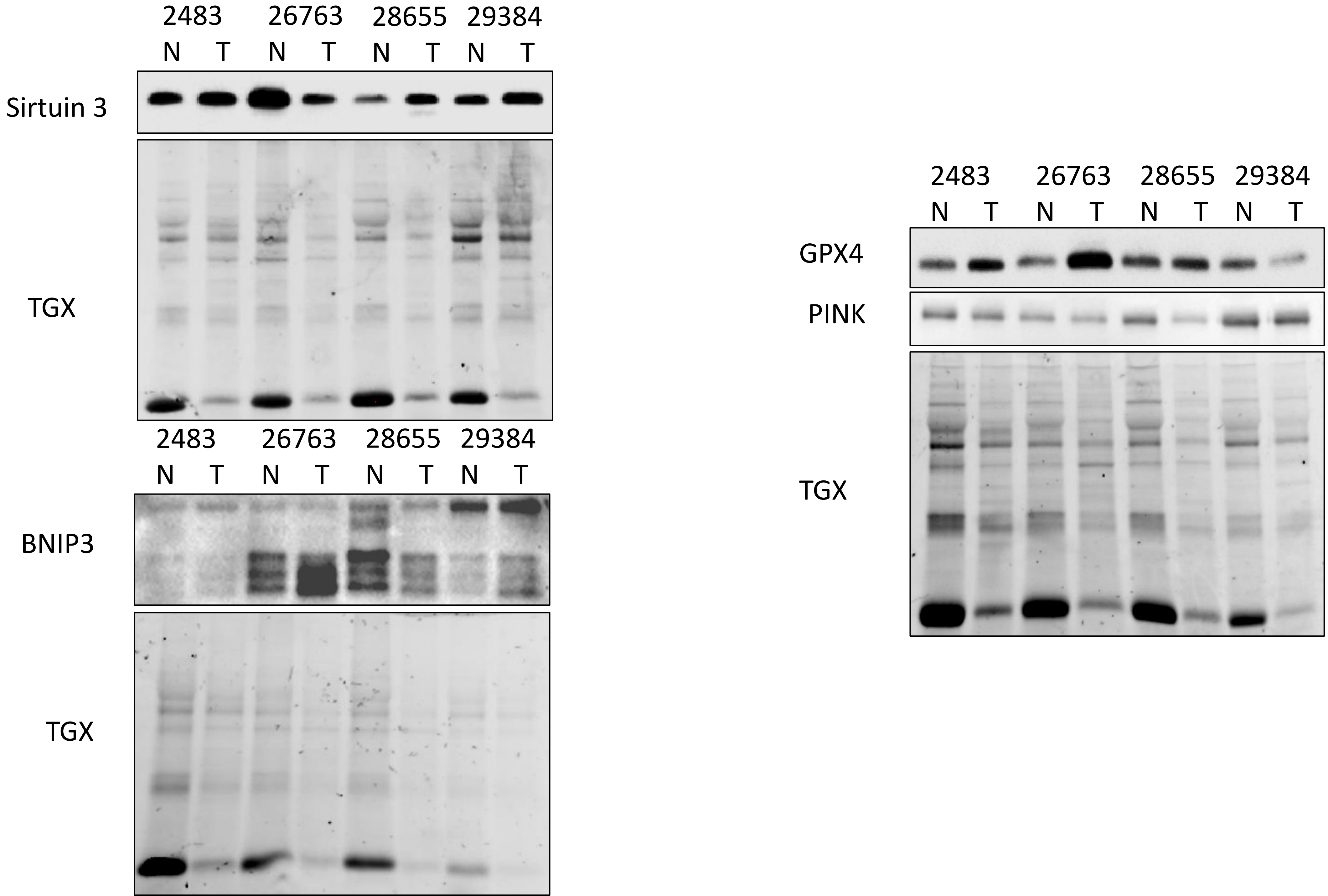
2483
26763
28655
29384
N T N T N T N T
Sirtuin 3
TGX
2483
26763
28655
29384
N T N T N T N T
BNIP3
TGX
2483
26763
28655
29384
N T N T N T N T
GPX4
PINK
TGX

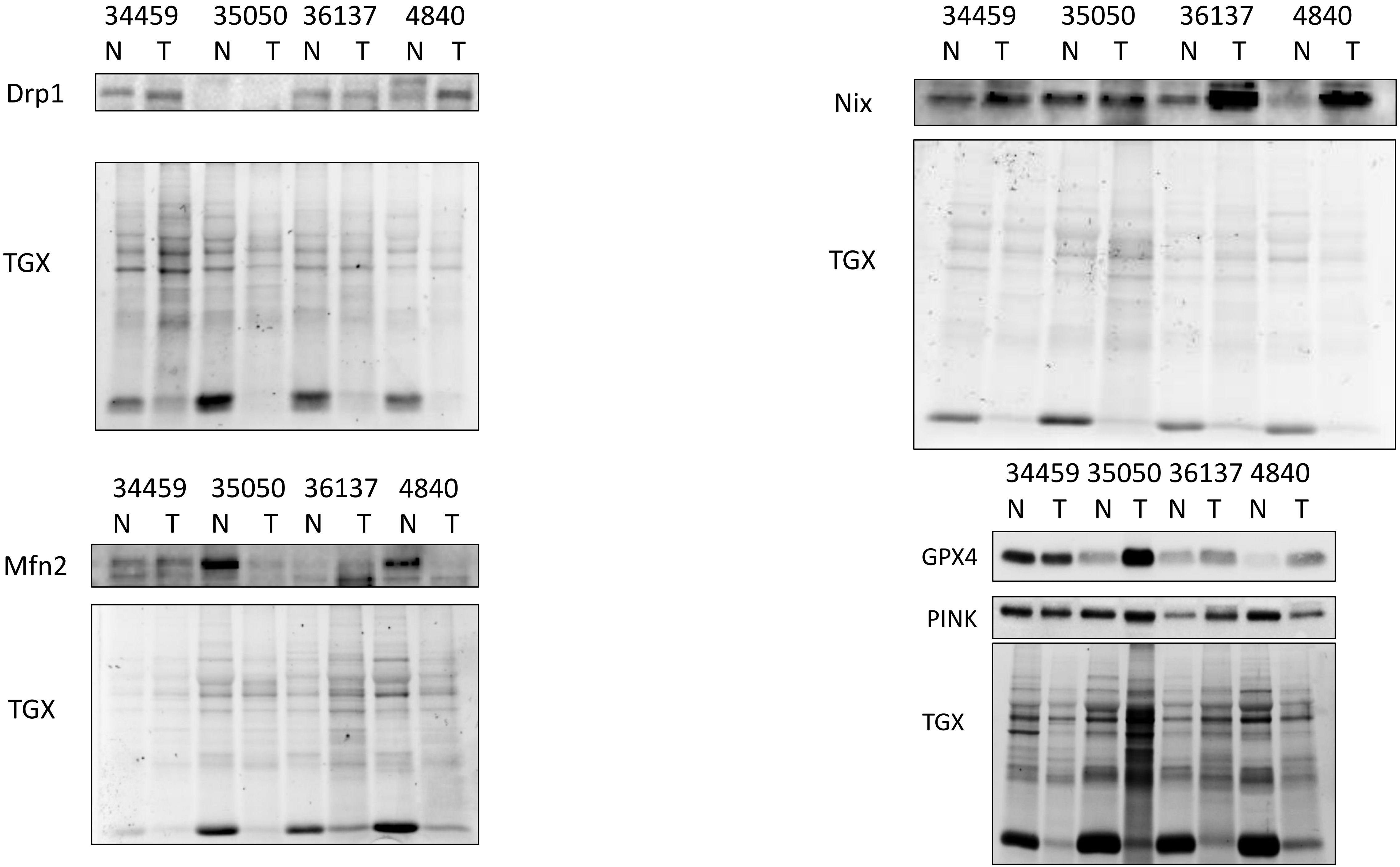
34459 35050 36137 4840
N T N T N T N T
Drp1
TGX
34459 35050 36137 4840
N T N T N T N T
Mfn2
TGX
34459 35050 36137 4840
N T N T N T N T
Nix
TGX
34459 35050 36137 4840
N T N T N T N T
GPX4
PINK
TGX

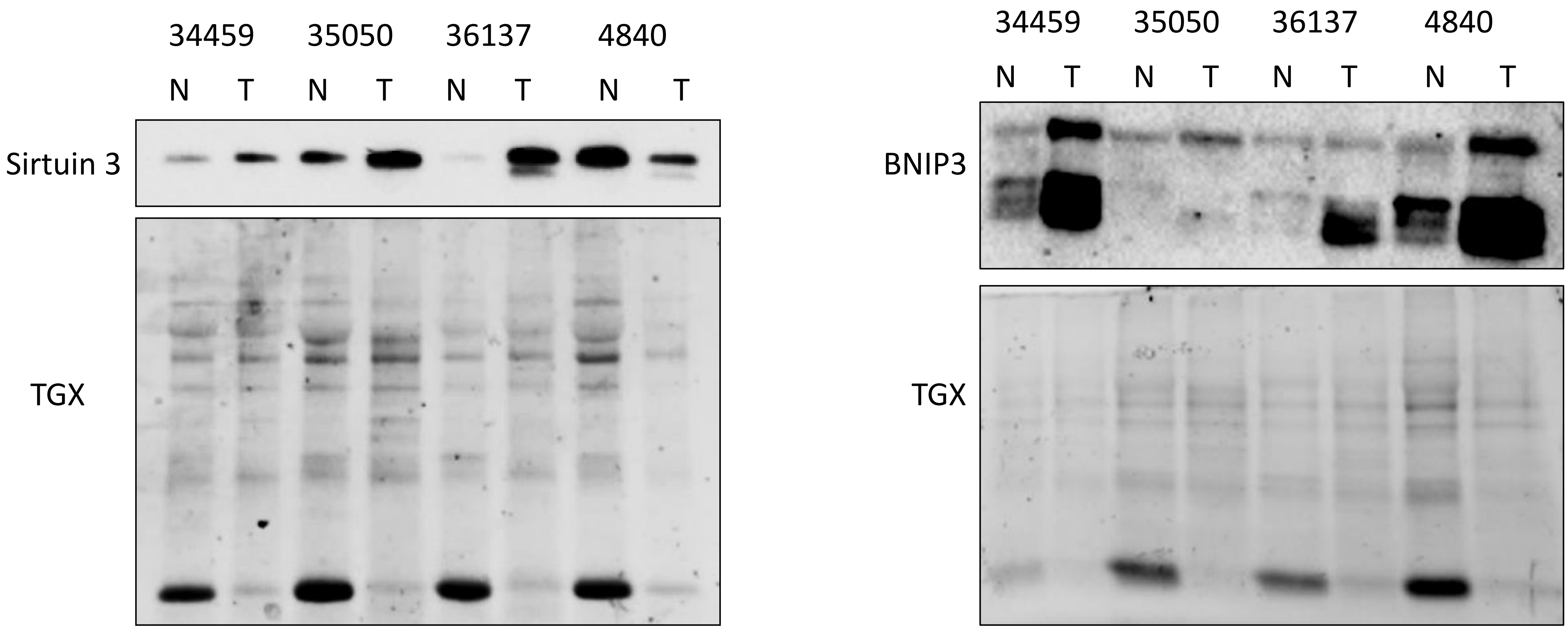
34459 35050 36137 4840
N T N T N T N T
Sirtuin 3
TGX
34459 35050 36137 4840
N T N T N T N T
BNIP3
TGX

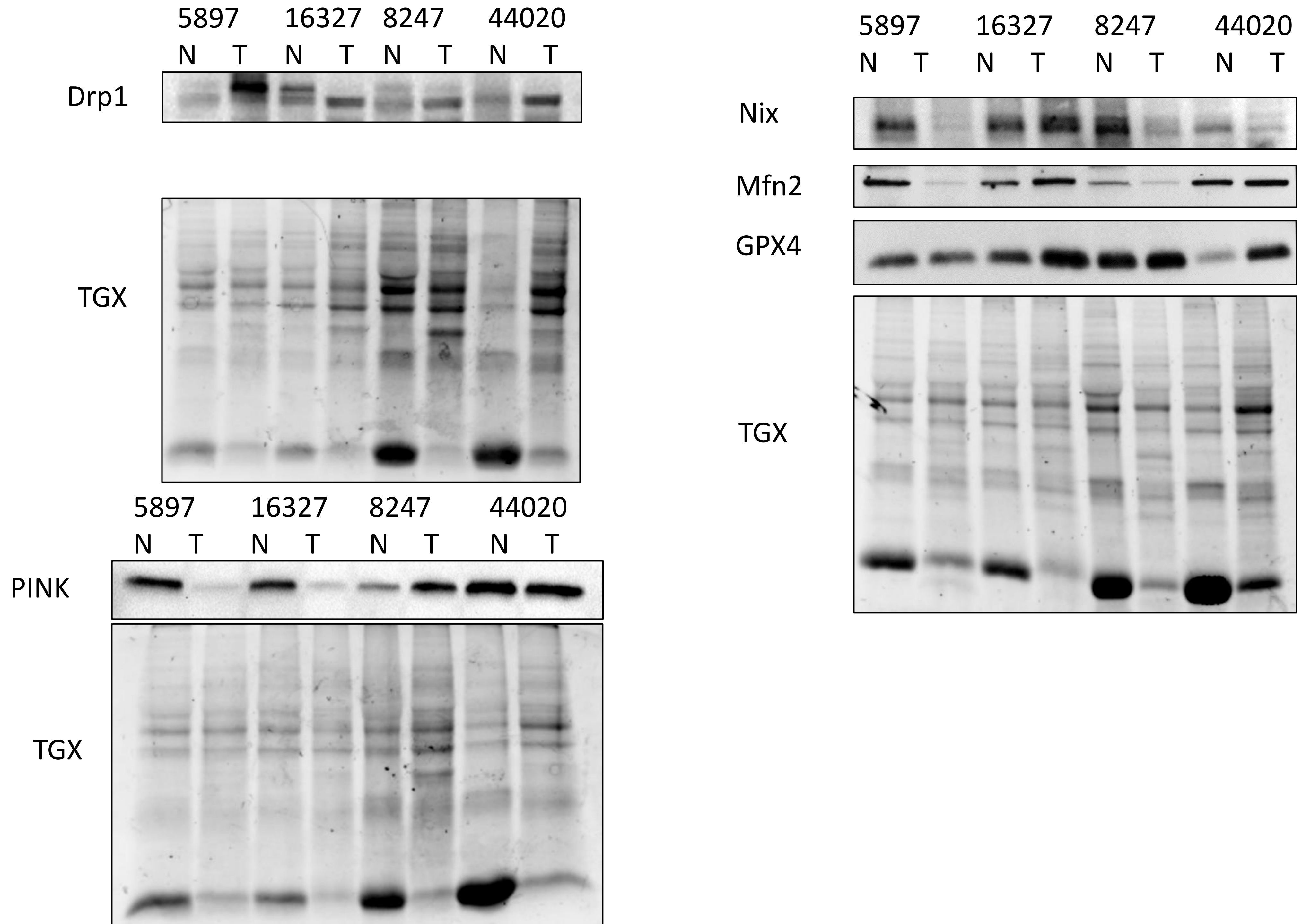
5897
16327
8247
44020
N T N T N T N T
Drp1
TGX
5897
16327
8247
44020
N T N T N T N T
PINK
TGX
5897
16327
8247
44020
N T N T N T N T
Nix
Mfn2
GPX4
TGX

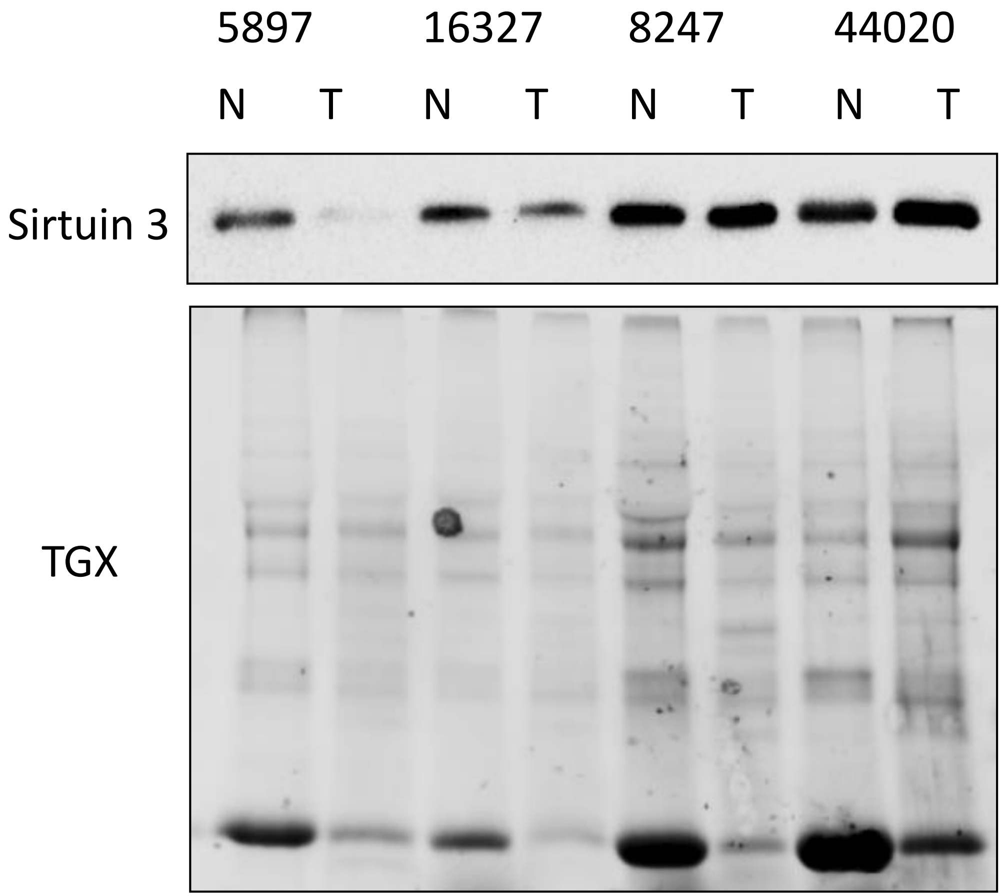
5897
16327
8247
44020
N T N T N T N T
Sirtuin 3
TGX

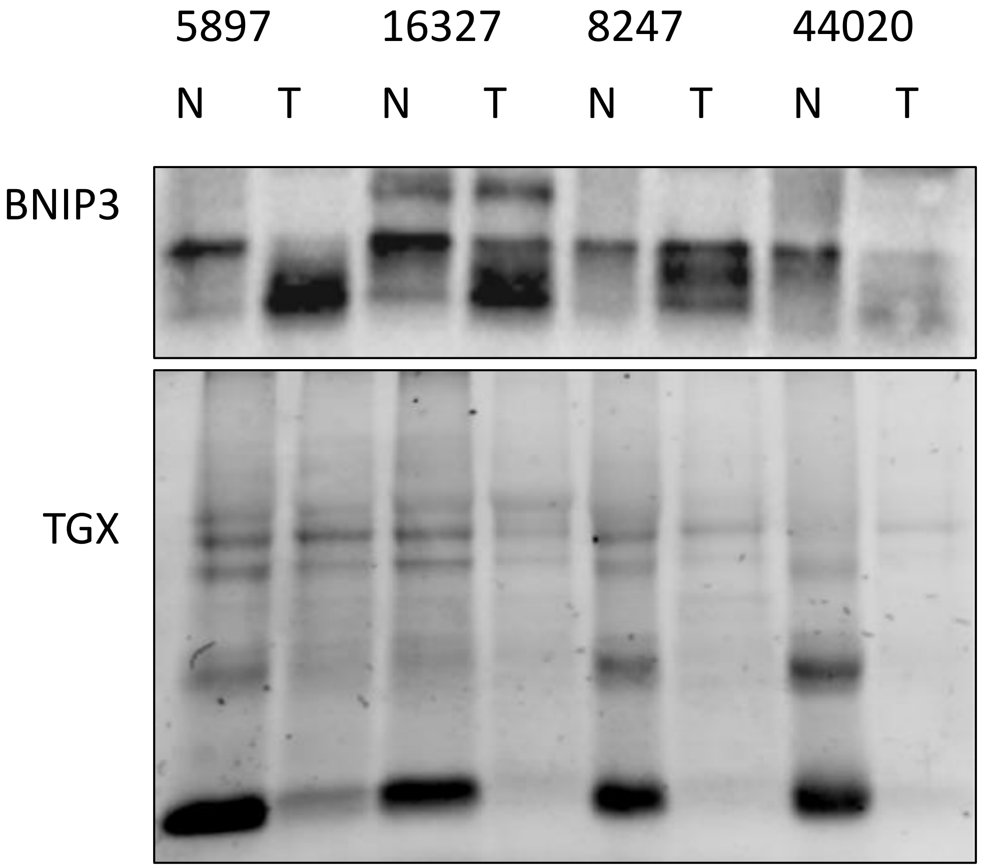
5897
16327
8247
44020
N T N T N T N T
BNIP3
TGX

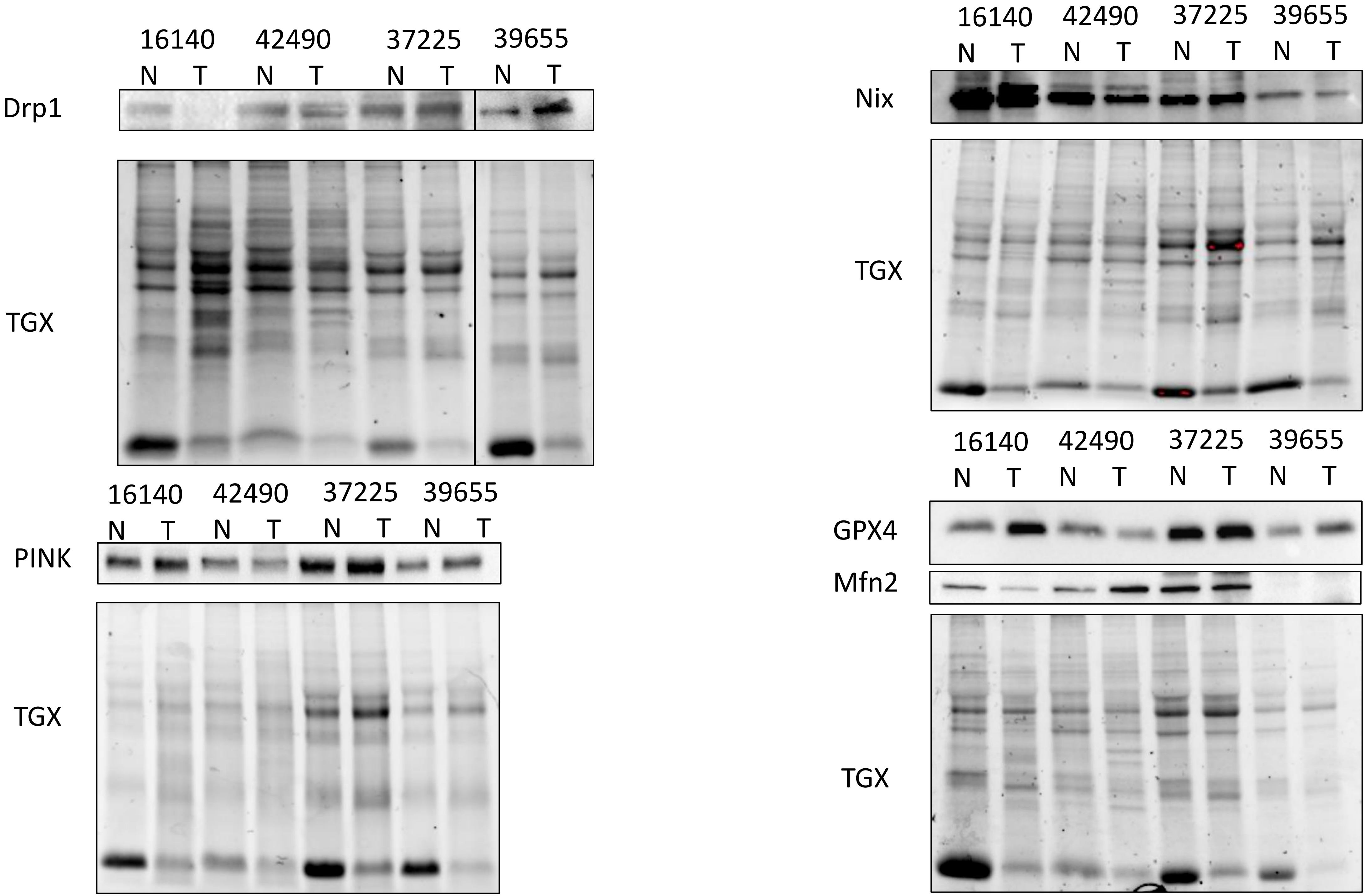

16140 42490 37225 39655
N T N T N T N T
Drp1
TGX
16140 42490 37225 39655
N T N T N T N T
PINK
TGX
16140 42490 37225 39655
N T N T N T N T
Nix
TGX
16140 42490 37225 39655
N T N T N T N T
GPX4
Mfn2
TGX

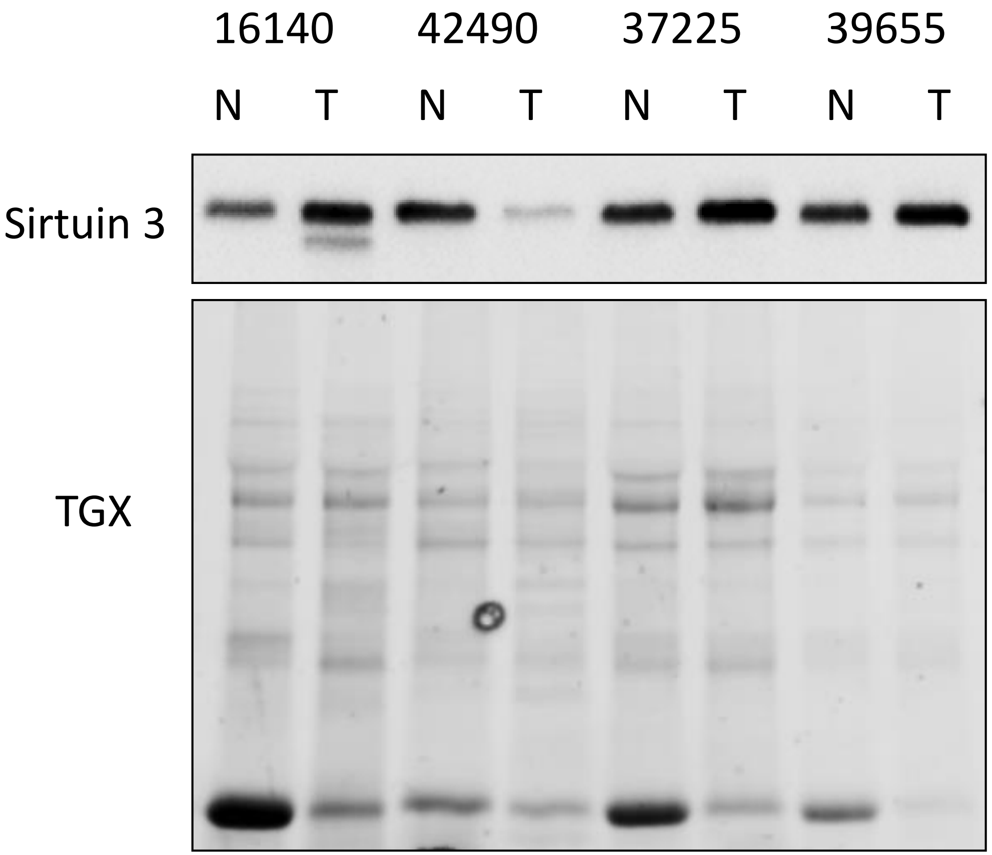
16140 42490 37225 39655
N T N T N T N T
Sirtuin 3
TGX

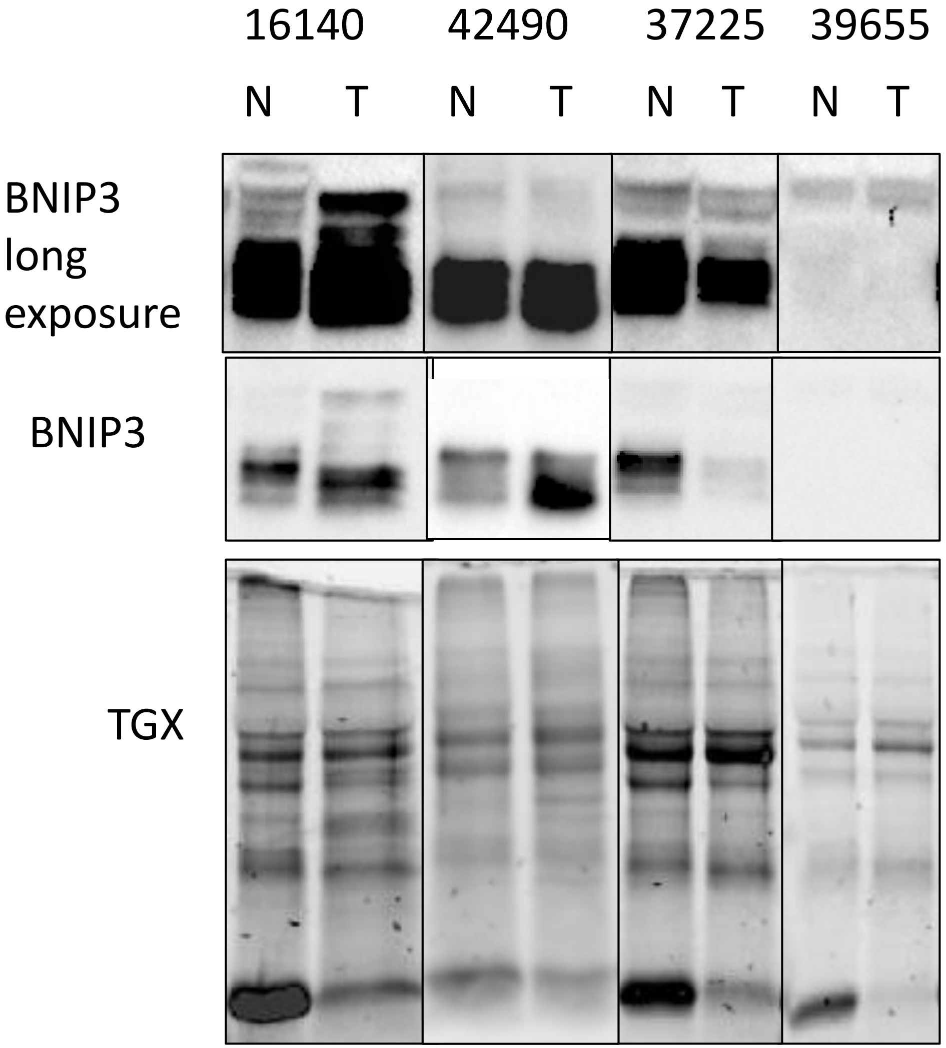
16140 42490 37225 39655
N T N T N T N T
BNIP3 long exposure
BNIP3
TGX

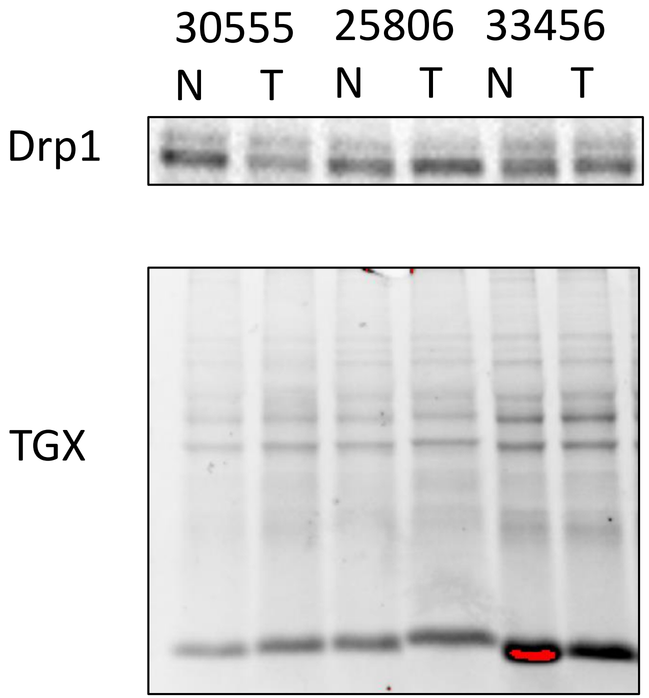
30555 25806 33456
N T N T N T
Drp1
TGX

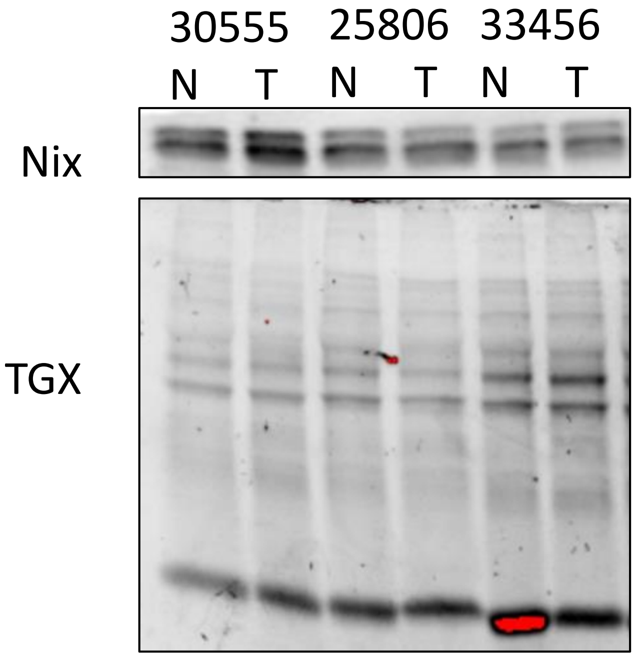
30555 25806 33456
N T N T N T
Nix
TGX

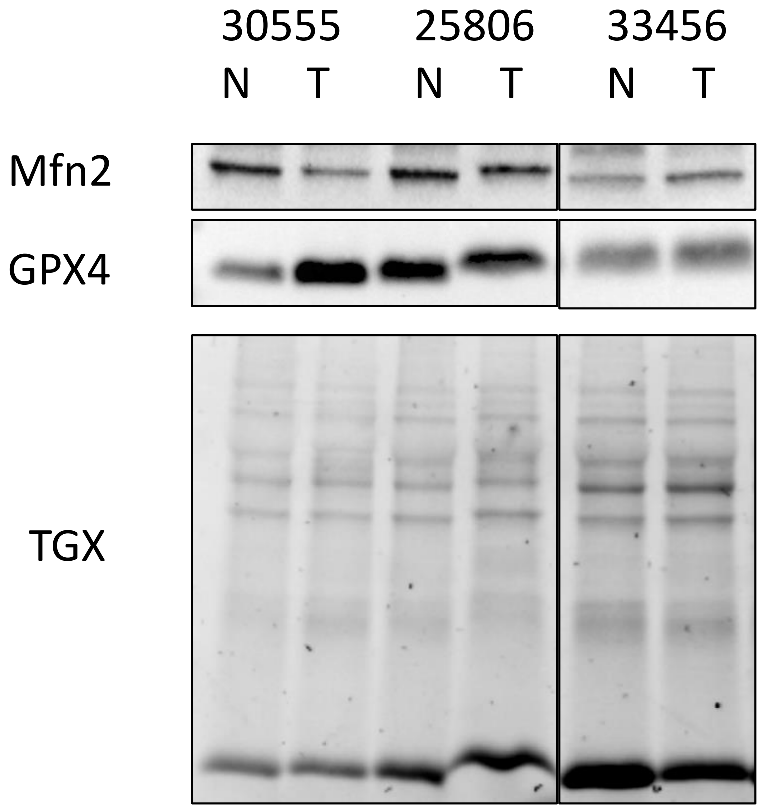
30555 25806 33456
N T N T N T
Mfn2
GPX4
TGX

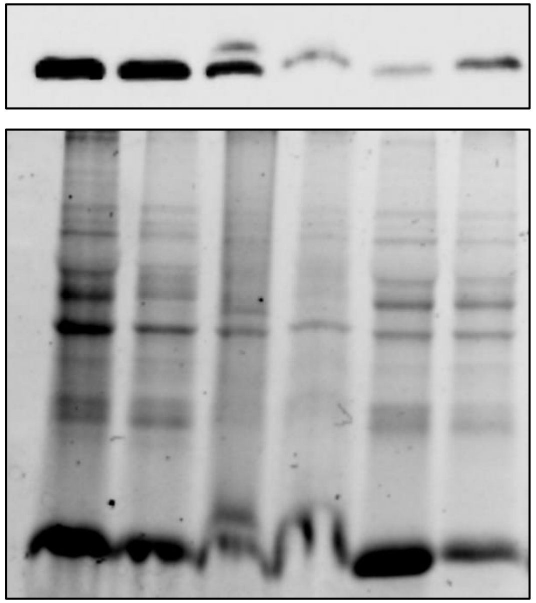
30555 25806 33456
N T N T N T
Sirtuin 3
TGX

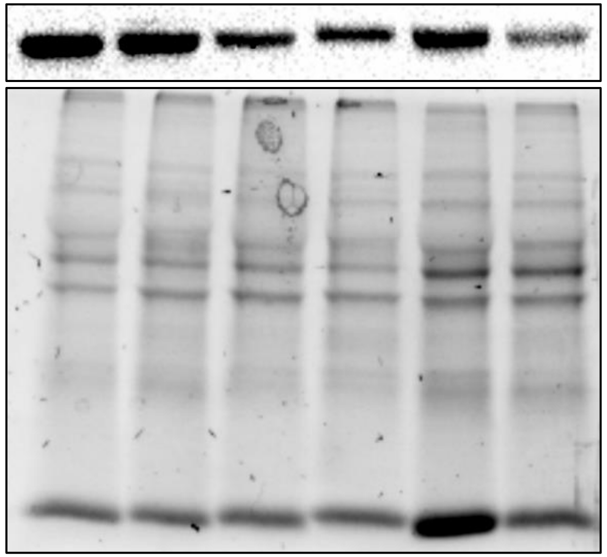
30555 25806 33456
N T N T N T
PINK
TGX

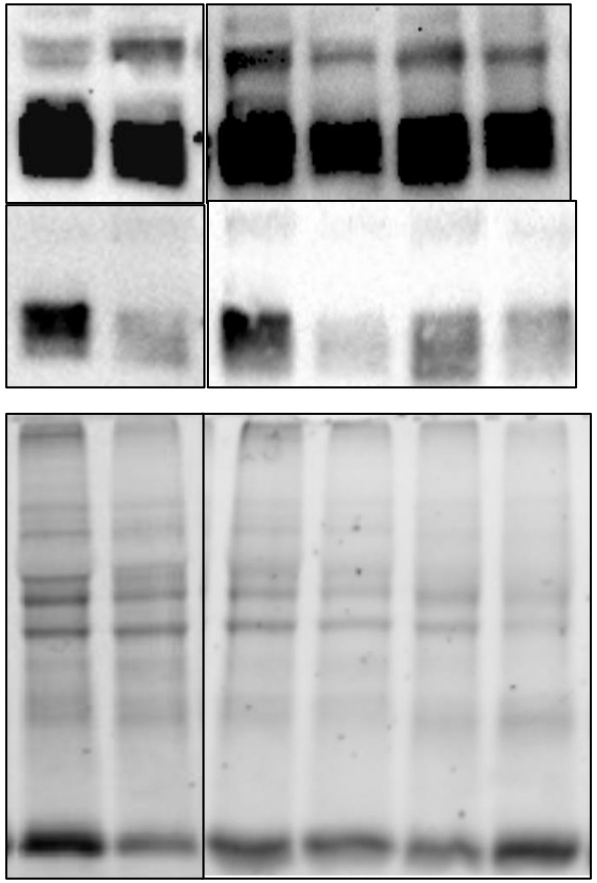
30555 25806 33456
N T N T N T
BNIP3 long exposure
BNIP3 short exposure
TGX

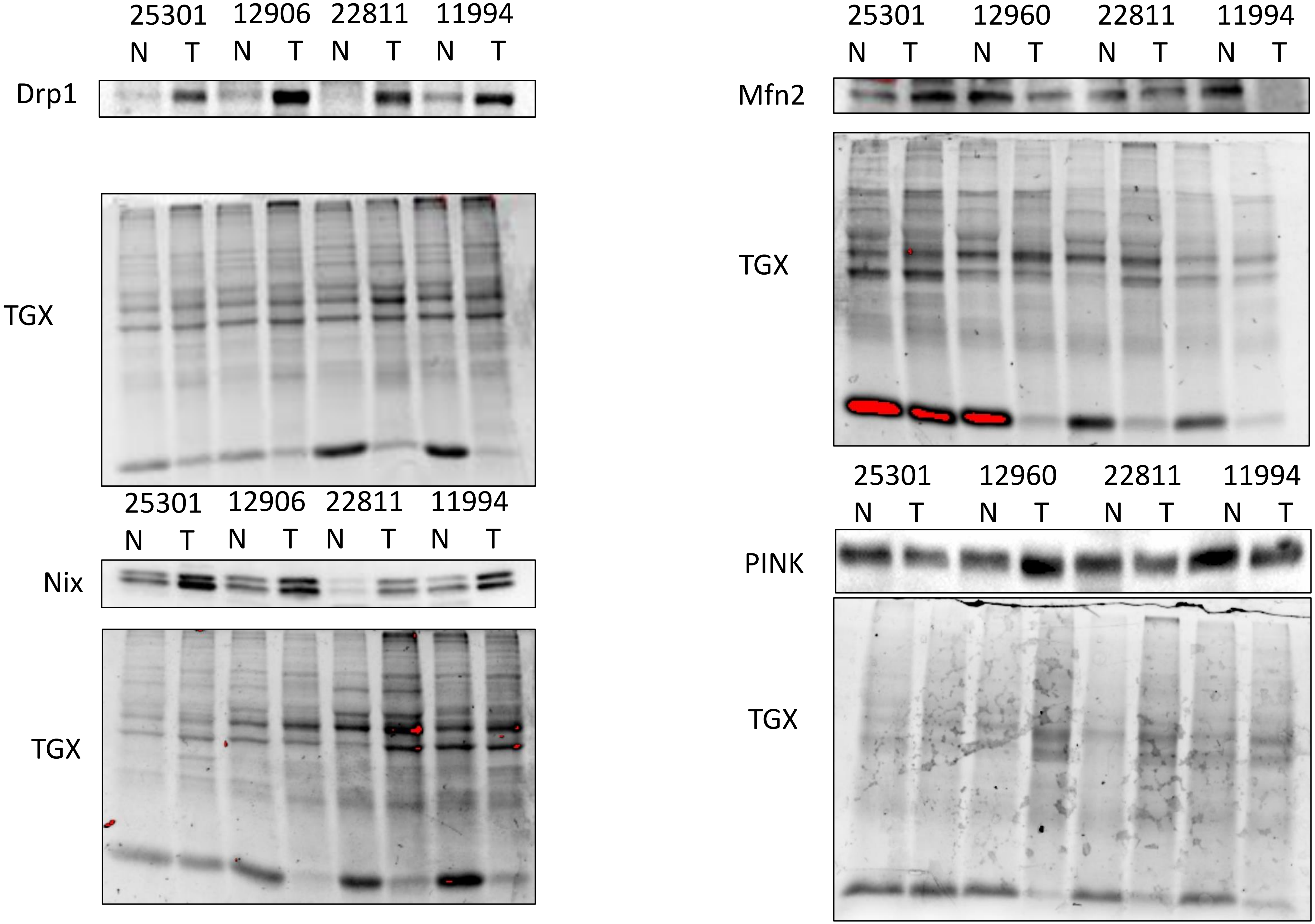
25301 12906 22811 11994
N T N T N T N T
Drp1
TGX
25301 12906 22811 11994
N T N T N T N T
Nix
TGX
25301 12960 22811 11994
N T N T N T N T
Mfn2
TGX
25301 12960 22811 11994
N T N T N T N T
PINK
TGX

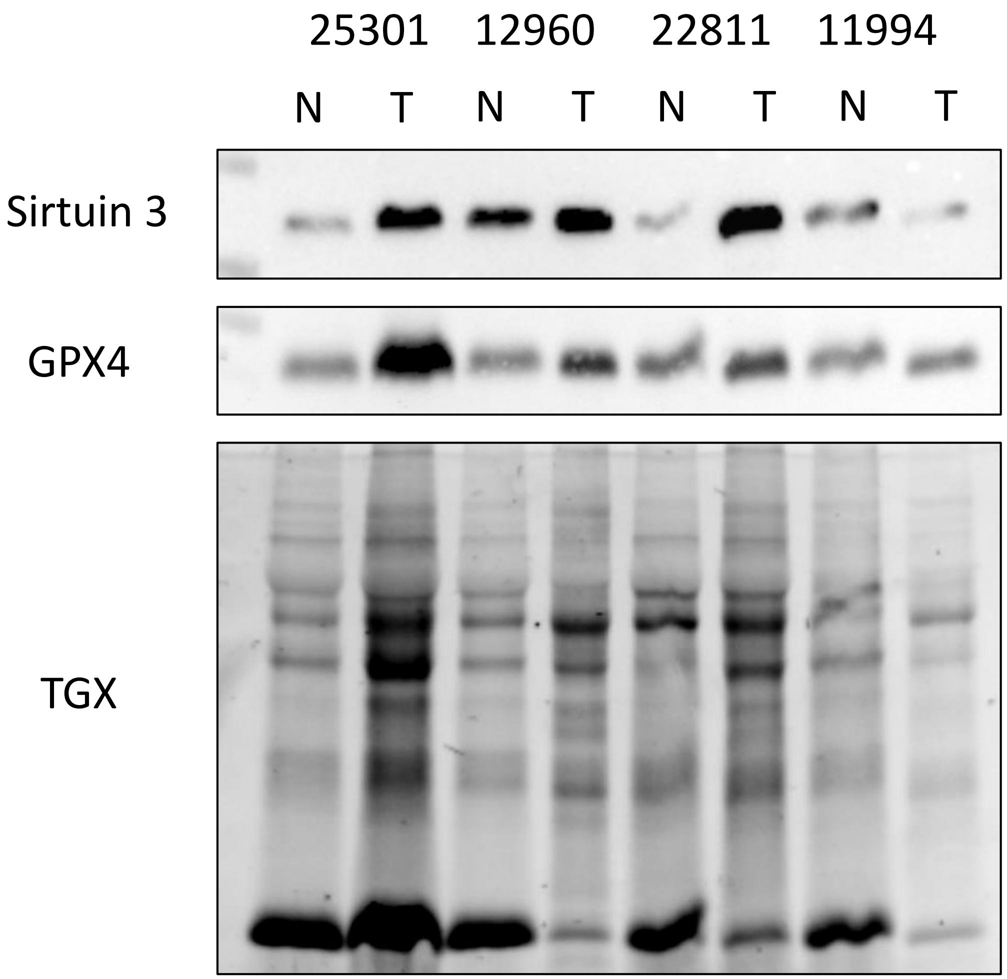
25301 12960 22811 11994
N T N T N T N T
Sirtuin 3
GPX4
TGX

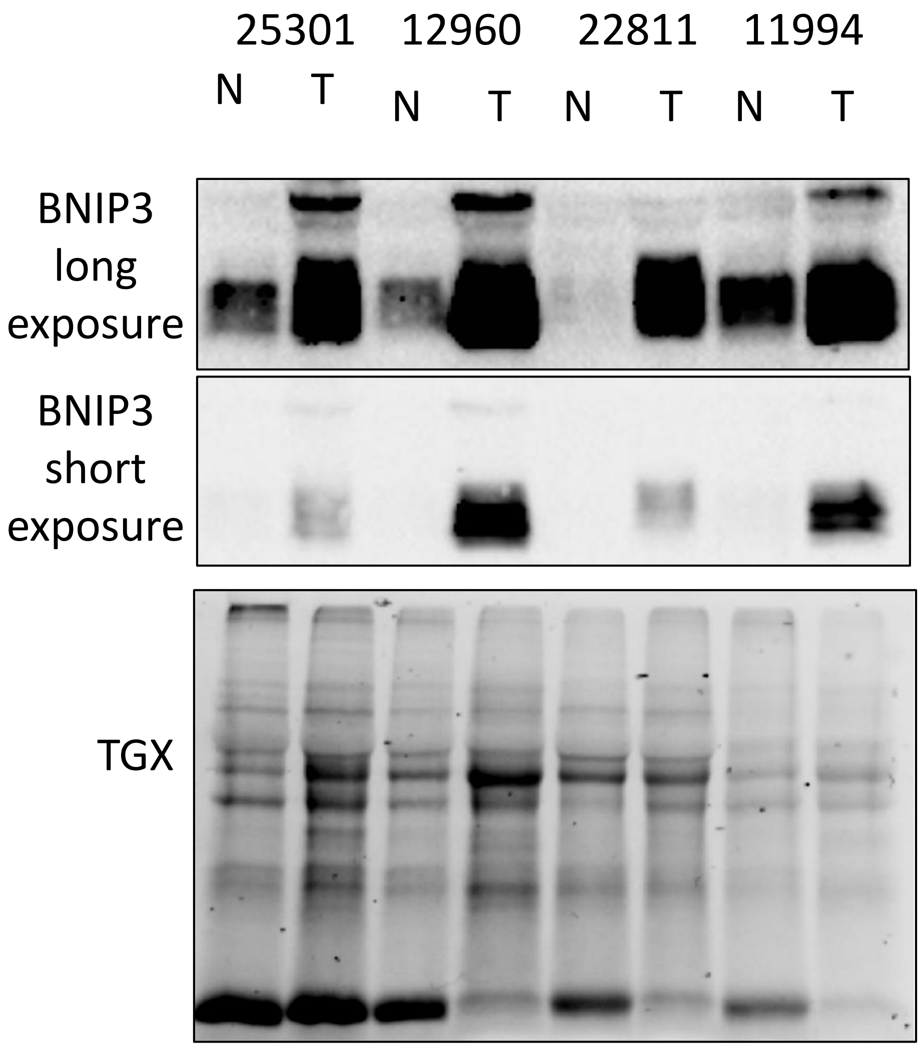
25301 12960 22811 11994
N T N T N T N T
BNIP3 long exposure
BNIP3 short exposure
TGX

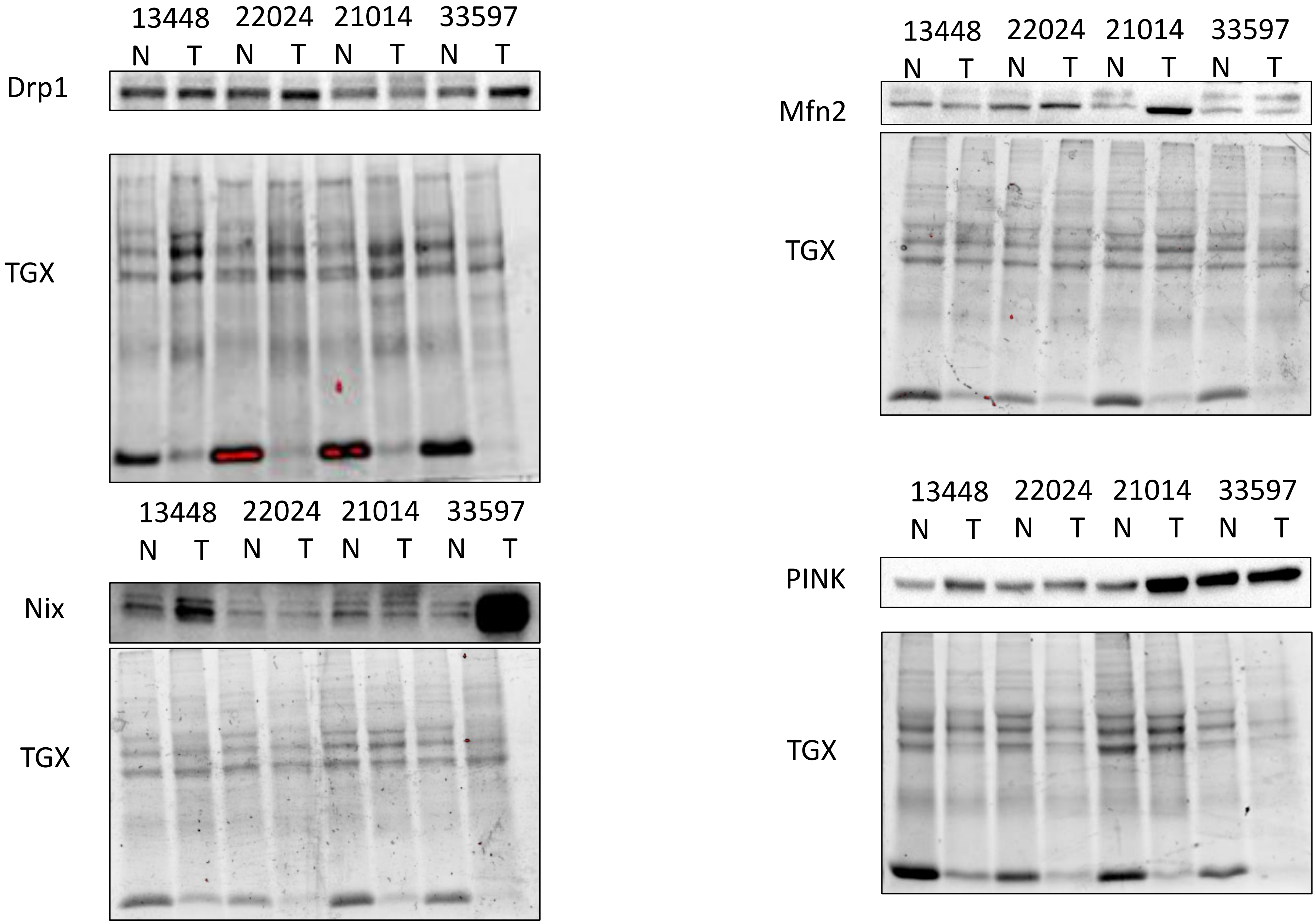
13448 22024 21014 33597
N T N T N T N T
Drp1
TGX
13448 22024 21014 33597
N T N T N T N T
Nix
TGX
13448 22024 21014 33597
N T N T N T N T
Mfn2
TGX
13448 22024 21014 33597
N T N T N T N T
PINK
TGX

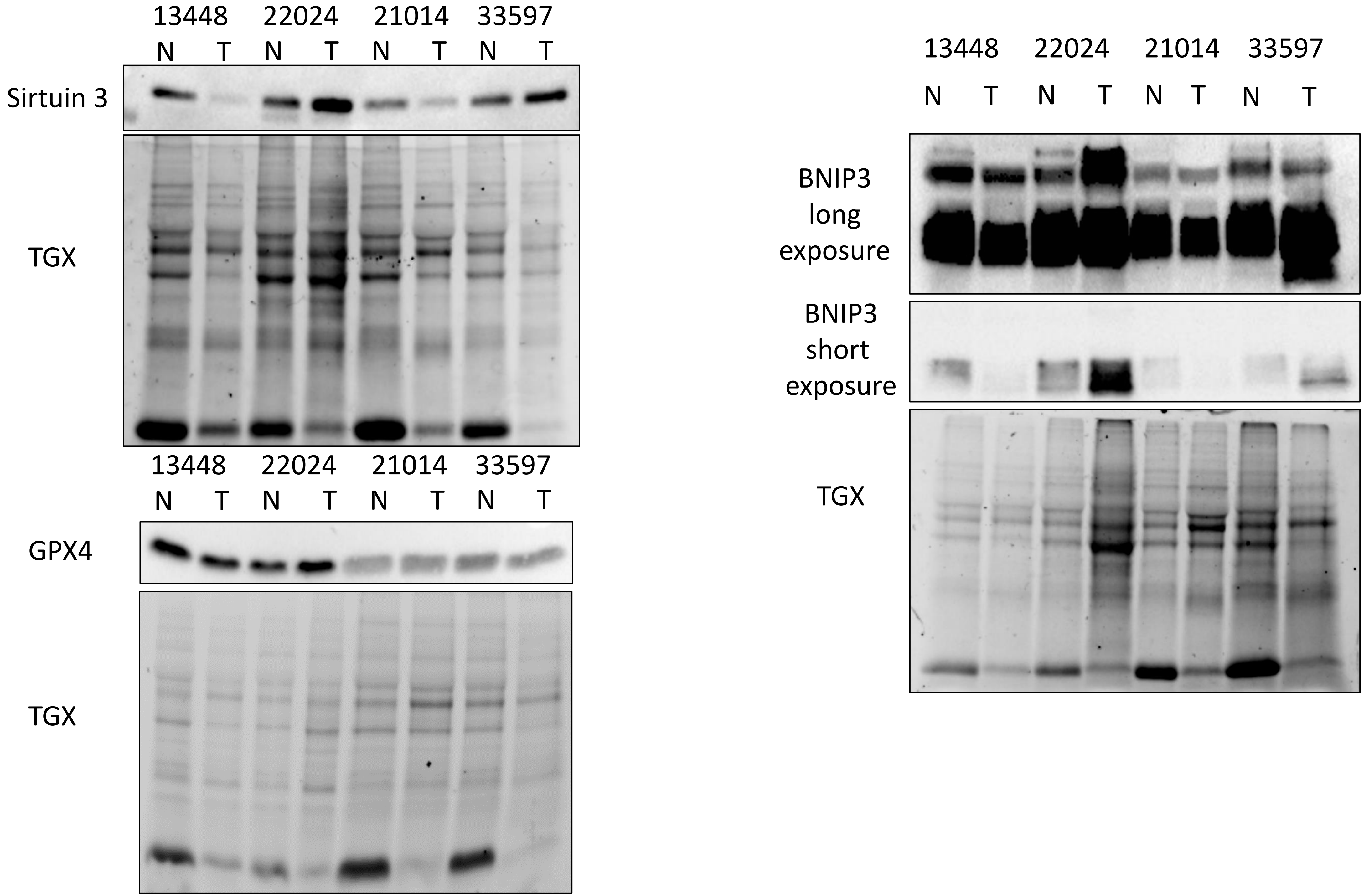
13448 22024 21014 33597
N T N T N T N T
Sirtuin 3
TGX
13448 22024 21014 33597
N T N T N T N T
GPX4
TGX
13448 22024 21014 33597
N T N T N T N T
BNIP3 long exposure
BNIP3 short exposure
TGX

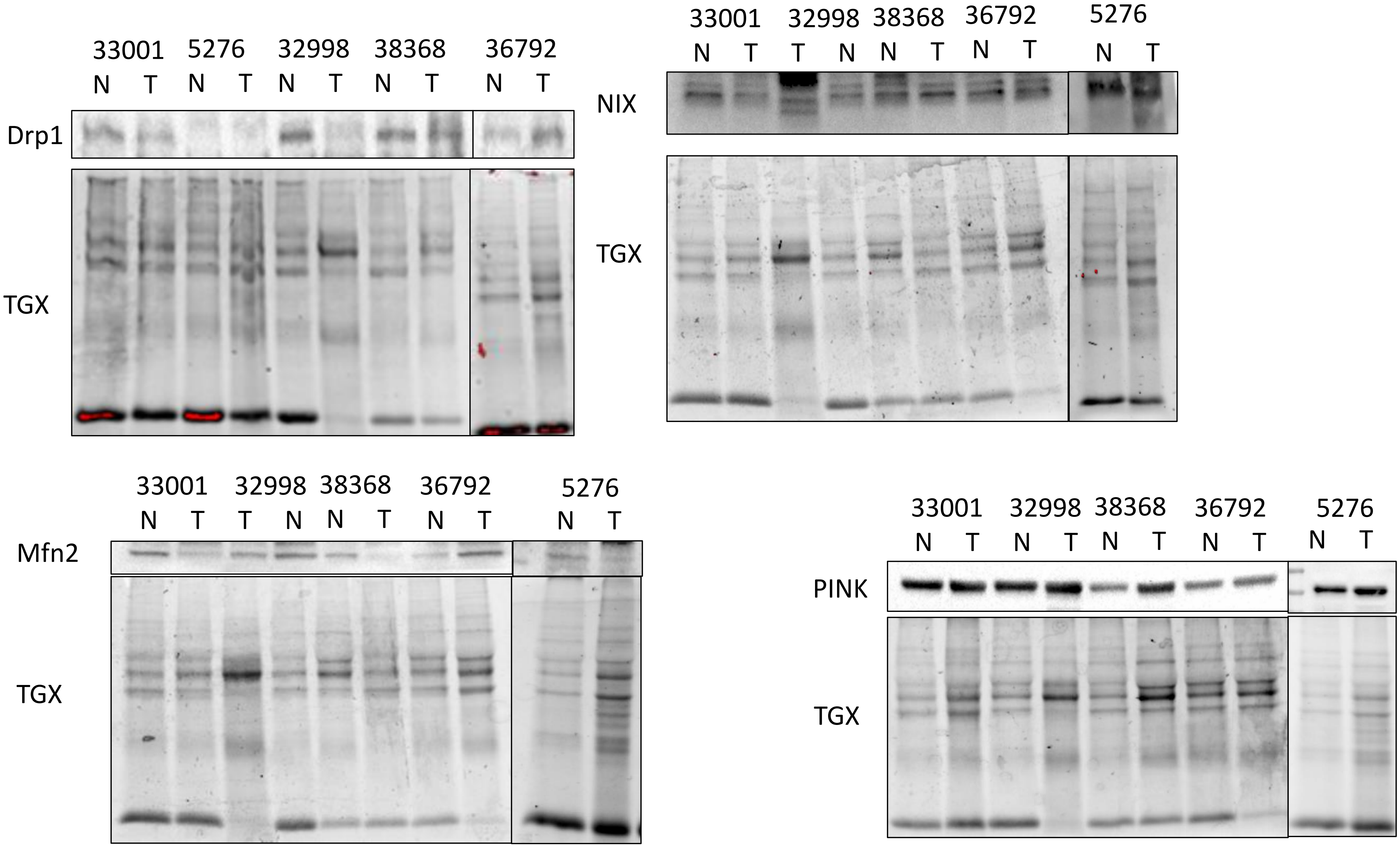
33001 5276 32998 38368 36792
N T N T N T N T N T
Drp1
TGX
33001 32998 38368 36792 5276
N T T N N T N T N T
NIX
TGX
33001 32998 38368 36792 5276
N T T N N T N T N T
Mfn2
TGX
33001 32998 38368 36792 5276
N T N T N T N T N T
PINK
TGX

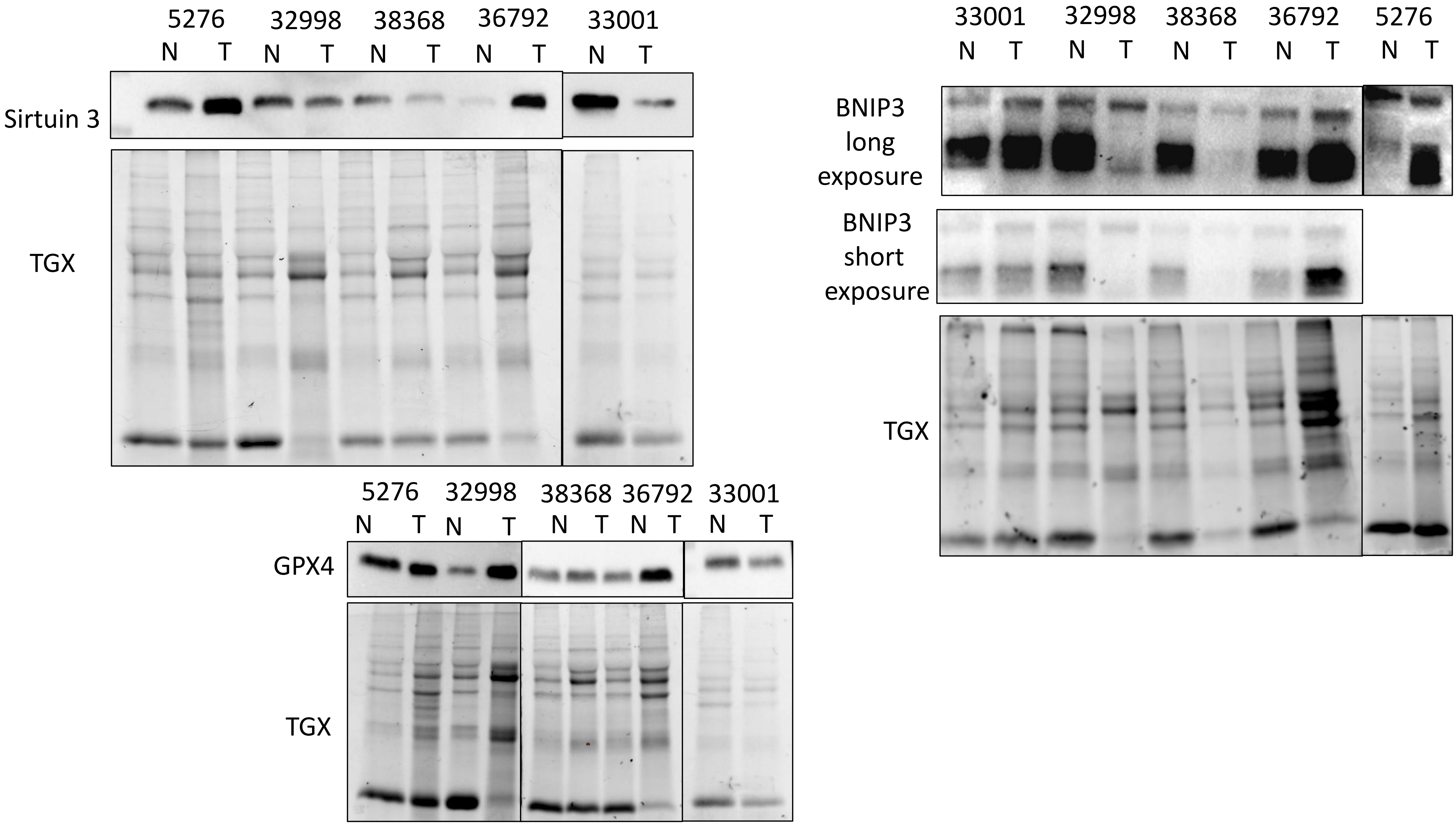
5276
32998
38368
36792
33001
N T N T N T N T N T
Sirtuin 3
TGX
33001
32998
38368
36792
5276
N T N T N T N T N T
BNIP3 long exposure
BNIP3 short exposure
TGX
5276
32998
38368
36792
33001
N T N T N T N T N T
GPX4
TGX

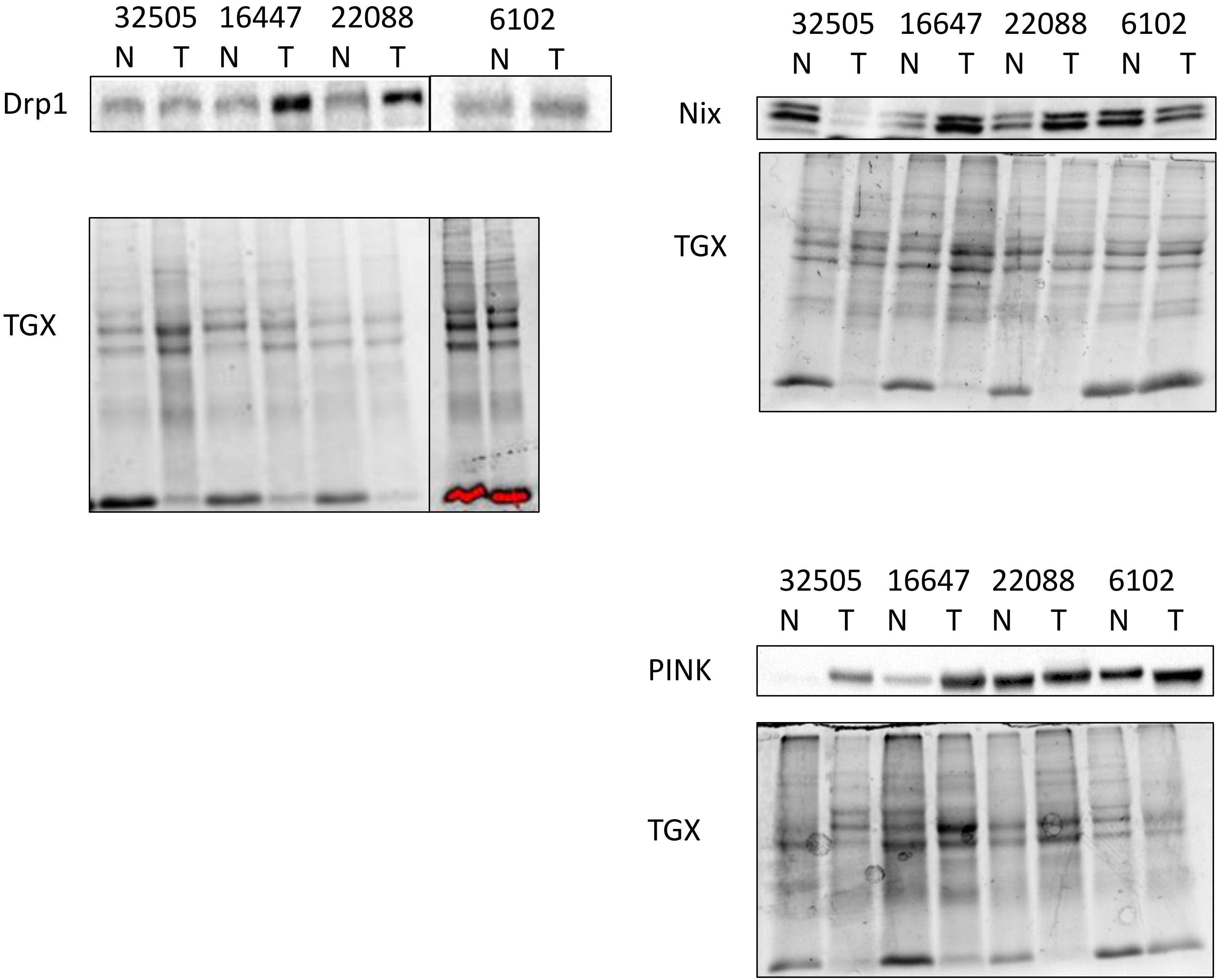

32505 16447 22088 6102
N T N T N T N T
Drp1
TGX
32505 16647 22088 6102
N T N T N T N T
Nix
TGX
32505 16647 22088 6102
N T N T N T N T
PINK
TGX

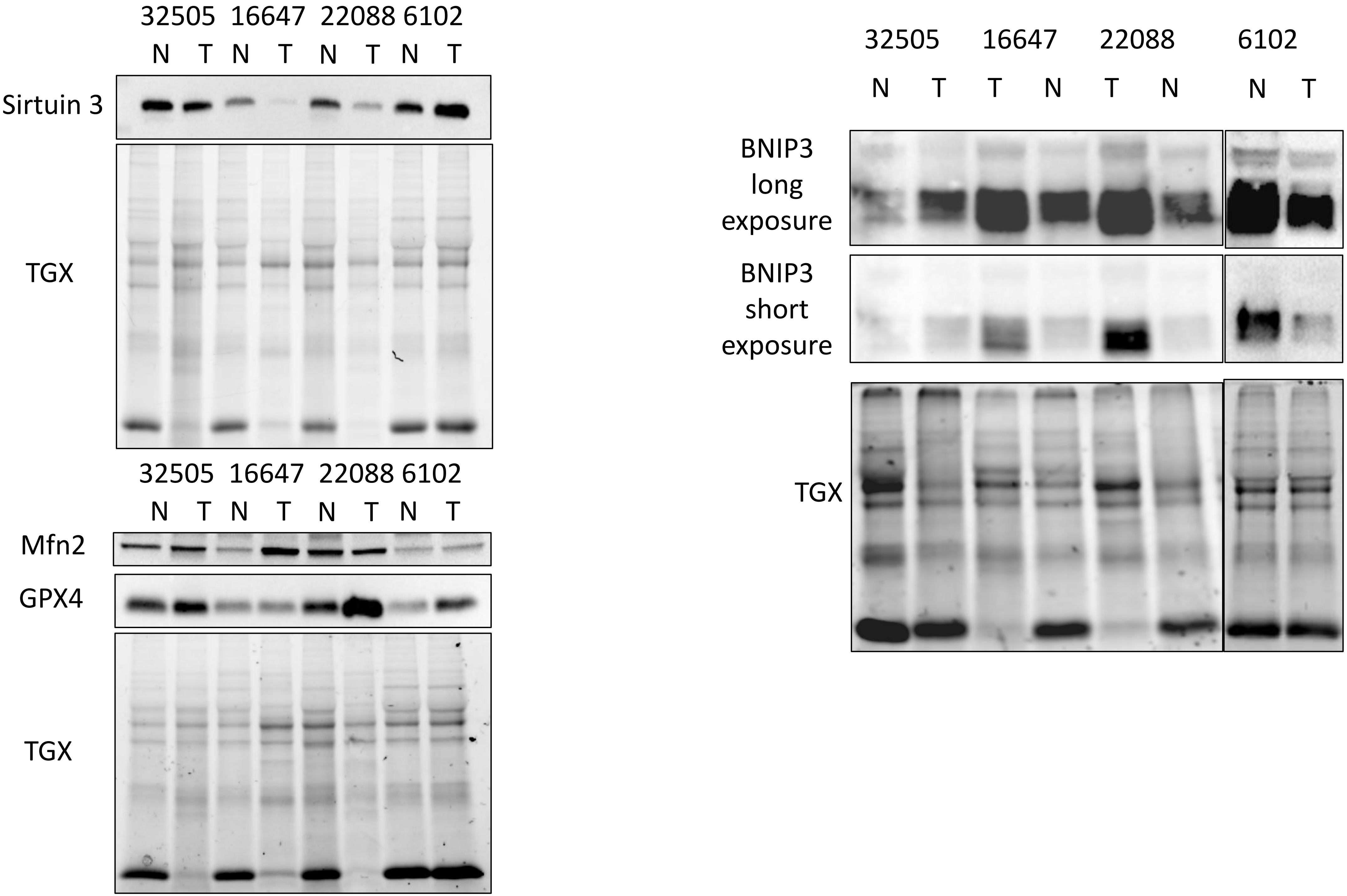
32505 16647 22088 6102
N T N T N T N T
Sirtuin 3
TGX
32505 16647 22088 6102
N T N T N T N T
Mfn2
GPX4
TGX
32505 16647 22088 6102
N T T N T N N T
BNIP3 long exposure
BNIP3 short exposure
TGX

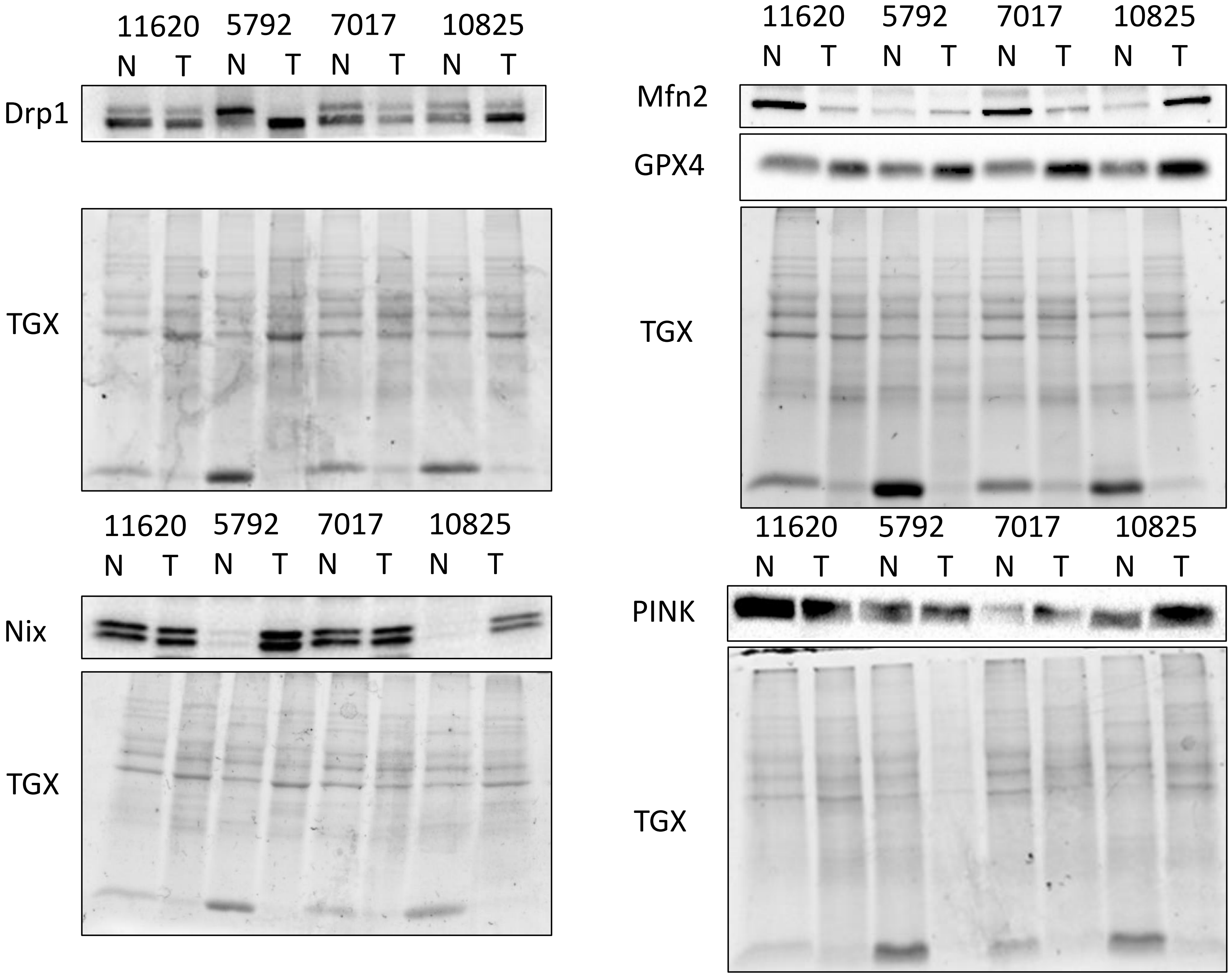
11620 5792 7017 10825
N T N T N T N T
Drp1
TGX
11620 5792 7017 10825
N T N T N T N T
Nix
TGX
11620 5792 7017 10825
N T N T N T N T
Mfn2
GPX4
TGX
11620 5792 7017 10825
N T N T N T N T
PINK
TGX

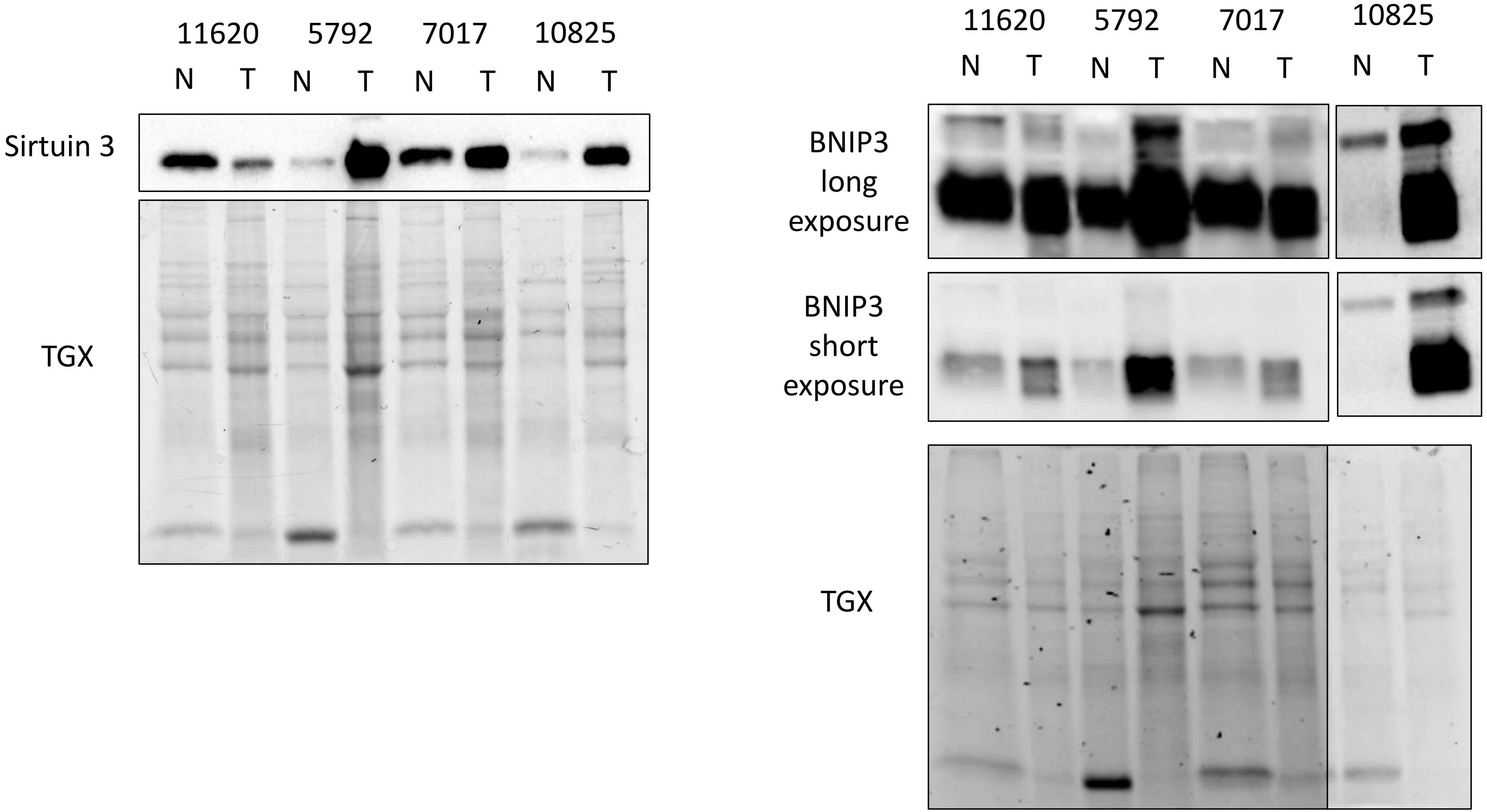
11620
5792
7017
10825
N
T
N
T
N
T
N
T
Sirtuin 3
TGX
11620
5792
7017
10825
N
T
N
T
N
T
N
T
BNIP3 long exposure
BNIP3 short exposure
TGX

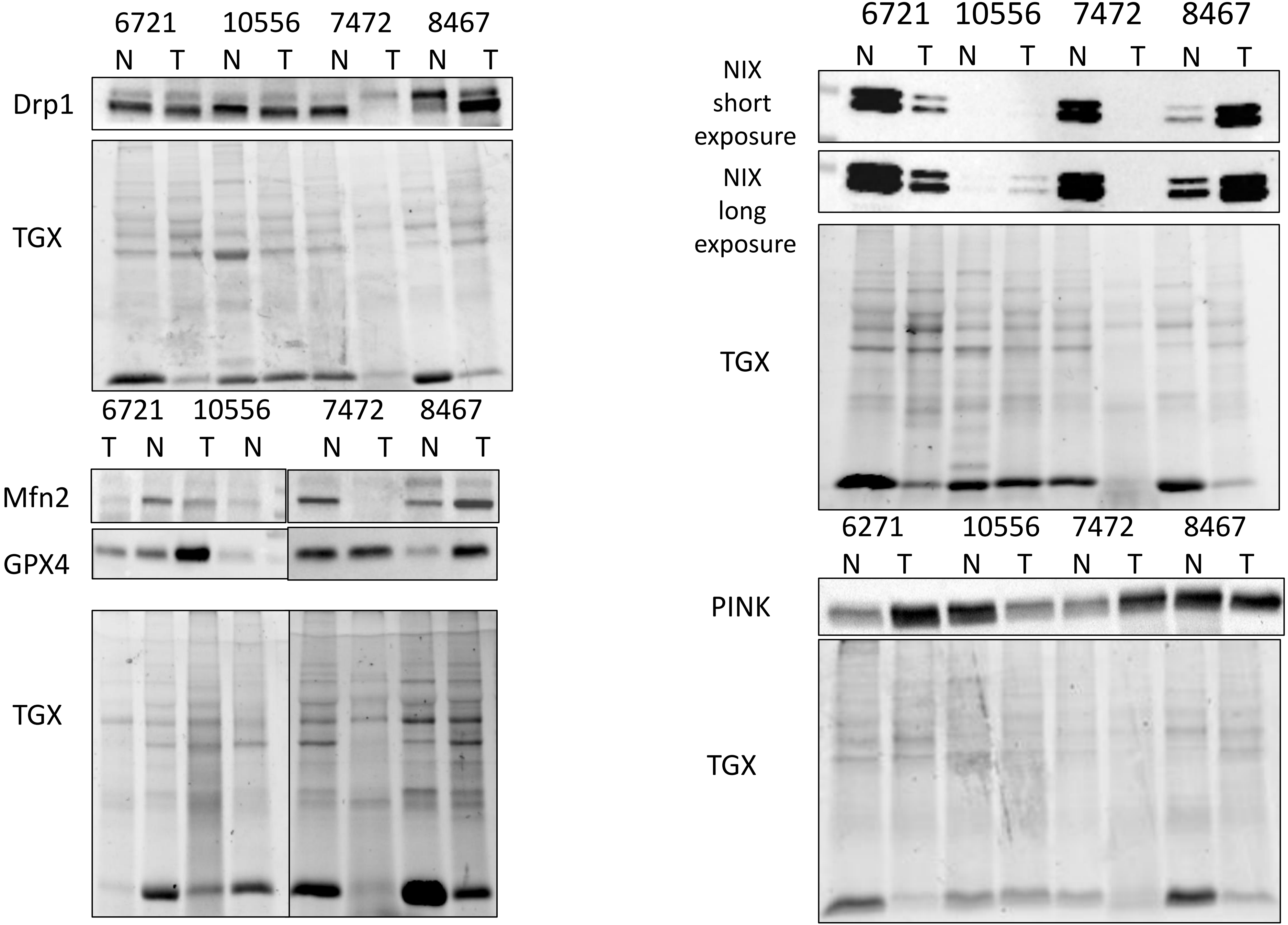
6721 10556 7472 8467
N T N T N T N T
Drp1
TGX
6721 10556 7472 8467
T N T N N T N T
Mfn2
GPX4
TGX
6721 10556 7472 8467
N T N T N T N T
NIX
short
exposure
NIX
long
exposure
TGX
6271 10556 7472 8467
N T N T N T N T
PINK
TGX

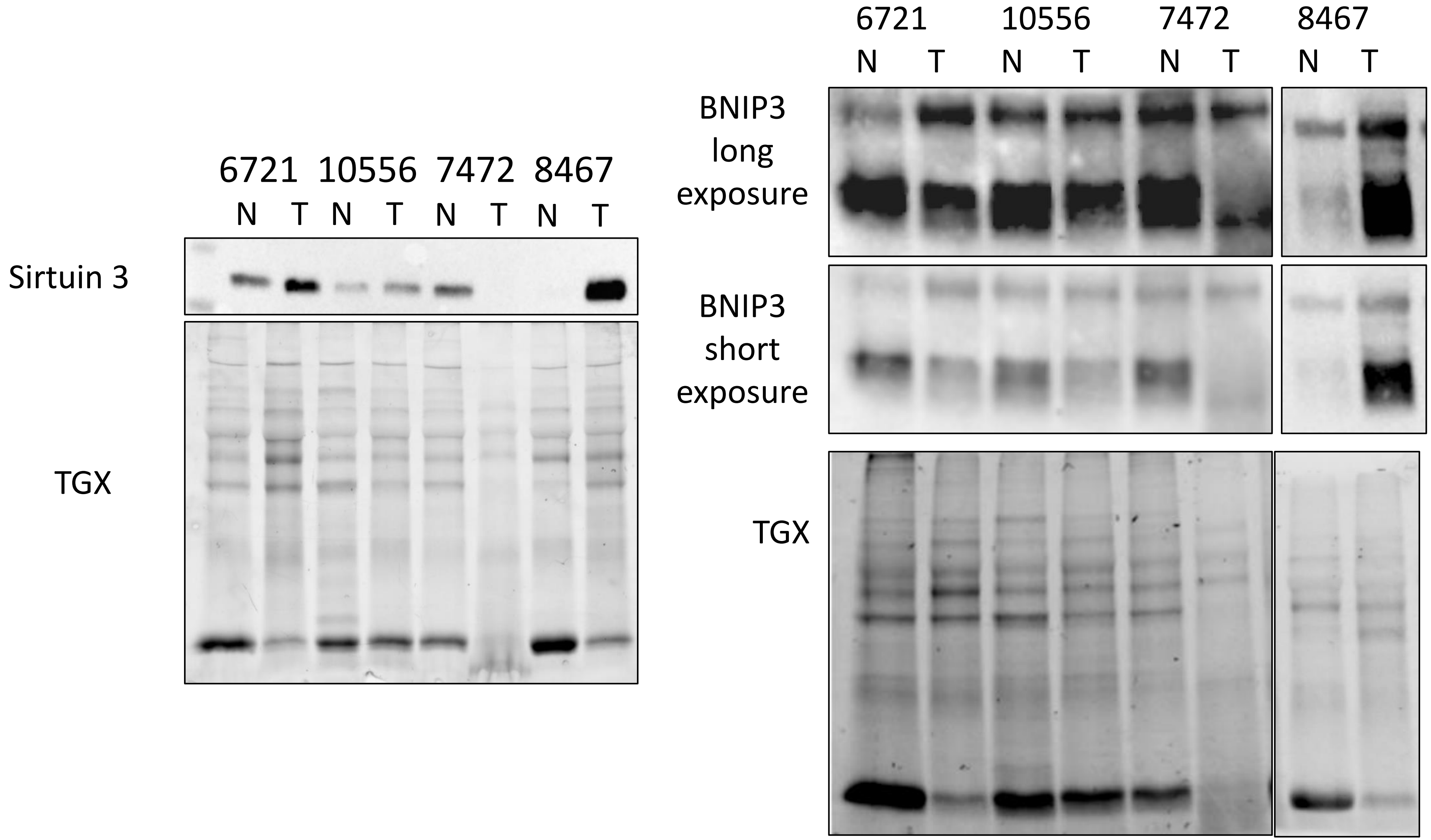

6721 10556 7472 8467
N T N T N T N T
Sirtuin 3
TGX
6721 10556 7472 8467
N T N T N T N T
BNIP3 long exposure
BNIP3 short exposure
TGX

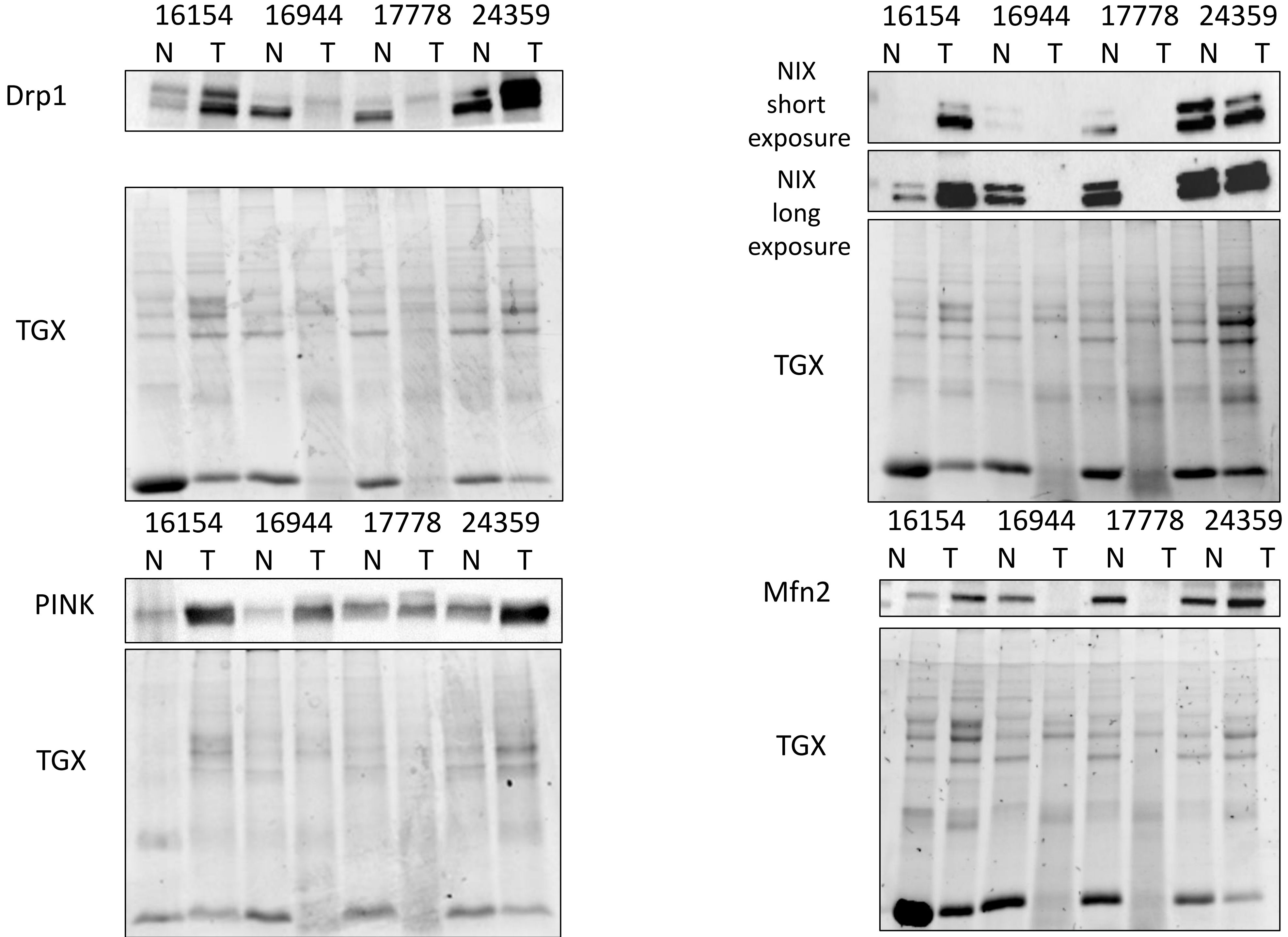
16154 16944 17778 24359
N T N T N T N T
Drp1
TGX
16154 16944 17778 24359
N T N T N T N T
NIX short exposure
NIX long exposure
TGX
16154 16944 17778 24359
N T N T N T N T
PINK
TGX
16154 16944 17778 24359
N T N T N T N T
Mfn2
TGX

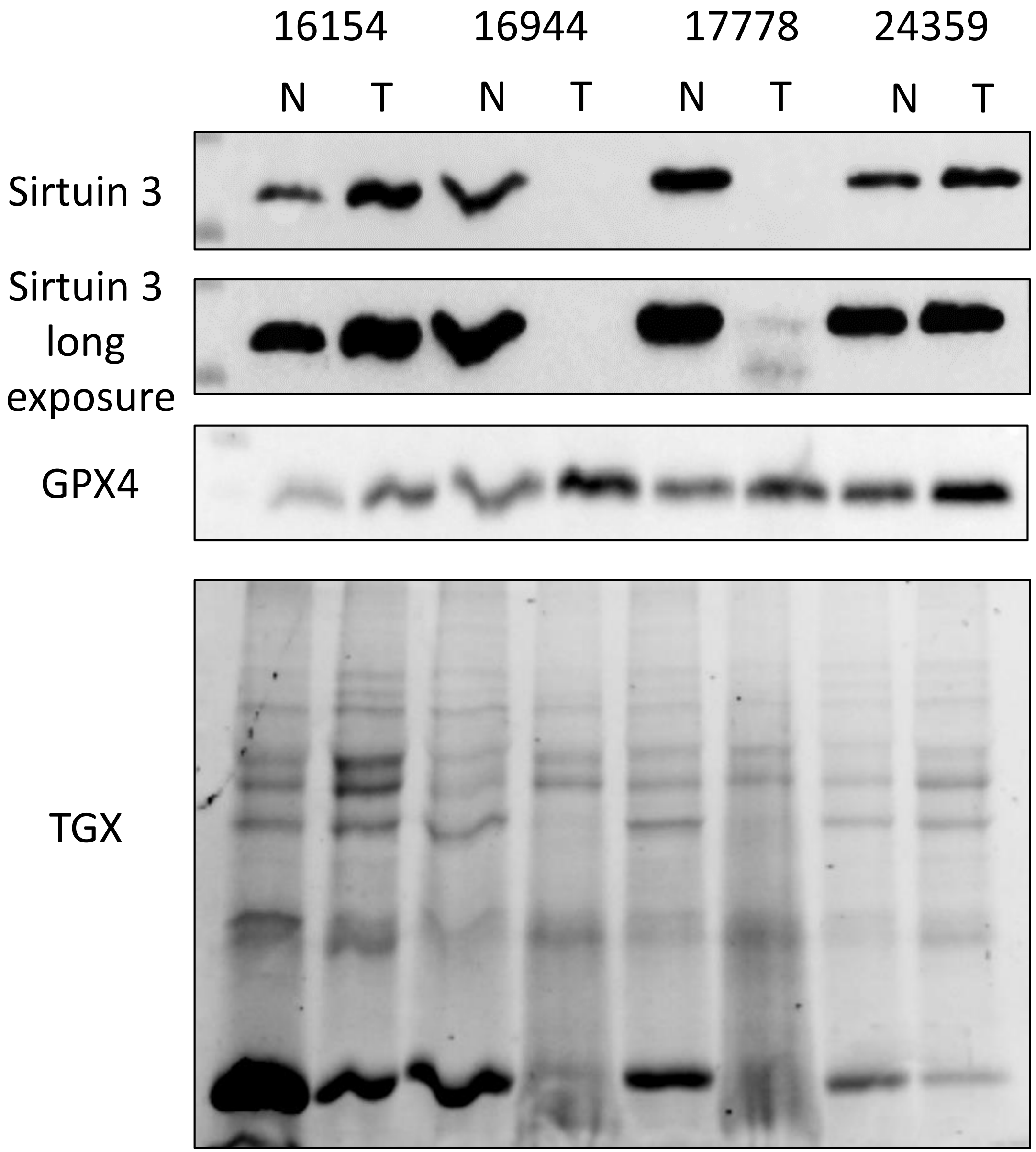
16154
16944
17778
24359
N
T
N
T
N
T
N
T
Sirtuin 3
Sirtuin 3 long exposure
GPX4
TGX

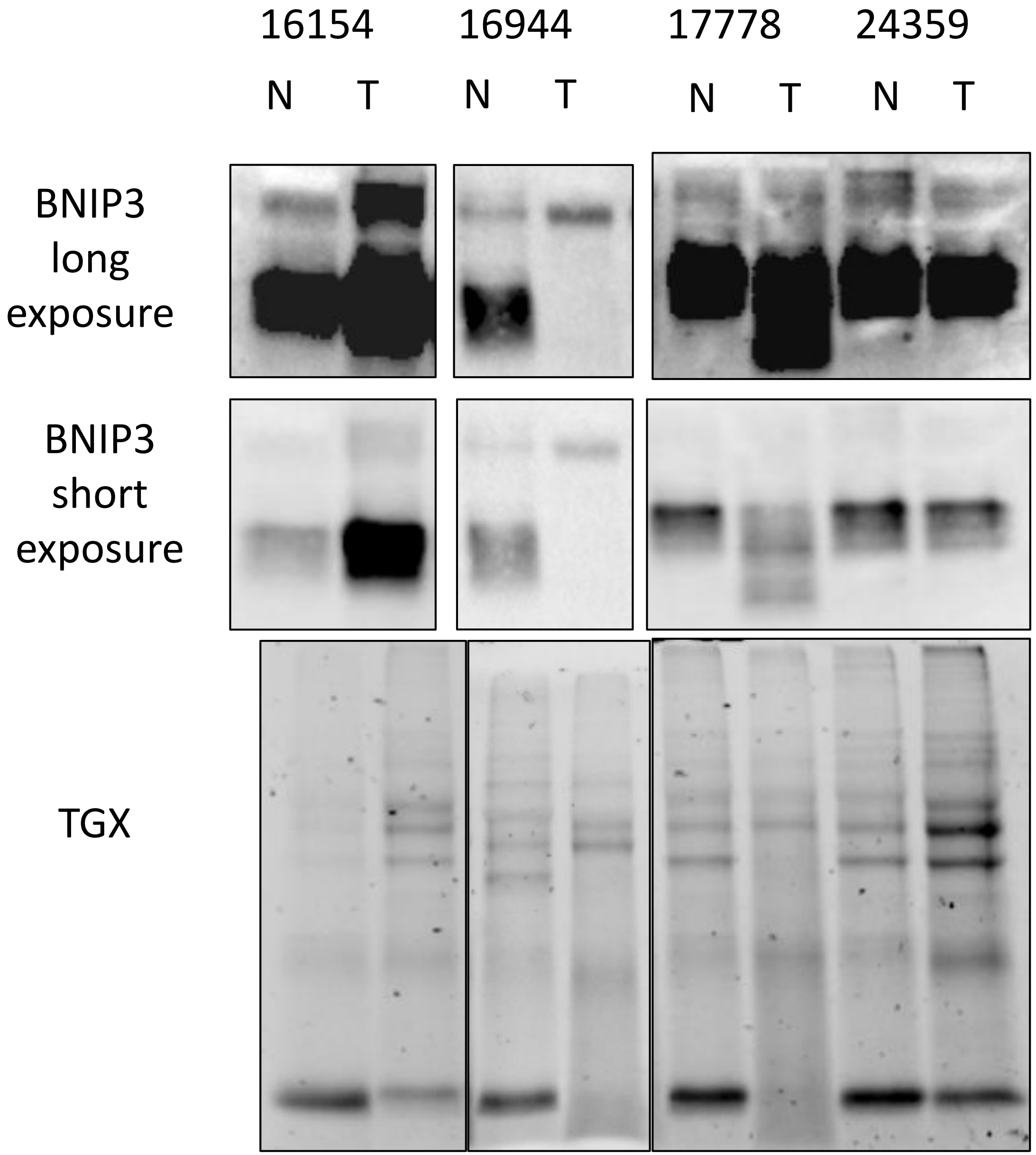
16154
16944
17778
24359
N
T
N
T
N
T
N
T
BNIP3 long exposure
BNIP3 short exposure
TGX

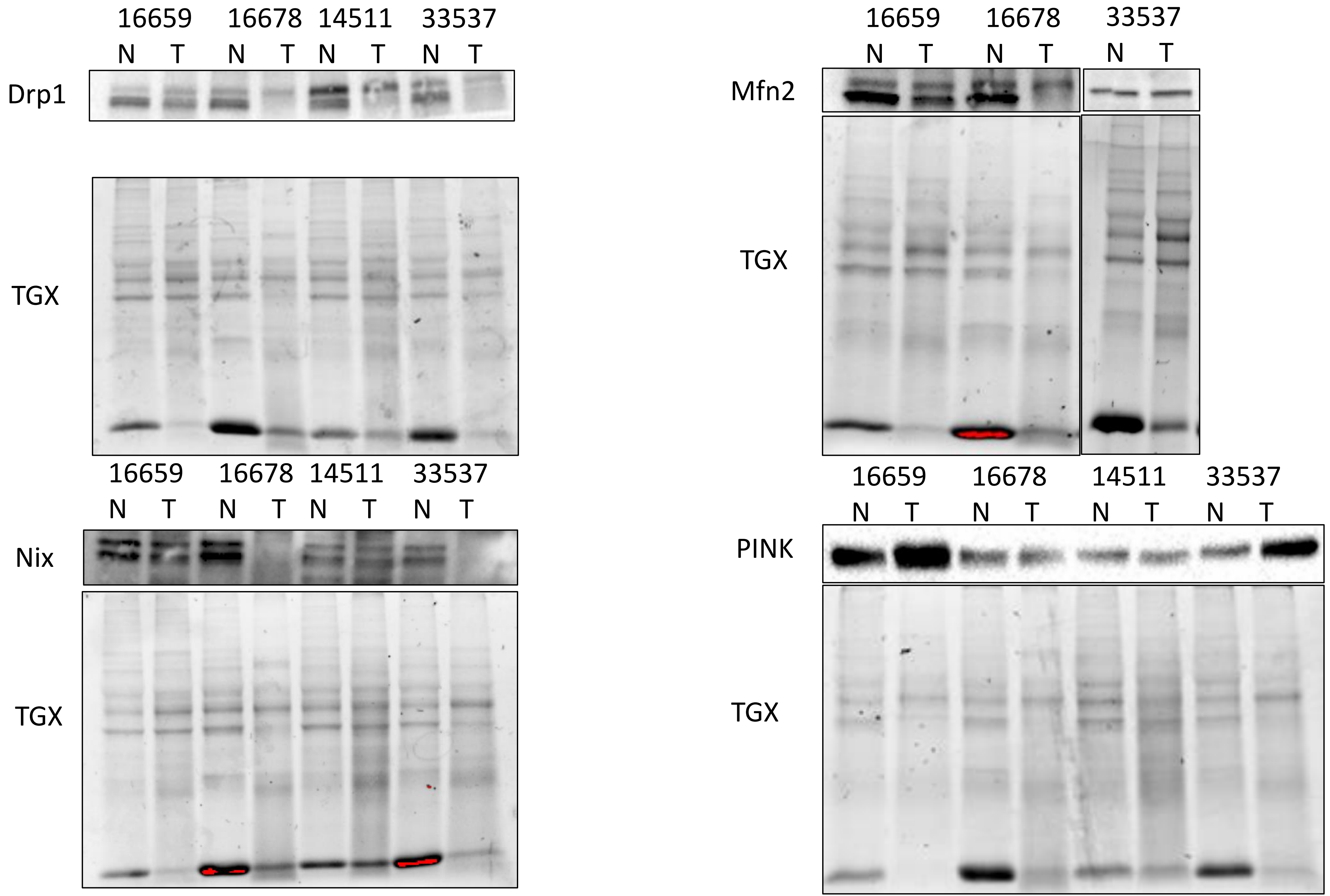
16659 16678 14511 33537
N T N T N T N T
Drp1
TGX
16659 16678 33537
N T N T N T
Mfn2
TGX
16659 16678 14511 33537
N T N T N T N T
Nix
TGX
16659 16678 14511 33537
N T N T N T N T
PINK
TGX

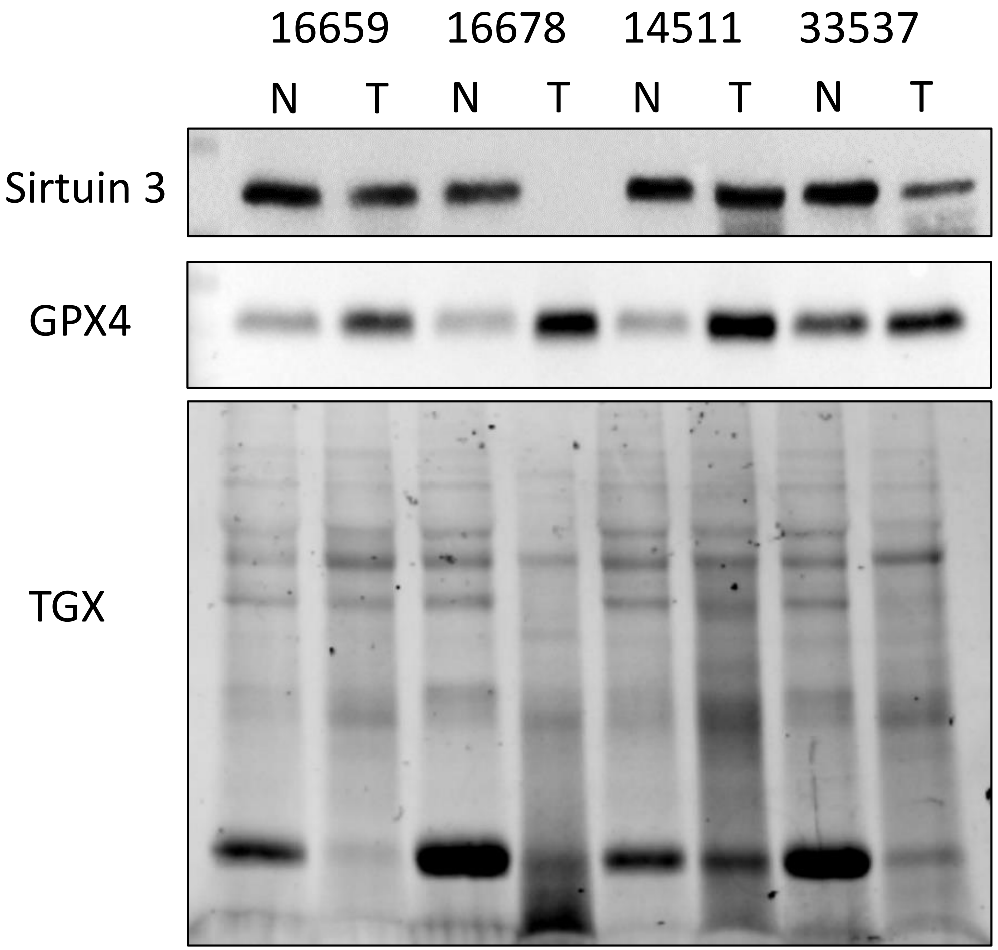
16659 16678 14511 33537
N T N T N T N T
Sirtuin 3
GPX4
TGX

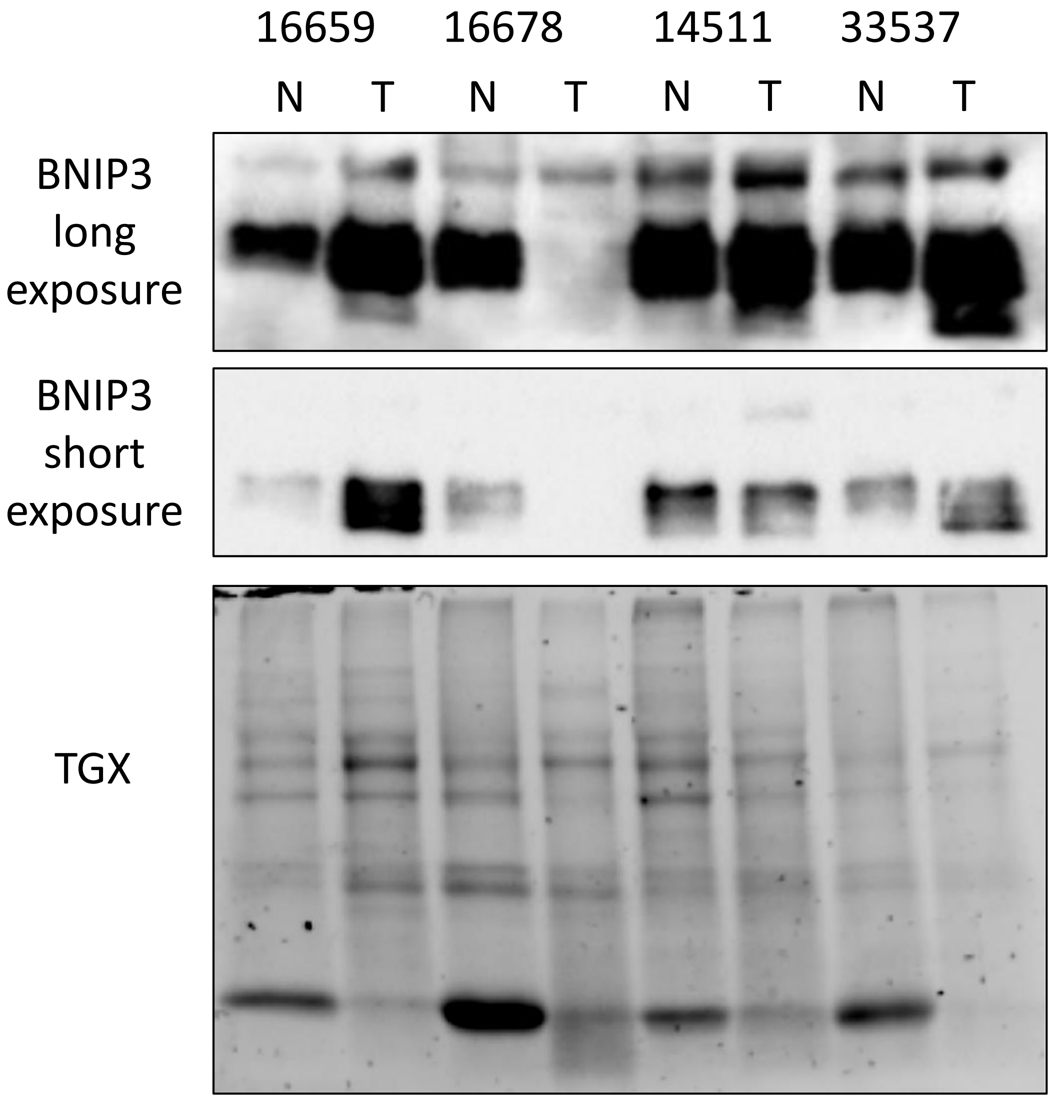
16659 16678 14511 33537
N T N T N T N T
BNIP3 long exposure
BNIP3 short exposure
TGX

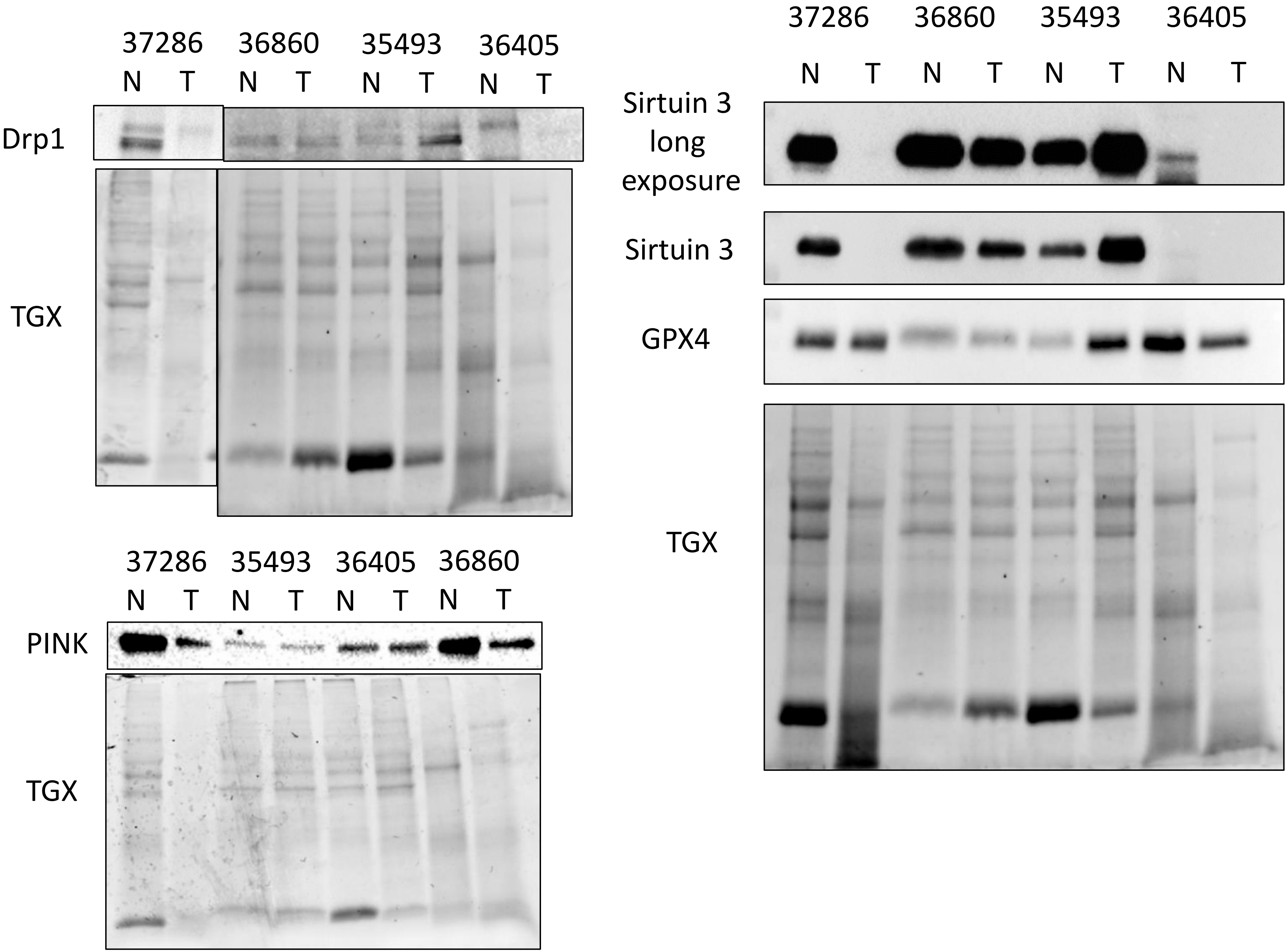
37286
36860
35493
36405
N
T
Drp1
TGX
37286
35493
36405
36860
PINK
TGX
Sirtuin 3
long
exposure
Sirtuin 3
GPX4
TGX

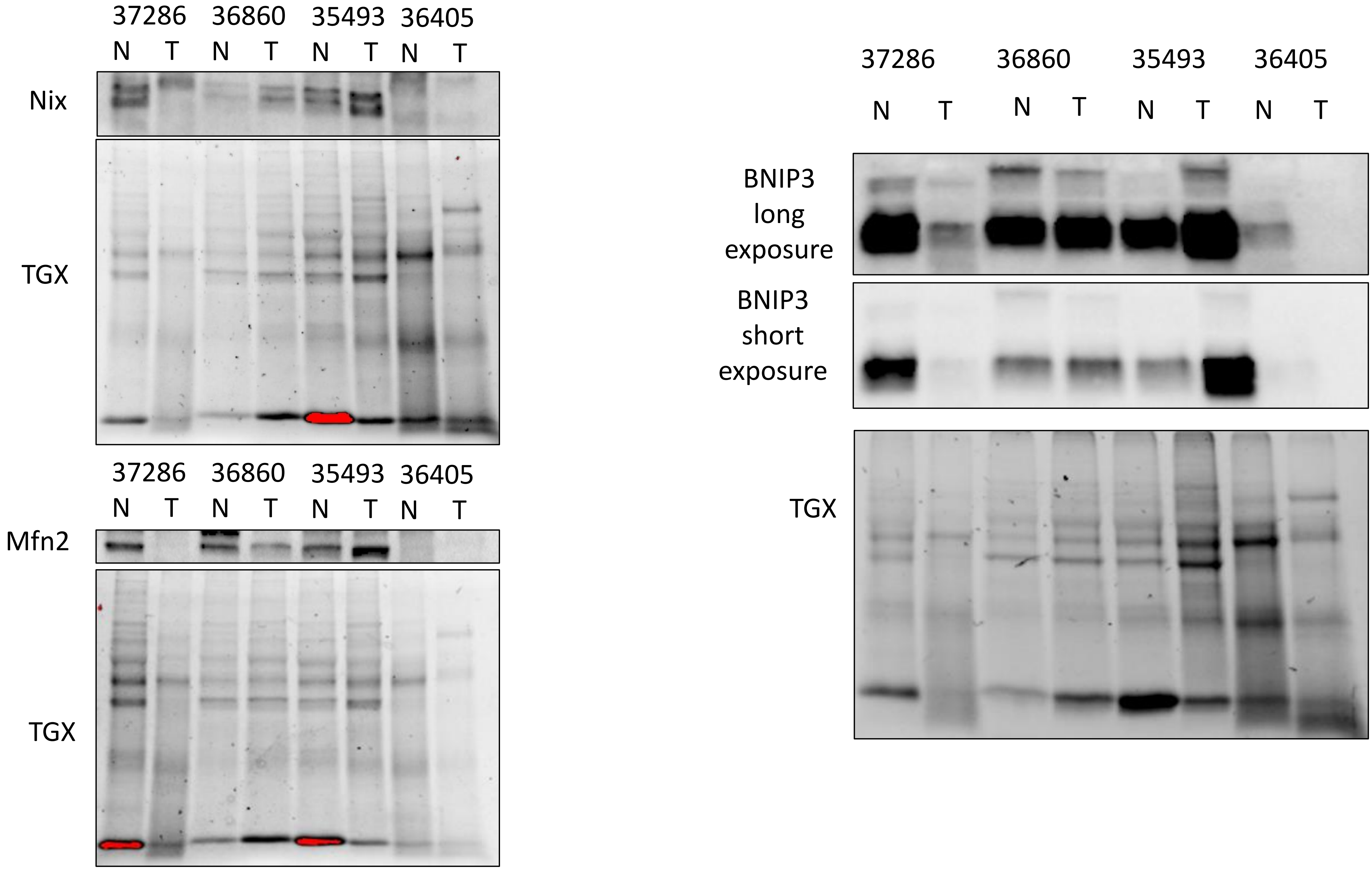
37286 36860 35493 36405
N T N T N T N T
Nix
TGX
37286 36860 35493 36405
N T N T N T N T
Mfn2
TGX
37286 36860 35493 36405
N T N T N T N T
BNIP3 long exposure
BNIP3 short exposure
TGX

Figure S2. The mRNA expression z-scores in cancer samples relative to normal (log RNA Seq V2 RSEM) based on TCGA dataset (LUAD, TCGA, PanCancer Atlas, n=517, p-value < 0,01).

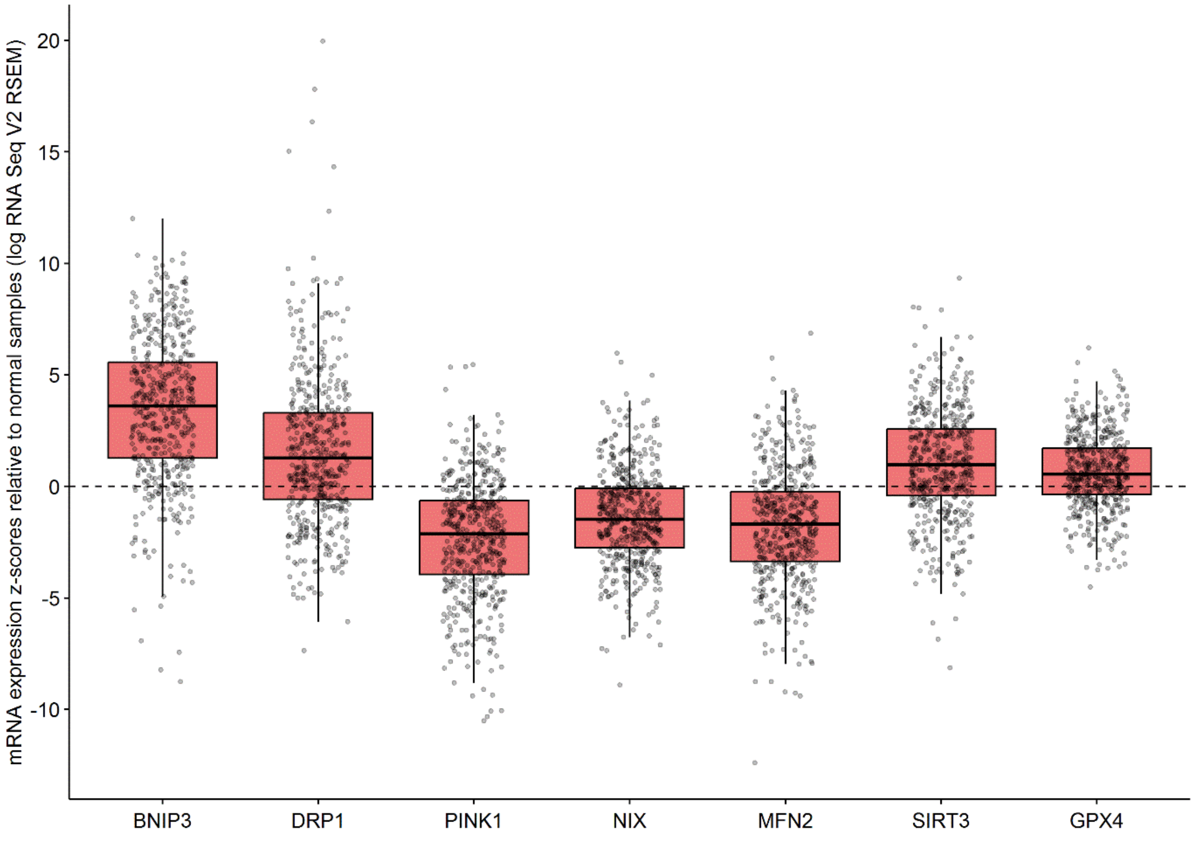

Figure S3. (a) Schoenfeld residual diagram. Individual and global p-values are shown in the diagram. (b) Deviance residual plot.

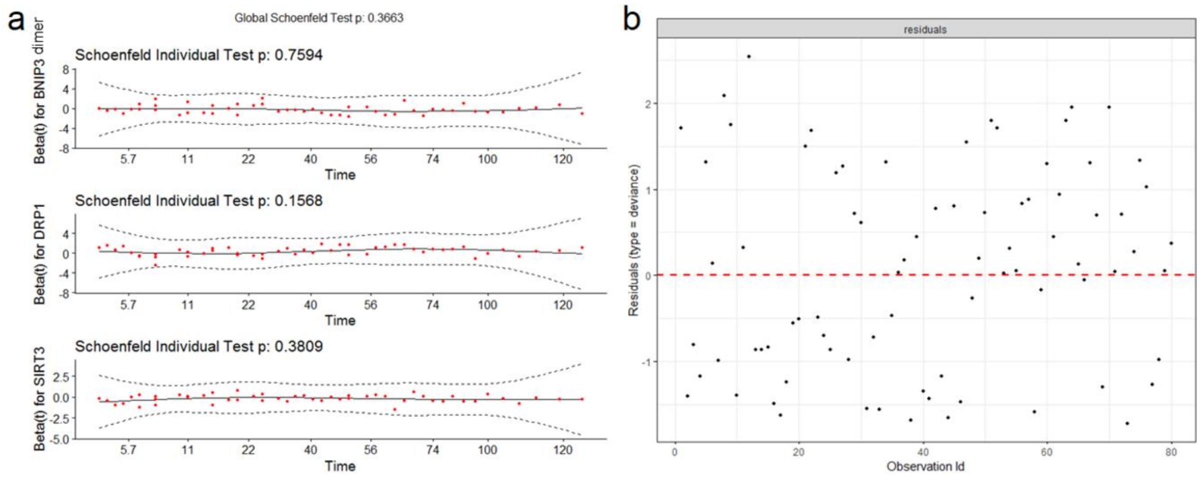

Figure S4. The discriminability of the Cox model (stage + age + risk score) compared with Cox models (risk score and tumor stage) with cross-validation.

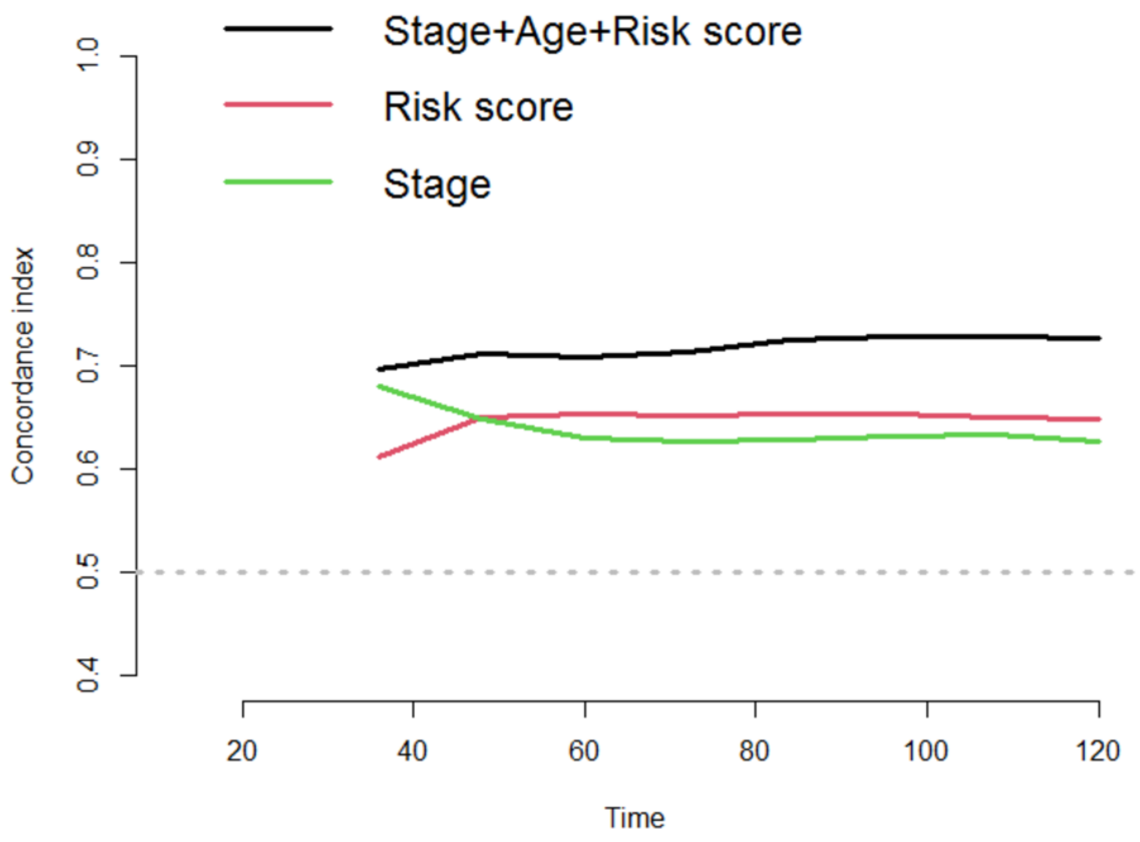

Figure S5. Secondary antibody verification. (a) Stain-free TGX gel with patient tissue and A549 cell lysates. (b) Nitrocellulose membrane with patient tissue and A549 cell lysates after staining with the Cell Signaling Technology and Abcam secondary antibodies. (c) WB analysis of patient tissue samples using anti-Parkin antibodies. Note: 36405, 36860, 37286, 14511, 33537, 37286, 24359, 16659, 10825, and 6721 are patient IDs. N, adjacent non-tumorous tissue; T, tumorous tissue; TGX, TGX Stain-Free^{TM} FastCast^{TM} Gel

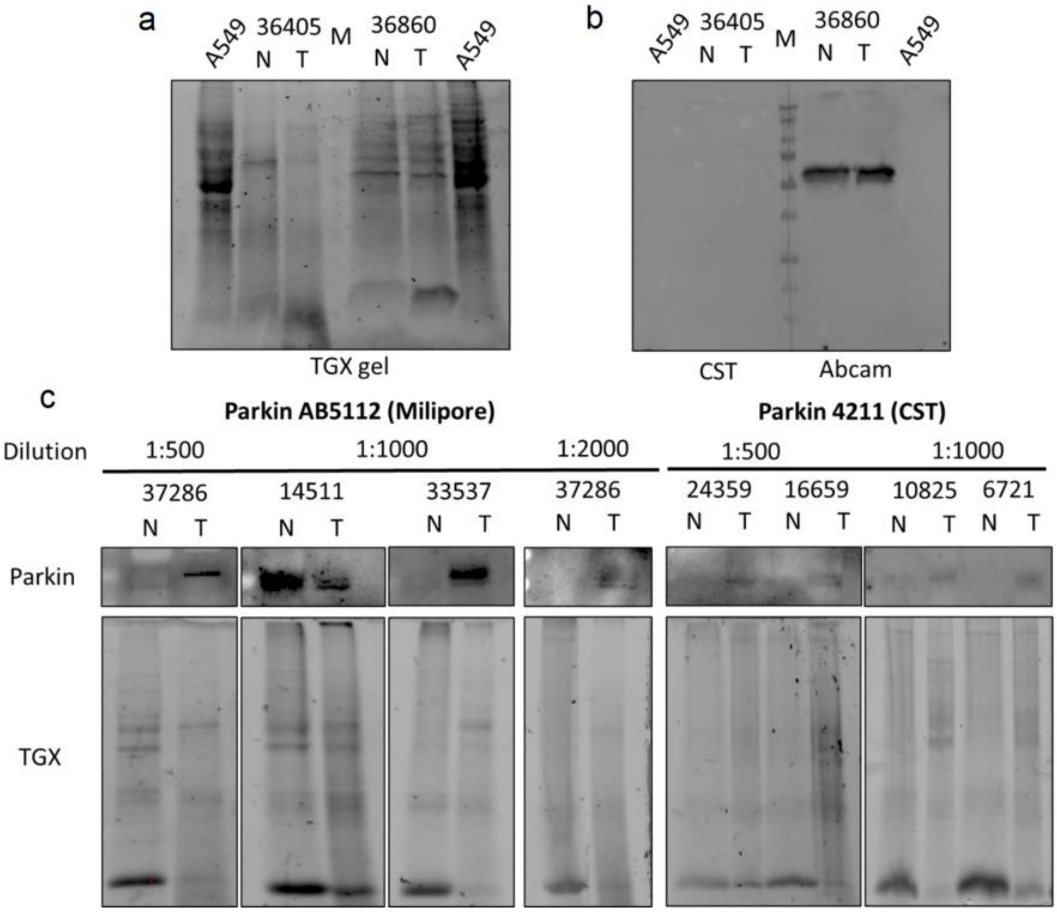

Table S1. Clinicopathological data and normalized MQCP levels in tumorous to non-tumorous tissues.

Patient's number		Clinicopathological features									Overall survival		The ratio of normalized MQCP levels in tumorous to non-tumorous tissues transformed into a log2 scale							
№	Patient's ID	T	N	M	Grade	Stage	Sex	Age	Age category	Multiple primary malignant tumours	Overall survival (1-dead, 0-alive)	Overall survival (months)	BNIP3 dimer	BNIP3 monomer	DRP1	PINK1	BNIP3L/NIX	MFN2	SIRT3	GPX4
1	12259	1-2	0	0	1-2	1-2	male	66	> 65	absence	1	25	3,433973	4,147885	0,384	0,6741	-0,64899	-0,72509	2,014677	0,885133
2	14024	1-2	0	0	mixed	1-2	male	62	≤ 65	absence	1	64	-1,17481	0,298916	0,2989	-0,3079	-3,60441	1,00711	-5,79348	-1,01343
3	15072	3-4	1-2	0	3-4	3-4	male	60	≤ 65	absence	0	97	1,508039	2,082768	-0,2593	1,5047	0,49621	0,207474	2,977091	-0,49027
4	17628	1-2	1-2	0	3-4	3-4	male	61	≤ 65	absence	0	85	0,528278	1,790665	1,7907	0,1282	0,4335	-0,90446	3,496035	1,394592
5	18607	1-2	1-2	0	1-2	3-4	male	52	≤ 65	absence	1	20	-1,53722	1,258433	-0,702	0,459	-0,28635	1,778162	2,484324	-0,18469
6	19547	1-2	1-2	0	3-4	1-2	male	81	> 65	absence	1	53	-0,54826	2,326322	2,3263	-0,5482	0,91639	-0,3596	1,803282	1,174069
7	2226	1-2	1-2	0	3-4	1-2	male	60	≤ 65	absence	0	80	0,308119	0,587988	-0,3766	-1,9828	0,72067	1,889777	1,720207	0,524796
8	23414	1-2	1-2	0	mixed	1-2	male	73	> 65	absence	1	2	0,696654	2,16775	2,1678	-0,9824	0,43577	1,460982	-0,44378	1,028785
9	2483	3-4	0	0	3-4	1-2	male	73	> 65	absence	1	11	2,238399	1,41665	-0,0861	0,8218	0,70702	-0,09872	0,128859	1,427861
10	26763	1-2	0	0	1-2	1-2	male	65	≤ 65	absence	0	81	1,823304	1,758716	1,7587	0,306	-0,56578	2,184105	-0,21375	2,125772
11	28655	1-2	1-2	0	mixed	3-4	female	74	> 65	absence	1	80	0,675344	0,878231	1,0522	0,0956	1,37285	0,271297	1,679785	1,397932
12	29384	3-4	0	0	3-4	1-2	female	69	> 65	absence	1	0	1,209201	1,915624	1,9156	0,474	0,47011	0,603435	0,362887	-0,71035
13	34459	1-2	0	0	3-4	1-2	male	64	≤ 65	absence	0	96	1,960913	2,186792	-0,3277	1,0536	1,23321	-0,88623	1,514102	0,919238
14	35050	3-4	1-2	0	1-2	3-4	female	53	≤ 65	absence	0	54	0,701138	0,118394	0,1184	-0,7402	-0,67373	-1,55502	1,090256	0,79677
15	36137	3-4	0	0	1-2	1-2	male	64	≤ 65	absence	0	86	-0,33851	1,432187	0,2637	0,2479	0,62457	0,881166	5,546586	-0,28757
16	4840	1-2	1-2	0	1-2	1-2	female	55	≤ 65	absence	0	104	2,485902	2,389644	2,3896	-0,2218	2,08184	-0,25151	0,183299	3,197515
17	5897	1-2	0	0	1-2	1-2	male	64	≤ 65	absence	0	79	0,475837	0,862099	-0,0439	-3,119	-1,37695	-2,48372	-3,06055	-0,4211
18	16327	3-4	0	0	3-4	1-2	male	64	≤ 65	absence	0	58	1,388122	1,737481	1,7375	-3,1003	0,04126	0,313346	-0,73173	0,378613
19	44020	3-4	0	0	1-2	1-2	female	63	≤ 65	presence	0	13	2,361367	1,298688	1,2987	-0,5989	-1,46475	-0,48214	-0,07083	0,833143
20	16140	1-2	0	0	1-2	1-2	female	69	> 65	presence	0	39	0,446896	0,404375	-2,4248	-0,395	0,8404	-0,76285	1,008769	1,32085
21	42490	3-4	1-2	0	1-2	3-4	male	75	> 65	absence	1	5	-0,71703	0,97021	0,9702	-0,2073	-0,51065	0,879974	-2,6038	-0,21471
22	39655	3-4	1-2	0	3-4	3-4	female	63	≤ 65	absence	1	9	-0,08783	-0,36747	-0,3675	0,0605	-0,58182	-0,13926	0,272514	0,307686
23	30555	3-4	0	0	3-4	1-2	female	54	≤ 65	absence	0	33	1,117085	-1,04388	-1,2289	-0,2368	0,1079	-0,97309	1,904757	0,992934
24	33456	1-2	0	0	1-2	1-2	male	68	> 65	absence	0	30	-0,13057	-0,59669	-0,1153	-0,7703	-0,28669	-0,12466	1,73288	0,059047

25	12960	3-4	0	0	1-2	1-2	male	61	≤ 65	absence	0	35	1,331956	0,707734	0,827	-0,4668	0,52174	-0,71143	-0,39818	-0,48694
26	11994	3-4	1-2	1	3-4	3-4	male	61	≤ 65	absence	1	20	1,628867	0,987737	0,2784	0,1124	1,26469	-1,08045	-1,00617	0,679077
27	22024	1-2	0	0	3-4	1-2	male	53	≤ 65	absence	1	12	-1,02388	-0,26294	-0,4136	1,01	-0,11201	-0,18587	0,351579	-0,29222
28	21014	1-2	0	0	1-2	1-2	female	72	> 65	absence	0	71	-1,20005	-2,45679	-2,4568	1,4354	0,62347	1,967969	-0,48611	-0,14689
29	33597	1-2	1-2	0	3-4	1-2	female	73	> 65	absence	1	37	-0,00648	1,668992	1,1655	0,4247	2,79058	0,139037	1,266303	0,109288
30	33001	1-2	0	0	1-2	1-2	male	66	> 65	absence	1	41	-0,02245	-0,04396	-0,044	-0,9464	0,22961	-0,88228	-1,07368	0,378076
31	5276	1-2	0	0	3-4	1-2	female	56	≤ 65	absence	0	97	-1,66293	-0,57468	-0,4761	0,3583	-3,04961	-1,56866	0,350804	-1,25082
32	32998	1-2	0	0	1-2	1-2	male	68	> 65	absence	0	72	0,048735	-3,89563	-3,8956	-0,1427	-0,68213	-2,19011	-1,1387	0,303835
33	38368	1-2	0	0	1-2	1-2	female	52	≤ 65	absence	0	135	1,772696	-1,61554	0,0971	0,3065	-0,0854	-0,45427	-0,9857	-0,03753
34	36792	1-2	1-2	0	mixed	3-4	female	55	≤ 65	absence	1	10	-1,02335	0,708673	0,7087	-0,2148	-0,58991	-0,05671	1,182753	2,417267
35	32505	3-4	0	0	1-2	1-2	male	55	≤ 65	absence	0	9	0,16709	0,789772	-0,9808	5,2073	-3,22229	0,230305	-0,98419	-0,28461
36	16647	1-2	0	0	1-2	1-2	male	71	> 65	absence	1	77	0,601671	0,368239	0,3682	2,4861	-2,56181	0,82112	-1,07496	-0,57238
37	22088	3-4	1-2	0	3-4	3-4	male	65	≤ 65	absence	1	74	1,031834	0,661905	0,8152	-0,4686	-1,69504	0,392663	-0,43701	1,678775
38	6102	1-2	0	0	1-2	1-2	female	61	≤ 65	absence	0	132	-0,48777	-0,44844	-0,4484	0,43	-2,51558	-0,48803	0,337957	0,316298
39	5792	1-2	1-2	1	1-2	3-4	male	46	≤ 65	absence	1	59	0,539821	1,933735	1,9337	0,3096	0,41854	0,664206	2,004921	1,001493
40	7017	1-2	0	0	mixed	1-2	female	76	> 65	absence	0	156	0,774017	0,693848	-1,7946	0,0121	-0,02316	-1,83106	-0,30652	0,609384
41	10825	1-2	1-2	0	1-2	1-2	male	74	> 65	absence	0	108	1,125603	2,358408	2,3584	0,5901	0,54286	1,270768	2,620202	0,471278
42	6721	1-2	1-2	0	3-4	1-2	female	69	> 65	absence	1	54	0,820251	0,03441	-0,178	1,4971	-1,35595	-0,69852	0,07342	-0,19697
43	10556	1-2	1-2	0	1-2	1-2	female	71	> 65	absence	0	80	0,316436	0,068878	0,0689	-0,9702	1,92158	-0,45537	0,717648	1,828824
44	7472	3-4	0	0	1-2	1-2	male	49	≤ 65	absence	0	109	0,758143	0,546452	-0,5362	0,95	-6,19093	-2,82711	-2,405	0,295019
45	8467	1-2	1-2	0	1-2	3-4	female	38	≤ 65	absence	1	70	1,041356	1,720419	1,7204	-0,4976	0,907	0,086899	4,11799	0,69416
46	16154	1-2	1-2	0	1-2	3-4	male	59	≤ 65	absence	0	109	0,500656	1,739149	1,003	0,2411	1,71007	1,634078	1,466317	0,972625
47	16944	3-4	0	0	3-4	3-4	female	75	> 65	presence	1	7	0,334786	-2,27722	-2,2772	2,0441	-5,15556	-5,04279	-6,17695	0,407157
48	17778	1-2	0	0	1-2	1-2	male	67	> 65	absence	1	109	-0,24386	0,173963	-2,7969	0,5391	-8,07827	-7,5826	-4,37738	-0,11981
49	24359	3-4	1-2	0	1-2	3-4	male	65	≤ 65	absence	1	72	-1,67007	-1,11046	-1,1105	0,5313	-1,42902	0,204564	-0,09273	-0,0841
50	16659	1-2	0	0	1-2	1-2	female	58	≤ 65	absence	1	53	0,550504	2,697539	-0,5778	0,6124	-1,04481	-1,55732	-0,89589	0,773146
51	16678	3-4	0	1	1-2	3-4	male	60	≤ 65	absence	1	9	1,020105	-4,24289	-4,2429	-0,4034	-2,05237	-1,52216	-6,16205	1,266949
52	33537	3-4	1-2	0	3-4	3-4	male	28	≤ 65	absence	1	9	0,649058	1,755361	-1,1775	1,5984	-2,16038	-0,42906	-1,61473	-0,32461
53	37286	3-4	0	0	3-4	3-4	male	59	≤ 65	absence	1	94	-1,71541	-3,86616	-3,8662	0,4871	-0,91044	-2,86986	-3,8955	0,388924
54	36860	3-4	0	0	1-2	1-2	male	68	> 65	absence	1	45	-1,54059	-0,39639	0,0209	-0,3544	-0,06299	-1,05033	-0,24496	-0,23274
55	35493	1-2	0	0	mixed	1-2	female	77	> 65	absence	1	116	2,508781	0,891001	0,891	0,375	-0,02474	0,106498	0,500127	0,287288
56	36405	1-2	0	0	mixed	1-2	male	60	≤ 65	absence	1	15	1,576946	-3,46232	1,3077	-0,364	-0,80121	-0,16862	-2,84918	0,639628
57	8961	1-2	1-2	0	1-2	3-4	female	46	≤ 65	absence	1	34	-0,24446	-0,45293	-0,3033	-0,7026	0,75443	-0,65045	0,041657	-0,15431

58	32974	1-2	0	0	1-2	1-2	male	64	≤ 65	absence	0	110	0,080916	0,156479	0,1565	-0,9858	0,25806	-0,55018	-0,05635	0,788461
59	18050	1-2	0	0	1-2	1-2	female	75	> 65	presence	1	108	0,051296	-0,17981	0,1761	0,0742	0,3534	-0,83832	-0,06685	-0,06773
60	26599	3-4	1-2	0	1-2	3-4	male	66	> 65	absence	1	11	-0,83232	-0,13799	-0,138	-0,75	-0,37276	-1,43136	0,130192	0,376718
61	14324	1-2	1-2	0	3-4	3-4	male	69	> 65	presence	1	28	0,321014	1,010651	1,0107	-0,2993	-1,5915	-0,81756	-1,52203	-0,15254
62	17378	1-2	1-2	0	1-2	3-4	male	65	≤ 65	absence	1	16	1,070142	2,04076	1,884	-1,2257	0,50764	-0,47	-0,03529	0,892351
63	31507	3-4	1-2	0	3-4	3-4	male	46	≤ 65	absence	1	3	-0,00928	-0,15147	-0,1515	0,7115	0,81587	0,230821	-4,03278	-1,1581
64	21864	1-2	1-2	0	1-2	3-4	female	61	≤ 65	absence	1	6	0,358081	0,007499	0,1079	-0,6053	-0,41703	-0,46615	0,035018	-0,38403
65	4630	1-2	0	0	mixed	1-2	female	59	≤ 65	absence	1	114	1,467841	1,622536	0,2106	0,0419	-0,49288	-0,85247	0,561205	0,626079
66	5151	3-4	0	0	1-2	3-4	male	51	≤ 65	absence	1	46	-0,63035	1,951072	1,9511	0,1395	0,37739	2,84109	-0,00269	-0,45094
67	6014	1-2	1-2	0	mixed	3-4	female	48	≤ 65	absence	1	15	-0,41187	2,171384	0,9604	0,3266	0,14002	1,235457	2,592663	1,357083
68	33339	1-2	0	0	1-2	1-2	male	71	> 65	absence	1	68	4,776649	4,009638	4,0096	-2,02	-0,35437	2,762807	1,937319	0,827461
69	28645	1-2	0	0	1-2	1-2	male	55	≤ 65	absence	0	168	4,848875	3,734619	1,9593	-1,471	1,81292	1,768662	0,709627	0,948307
70	26426	1-2	0	0	mixed	1-2	male	67	> 65	presence	1	7	-0,14081	-0,74004	-0,74	-0,371	-1,79072	-0,9992	0,640299	0,27887
71	35410	1-2	0	0	1-2	1-2	female	68	> 65	absence	1	60	-0,67044	1,119317	1,438	0,0994	2,32194	0,422151	0,872326	1,237463
72	24385	1-2	1-2	0	1-2	3-4	female	56	≤ 65	absence	1	27	-0,41847	0,275338	0,2753	-0,6027	1,28746	-0,04071	-0,45317	0,360481
73	25581	1-2	1-2	0	1-2	3-4	male	59	≤ 65	absence	0	158	-0,52086	3,212833	-0,7044	0,0176	2,50005	0,249411	0,717879	-0,00033
74	25644	1-2	0	0	3-4	1-2	male	72	> 65	absence	1	84	3,256292	2,428454	2,4285	-0,0621	-0,42879	1,986438	0,535683	0,902637
75	24554	1-2	0	0	1-2	1-2	male	82	> 65	absence	1	23	1,074014	0,029385	-0,339	-0,1132	0,19543	1,632698	0,283478	-0,1898
76	20518	3-4	0	0	mixed	3-4	male	53	≤ 65	absence	1	25	1,444129	0,053981	0,054	0,302	0,84215	0,775742	-1,44378	-1,26354
77	21571	1-2	0	0	1-2	1-2	male	63	≤ 65	absence	0	135	1,725437	-0,81146	-1,3543	0,2119	0,29844	0,4232	-1,22696	-0,00229
78	26328	1-2	0	0	1-2	1-2	male	62	≤ 65	absence	1	141	-0,10442	0,770825	0,7708	0,9314	1,27699	1,056177	0,289757	0,248217
79	30757	3-4	0	0	1-2	1-2	male	60	≤ 65	absence	1	43	0,202982	2,012027	2,5767	0,2006	0,26286	-0,12409	-0,05951	0,130738
80	23875	1-2	1-2	0	3-4	3-4	female	63	≤ 65	absence	1	100	-0,14115	-0,3501	-0,3501	-0,0447	1,96193	0,122166	1,694076	1,236907

Table S2. MS analysis of bands demonstrated the abundance of protein complexes containing α, β, and δ hemoglobin subunits.

MS/MS View	Accession Number	Alternate ID	Molecular Weight	Protein Grouping Ambiguity	Fold Change by Category	220429 _N1	220429 _N2	220429 _N3	220429 _T1	220429 _T2	220429 _T3
Hemoglobin subunit beta OS=Homo sapiens OX=9606 GN=HBB PE=1 SV=2	P68871	HBB	16 kDa	TRUE	0,8	123	169	117	114	68	147
Hemoglobin subunit alpha OS=Homo sapiens OX=9606 GN=HBA1 PE=1 SV=2	P69905	HBA1	15 kDa		0,8	73	86	62	62	57	58
Hemoglobin subunit delta OS=Homo sapiens OX=9606 GN=HBD PE=1 SV=2	P02042	HBD	16 kDa	TRUE	0,8	65	92	65	60	36	77
Protein S100-A9 OS=Homo sapiens OX=9606 GN=S100A9	P06702	S100A9	13 kDa		1,2	55	56	37	63	57	62
Cluster of Keratin, type II cytoskeletal 1 OS=Homo sapiens OX=9606 GN=KRT1 PE=1 SV=6 (P04264)	P04264 [14]	KRT1	66 kDa	TRUE	1,1	61	44	57	62	54	59
Glyceraldehyde-3-phosphate dehydrogenase OS=Homo sapiens OX=9606 GN=GAPDH PE=1 SV=3	P04406	GAPDH	36 kDa	TRUE	1,1	46	53	58	64	62	43
Cluster of Tubulin beta chain OS=Homo sapiens OX=9606 GN=TUBB PE=1 SV=2 (P07437)	P07437 [7]	TUBB	50 kDa	TRUE	1	50	39	42	44	47	45
